# Modulation of lysosomal function as a therapeutic approach for coronaviral infections

**DOI:** 10.21203/rs.3.rs-419305/v1

**Published:** 2021-04-23

**Authors:** Yuan Liu, Travis Lear, Mads Larsen, Bo Lin, Qing Cao, Irene Alfaras, Jason Kennerdell, Laura Salminen, Daniel Camarco, Karina Lockwood, Jing Ma, Jie Liu, Jay Tan, Michael Myerburg, Yanwen Chen, Claudette St Croix, Yusuke Sekine, John Evankovich, Toren Finkel, Bill Chen

**Affiliations:** University of Pittsburgh; University of Pittsburgh; University of Pittsburgh; University of Pittsburgh; University of Pittsburgh; University of Pittsburgh; University of Pittsburgh; University of Pittsburgh; University of Pittsburgh; University of Pittsburgh; University of Pittsburgh; University of Pittsburgh School of Medicine; University of Pittsburgh; University of Pittsburgh; University of Pittsburgh; University of Pittsburgh; University of Pittsburgh School of Medicine; University of Pittsburgh School of Medicine; University of Pittsburgh; University of Pittsburgh

**Keywords:** coronaviral infections, SARS-CoV-2, lysosomal function

## Abstract

The endo-lysosomal pathway plays an important role in pathogen clearance and both bacteria and viruses have evolved complex mechanisms to evade this host system. Here, we describe a novel aspect of coronaviral infection, whereby the master transcriptional regulator of lysosome biogenesis – TFEB – is targeted for proteasomal-mediated degradation upon viral infection. Through mass spectrometry analysis and an unbiased siRNA screen, we identify that TFEB protein stability is coordinately regulated by the E3 ubiquitin ligase subunit DCAF7 and the PAK2 kinase. In particular, viral infection triggers marked PAK2 activation, which in turn, phosphorylates and primes TFEB for ubiquitin-mediated protein degradation. Deletion of either DCAF7 or PAK2 blocks viral-mediated TFEB degradation and protects against viral-induced cytopathic effects. We further derive a series of small molecules that interfere with the DCAF7-TFEB interaction. These agents inhibit viral-triggered TFEB degradation and demonstrate broad anti-viral activities including attenuating in vivo SARS-CoV-2 infection. Together, these results delineate a viral-triggered pathway that disables the endogenous cellular system that maintains lysosomal function and suggest that small molecule inhibitors of the E3 ubiquitin ligase DCAF7 represent a novel class of endo-lysosomal, host-directed, anti-viral therapies.

## Introduction

Autophagy plays a key role in the cellular response to microbial threats including a direct role for pathogen elimination through the endo-lysosomal pathway^1^. Master transcriptional control of autophagy rests with the Transcription factor EB (TFEB), which regulates genes involved in all stages of autophagy^2,3^. Various stressors stimulate the nuclear translocation of TFEB where it can activate >400 different gene products containing the CLEAR (coordinated lysosomal expression and regulation) regulatory motif^4^. These gene products work to increase lysosomal number and function, endocytic function, lysosomal exocytosis, and maintain lysosomal acidification^5^. TFEB’s role in host defense is evolutionarily conserved, as it is known to play a prominent role in the response to bacterial pathogens in organisms such as *C. elegans* ^6^. In many cases, pathogens have evolved sophisticated mechanisms to disrupt or evade the host’s autophagic machinery^7–10^. Here, we report that coronaviruses trigger TFEB protein degradation through E3 ligase-dependent ubiquitination. A small molecule that specifically blocks virally-induced TFEB degradation is shown to have potent anti-viral activity.

## Results

### Viruses induce proteasomal-mediated degradation of TFEB

**Figure 1:**
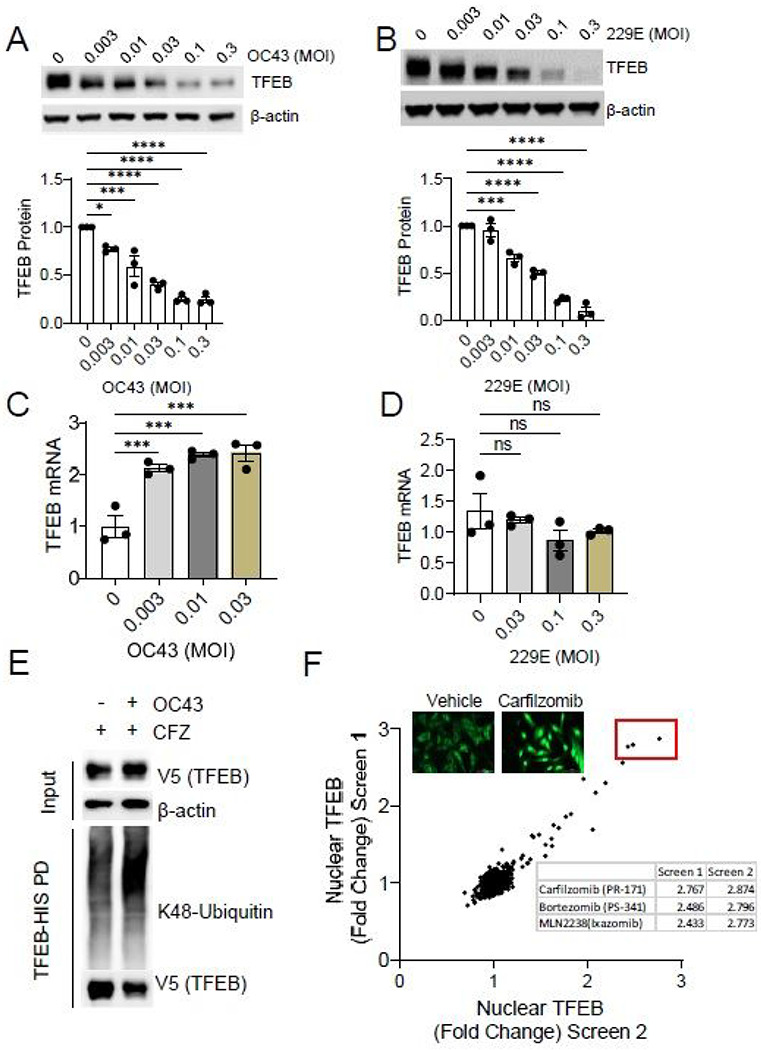
Viral infection triggers TFEB proteolytic degradation. A. Immunoblot analysis of human bronchial airway BEAS-2B cells infected with beta-coronavirus OC43 at the indicated MOI (48hr post inoculation). TFEB protein densitometry was quantified; data represent mean ±SEM (n=3). B. Immunoblotting of human lung fibroblast MRC-5 cells following alpha-coronavirus 229E infection (48hr after inoculation). TFEB protein densitometry was quantified; data represent mean ±SEM (n=3). C-D qPCR analysis of TFEB mRNA from BEAS-2B treated with increasing MOI of OC43 (F) or MRC-5 cells treated with increasing doses of 229E (G). Data represent fold change in TFEB mRNA level relative to control treatment; mean ±SEM (n=3). E. Immunoblot analysis of Lysine-48 linked polyubiquitination of TFEB protein in the presence or absence of OC43 infection. Lysates were generated from cells treated with the proteasomal inhibitor carfilzomib (CFZ). F. FDA-approved compound screening for enhancers of TFEB nuclear localization in the setting of coronavirus infection. TFEB-EGFP-BEAS-2B cells were infected with OC43 prior to treatment with a library of approximately 1100 FDA-approved drugs. Log-fold change in nuclear TFEB signal for each compound is shown for two independent screens. The top three hits (red box) identified from both screens are known proteasomal inhibitors. NS, p>0.05; *, p<0.05; **, p<0.01; ***, p<0.001; ****, p<0.0001 compared to vehicle or control or as indicated by one-way ANOVA with Dunnetťs multiple comparisons (A-D).

Recent observations have demonstrated that coronaviruses, including SARS-CoV-2, induce a critical deacidification of lysosomes, as part of their pathogenesis^10^. We were intrigued by this observation, since normally, lysosomal pH is kept in a narrow physiological range by the feedback, homeostatic activity of TFEB and related transcription factors ^11^. To begin to understand how coronaviruses are able to modulate lysosomal function, we inoculated human airway cells with two human coronavirus strains, OC43 and 229E; strains associated with the common cold^12^. Surprisingly, we observed a marked dose and time dependent reduction of TFEB protein level after viral infection (Fig. 1A–B, S1A–D). This fall in protein levels occurred without a concomitant reduction in TFEB transcription (Fig. 1C–D). Viral infection decreased the cytoplasmic and nuclear pools of TFEB protein, revealing a nuclear-compartment loss of TFEB protein and thus presumed impairment of its transcriptional activity (Fig. S1E–F). These results suggest that viral infection likely triggers the post-translational degradation of TFEB, which may impair the cell’s ability to maintain lysosomal function. In support of this, we observed that following viral infection, there was an increase in K48-linked TFEB poly-ubiquitin, the canonical linkage associated with proteasome degradation (Fig. 1E). To begin to understand whether this observation might be a point for future therapeutic intervention, we performed an unbiased, high throughput chemical screen using a library of FDA-approved molecules (approximately 1100 compounds). We used an EGFP-tagged TFEB chimeric protein, and specifically focused on nuclear TFEB levels in the setting of concurrent viral infection, as the nuclear pool of TFEB is responsible for its biological activity. From this screen, we noted that the top hits, namely molecules that could maintain nuclear TFEB levels in the setting of coronaviral infection, all represented chemical inhibitors of the ubiquitin-proteasomal system (UPS) (Fig. 1F). Together, these results suggest that coronaviral infection leads to reduced TFEB protein levels, likely mediated through the ubiquitin proteasomal pathway.

### The E3 ligase DCAF7 mediates TFEB degradation

**Figure 2:**
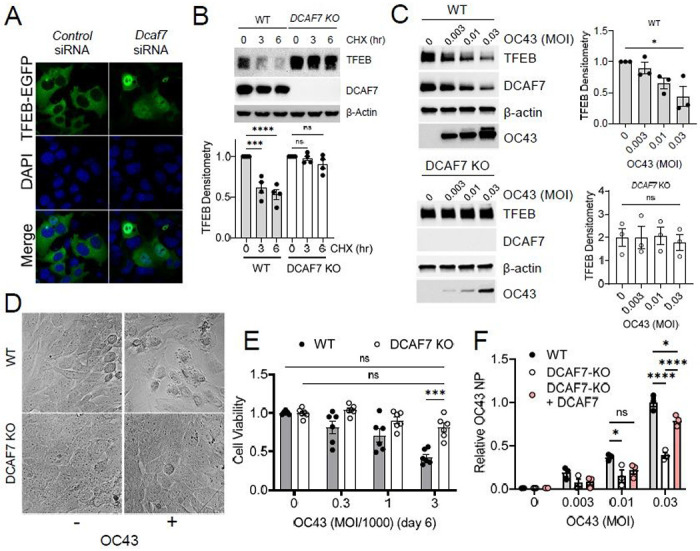
The E3 ubiquitin ligase DCAF7 regulates TFEB levels and modulates beta-coronavirus infection. A. Immunofluorescence microscopy of TFEB-GFP expressing BEAS-2B cells transfected with either a control or DCAF7 siRNA. B. Western blot analysis of a cycloheximide (CHX) chase in WT or DCAF7 knockout (KO) BEAS-2B cells demonstrating increased TFEB stability with DCAF7 deletion. TFEB protein densitometry was corrected to β-actin and normalized to time 0 control, data represent mean ±SEM (n=4). C. Immunoblot analysis of WT or DCAF7-KO BEAS-2B cells infected with the indicated MOI of OC43 for 48 h before analysis. TFEB protein densitometry for both WT and DCAF7 KO cells was corrected to β-actin and normalized to WT MOI=0 condition, data represent mean ±SEM (n=3). D. Representative phase microscopy for WT or DCAF7 KO BEAS-2B cells in the presence (+) or absence (−) of OC43 infection (72 h post infection). E. Quantification of cell viability at day 6 post OC43 infection. Data from CellTiterGlo2.0 readings were normalized to untreated control and represent mean ±SEM (n=6). F. OC43 nucleoprotein (NP) as measured by an in-cell ELISA in WT BEAS-2B cells, DCAF7-KO BEAS-2B cells, or DCAF7-KO BEAS-2B cells reconstituted with DCAF7 with increasing MOI of OC43. Data were normalized to WT MOI=0.03 treatment and represent mean ± SEM (n= 3). NS, p>0.05; *, p<0.05; **, p<0.01; ***, p<0.001; ****, p<0.0001 compared to vehicle or control or as indicated by one-way ANOVA with Tukey’s multiple comparisons (B, C), or two-way ANOVA with Tukey’s (E-F) multiple comparisons.

E3 ubiquitin ligases catalyze substrate ubiquitination, and we therefore sought to identify the E3 ubiquitin ligase regulating TFEB degradation. TFEB-EGFP pulldown (PD) and MS/MS analysis detected only one E3 ligase protein, DCAF7, present in the TFEB-EGFP PD samples (total 449 proteins detected) (Fig. S2A). DCAF7 is a substrate receptor for the Cul4-based CRL4 ubiquitin E3 ligase complex, and aside from a few studies, including a study validating its nuclear localization, the mechanistic function of DCAF7 remains unknown^13–15^. Ectopic DCAF7 expression decreased TFEB protein levels in a dose-dependent manner (Fig. S2B), and significantly increased TFEB poly-ubiquitination (Fig. S2C). Furthermore, RNAi knockdown of *DCAF7*led to an accumulation of TFEB protein in the nucleus (Fig. 2A). The bioinformatic tool, BDM-PUB, predicted a key ubiquitin acceptor within TFEB (Lys-232), which when mutated increased TFEB protein stability and reduced DCAF7-mediated ubiquitination (Fig. S2D–F)^16^. CRISPR-based gene editing of DCAF7 revealed that in *DCAF7*knockout (KO) cells, TFEB exhibited a significantly prolonged protein half-life (Fig 2B), as well as increased nuclear localization and reduced poly-ubiquitination, (Fig. S2G, S3A).

Prior to viral infection, *DCAF7* KO cells demonstrated an approximate 2-fold increase in the level of TFEB protein levels compared to WT control cells (Fig. 2C). This supports a role for DCAF7 control of TFEB levels under basal conditions. Following coronavirus challenge, TFEB protein declined in WT cells but was preserved in *DCAF7* KO cells (Fig. 2C, S3B). Interestingly, *DCAF7* KO cells were also resistant to the cytopathic effects of OC43 infection (Fig. 2D) and demonstrated an increase in viability after infection (Fig. 2E, S3C). Preservation of TFEB also correlated with a significant decrease in viral load (Fig. S3B, D).

These observations appear relevant to other viral pathogen families, as H1N1 influenza infection of airway epithelial cells also led to a dose-dependent reduction in TFEB protein which was blunted in *DCAF7* KO cells (Fig. S2E). To validate the fidelity of our KO cells, we re-expressed DCAF7. Reconstitution of DCAF7 in *DCAF7* KO cells abrogated the protective KO phenotype, demonstrating comparable viral infection as WT cells (Fig. 2F, S4A–B).

### PAK2 primes TFEB for DCAF7-dependent degradation

**Figure 3:**
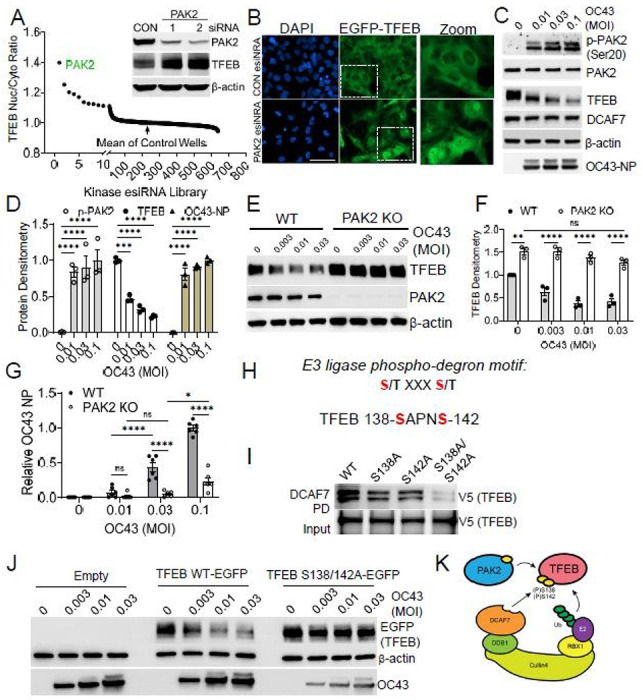
Viral infection activates PAK2 kinase to prime TFEB for degradation. A. High content screening of kinase regulators of TFEB-EGFP nuclear localization. BEAS-2B cells stably expressing TFEB-EGFP were screened with an RNAi library targeting all human kinases prior to quantification of TFEB nuclear to cytosolic ratio. The kinase PAK2 was identified as a top regulator of TFEB nuclear levels and confirmed by immunoblotting of PAK2 siRNA treatment in BEAS-2B cells (inset). B. Representative images of TFEB-EGFP localization following control or PAK2 RNAi treatment. PAK2 siRNA increased nuclear TFEB levels. Boxed area is shown at higher magnification (Zoom). Scale bar = 50 μm. C. Immunoblot analysis demonstrating PAK2 activation (Serine-20 phosphorylation) in BEAS-2B following OC43 infection (48h), with the subsequent decline in TFEB levels and rise in OC43 nucleoprotein (OC43-NP). D. Quantification of protein levels from experimental design in (C). Indicated protein densitometry was corrected to unmodified protein or β-actin and normalized to highest or lowest MOI treatment. Data represent mean ±SEM (n=3). E. Immunoblot analysis of TFEB levels in WT or PAK2 knockout (KO) BEAS-2B cells following infection with OC43 at the indicated MOI (48h). F. Quantification of TFEB protein level from experimental design in panel (E). TFEB protein densitometry was corrected to β-actin and normalized to WT-control. Data represent mean ±SEM (n=3). G. Quantification of viral infection through an in-cell ELISA assay of WT or PAK2 KO BEAS-2B cells infected with OC43 (48h). Data were normalized to the WT MOI=0.1 treatment levels and represent mean ±SEM (n=6). H. A canonical E3 ligase phospho-degron motif 21 S/T XXX S/T is present within TFEB at serine-138 and serine-142. I. Binding assay of TFEB serine-alanine mutants with DCAF7. In vitro synthesized V5-tagged TFEB mutants were incubated with immunoprecipitated DCAF7 prior to washing, elution, and immunoblot analysis. J. Immunoblot analysis of BEAS-2B cells transfected with either WT TFEB or the double serine phospho-mutant TFEB prior to OC43 infection (72h). The phospho-mutant is resistant to degradation and reduces OC43 NP expression. K. Schematic of PAK2-induced priming phosphorylation required for DCAF7-mediated poly-ubiquitination. NS, p>0.05; *, p<0.05; **, p<0.01; ***, p<0.001; ****, p<0.0001; compared to vehicle or control or as indicated by two-way ANOVA with Tukey’s multiple comparisons (D, F-G).

The Cullin based E3 ligase complex typically targets phosphorylated substrates^17–19^, and TFEB translocation is known to be regulated by phosphorylation status^5^. Taken together, we hypothesized that viral infection might alter the phosphorylation of TFEB, marking the protein for ubiquitination. We used a siRNA library targeting all 600 kinases and high content imaging to identify regulators of TFEB nuclear localization. This unbiased approach uncovered p21-activated kinase 2 (PAK2) as a key determinant of TFEB localization and stability (Fig. 3A–B). OC43 coronavirus infection dramatically increased PAK2 activation (phospho-serine-20), which correlated with a decline in TFEB levels and a rise in viral load (Fig. 3C–D). Deletion of PAK2 increased basal TFEB levels and significantly mitigated the viral-induced TFEB protein decline (Fig. 3E–F). Similarly, PAK2 KO cells displayed reduced viral load and improved overall cell viability (Fig. 3G, S5A).

We next explored the mechanism whereby PAK2 modulates TFEB stability. Through protein binding assays we noted that PAK2 and TFEB could directly interact, and we defined a critical region within TFEB’s basic helix-loop-helix domain required for PAK2 binding (Fig. S5B–D). We sought to uncover the key substrate phosphorylation sites, *phospho-degrons*, within TFEB that may serve as E3 ligase engagement motifs^20^. We hypothesized that proteasomal inhibition would specifically enrich for TFEB phosphorylation sites that might act as phospho-degrons, identifiable through phospho-proteomics (Fig. S6A). We identified several TFEB phosphorylation sites significantly enriched with proteasomal inhibition (MG132), making them candidate phospho-degrons (Supplemental Table 1). Of these candidates, serine-138 and serine-142 fit the pattern of a classical E3 ligase phospho-degron motif: S/T XXX S/T^21^ (Fig. 3H). Mutation of each of these candidate serine residues to alanine resulted in reduced DCAF7-TFEB interaction (Fig. 3I). The double serine to alanine mutant (S138A/S142A), hereafter termed the TFEB phospho-mutant, further weakened this interaction when compared to mutation of each residue alone, and was resistant to DCAF7-mediated ubiquitination (Fig. S6B). We next explored the functional importance of this mechanism. The TFEB phospho-mutant exhibited enhanced nuclear localization and resistance to OC43 mediated TFEB phosphorylation (Fig. S6C–D). Further, the TFEB phospho-mutant was resistant to degradation following OC43 infection and its persistent expression decreased viral load (Fig. 3J, S6E–F). Together, this suggests PAK2-mediated TFEB phosphorylation generates a critical molecular motif for substrate engagement by the DCAF7-E3 ligase complex (Fig. 3K)

### Small molecule DCAF7 inhibitors modulate *in vitro* and *in vivo* TFEB activity

From these data, we hypothesized that small molecule inhibition of PAK2 or DCAF7 could prevent TFEB protein degradation, leading to preserved endo-lysosomal activity and the potential to improve the cell’s anti-viral response. To date, it has been difficult to generate PAK2 or PAK family inhibitors without toxicity^22,23^. As such, we focused on novel small molecules targeting the E3 ubiquitin ligase DCAF7. DCAF7 harbors a conserved WD repeat domain within its C-terminus which is a key substrate binding domain ^24,25^. We hypothesized that small molecule inhibition of the WD repeat domain would disrupt DCAF7’s interaction with TFEB. We constructed a DCAF7 homology model using the Nurf55 WD domain crystal structure (2XYI.pdb)^26^ and screened 3 million compounds (ChemDiv, INC) as potential ligands *in silico* (Fig. S7A). The top score-ranking molecules were selected and further evaluated *in vitro.* The initial top hit, termed BC1753, dose-dependently increased TFEB protein, and nuclear localization, and increased autophagosome formation and lysosomal activity (Fig. S7B–E). We executed multiple rounds of hit-to-lead and subsequent lead optimization and arrived at two closely related candidate DCAF7 inhibitors, BC18813 and BC18630 (Fig. S7F). Direct target engagement was confirmed through a thermal shift assay (Fig. S8A).

**Figure 4:**
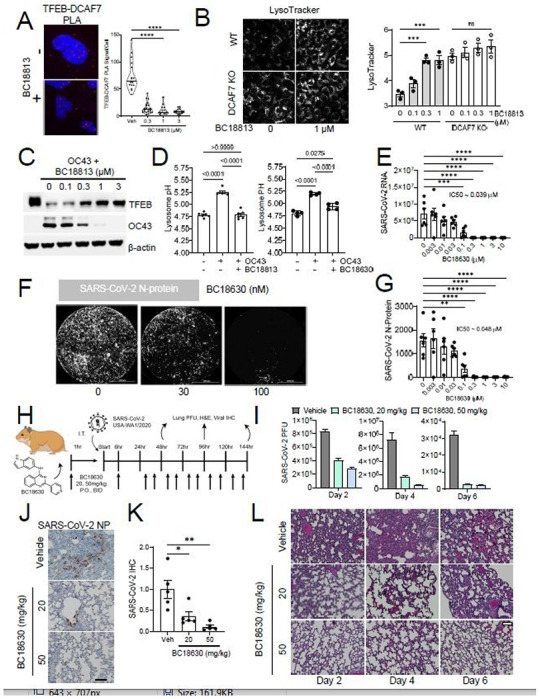
Small molecule DCAF7 inhibitors are protective against coronaviral infection, including SARS-CoV-2, in vitro and in vivo. A. Proximity Ligation Assay (PLA) of TFEB and DCAF7. DCAF7 inhibitors decreased TFEB-DCAF7 association in the nucleus. Data represent mean and interquartile range in violin plot (n=11-25 cells). B. Fluorescent micrograph of WT or DCAF7 KO BEAS-2B cells treated with BC18813 (18hr) and stained with Lysotracker, a measure of lysosomal number. Lysotracker fluorescence was quantified, data represent median lysotracker signal from each sample, mean ±SEM (n=3). C. Immunoblot analysis of OC43 infected BEAS-2B cells treated with BC18813 demonstrates maintenance of TFEB levels and reduced viral load with increasing compound concentration. D. Quantification of lysosomal pH in BEAS-2B cells infected with OC43 for 7hr without or with 18hr pre-treatment of BC18813 (50 nM) or BC18630 (100 nM). Data represent mean ±SEM (n=4). E-G. Cell-based SARS-CoV-2 infection assay. Calu-3 human lung cells were pre-treated with BC18630 for 4hrs before inoculation with SARS-CoV-2 virus (USA-WA-1/2020, MOI=0.01). After a 75 min inoculation, cells were washed and media was replaced with compound at the indicated concentration. Samples of the supernatant were taken 48 hours later to quantify viral RNA levels. Data in panel E represents mean ± SEM (n=6), and IC50 values were determined by sigmoidal nonlinear regression. Cells were fixed and stained for SARS-CoV-2 nucleoprotein (NP) for quantification of viral signal (F-G). Data represent mean ± SEM (n=6), and IC50 values were determined by sigmoidal nonlinear regression. H. Schematic of in vivo SARS-CoV-2 hamster infection model. Briefly, 5 Syrian golden hamsters per group were treated with BC18630 (P.O.) at the indicated doses 1 hour prior to inoculation with SARS-CoV-2 (i.t.). Six hours later, a second dose of BC18630 was administered and thereafter animals were treated BID for 5 days. Animals were sacrificed at days 2,4, and 6 for analysis. I. SARS-CoV-2 viral load determination using PFU assays at day 2, 4, and 6 post -infection from pooled lungs lysate of n=3 animals. Data represent mean ± SEM of technical replicates (n=3). J. Lung samples from 2 animals at day 4 post infection were stained for immunohistochemical detection of SARS-CoV-2 NP expression in tissues. K. Semi-quantification of IHC staining in panel (F). Relative SARS-CoV-2 NP signal was quantified from five random fields per treatment and normalized to vehicle treatment; mean ± SEM. L. Lung samples from 2 animals at day 2, 4, 6 post infection were stained for H&E staining to evaluate lung inflammation. Scale bar represents 100 μm. Ns, p>0.05; *, p<0.05; **, p<0.01; ***, p<0.001; ****, p<0.0001; as indicated by one-way ANOVA with Dunnetťs multiple comparisons (A, E, G, I, K), or with Tukey’s multiple comparisons (B, D).

Both BC18813 and BC18630 decreased TFEB ubiquitination and significantly reduced the measured interaction between DCAF7 and TFEB in nucleus of cells (Fig. 4A, S8B–D). Consistent with a role for DCAF7 in basal TFEB regulation, treatment with either DCAF7 inhibitor significantly increased nuclear levels of TFEB in a time and dose-dependent manner (Fig. S9A–H) in a wide variety of cell types (Fig. S10A–D). DCAF7 inhibition also resulted in a significant dose and time-dependent increase in multiple key TFEB-transcription targets (lysosomal proteases, autophagic transport, autophagy receptors) (Fig. S11A–D). To further validate our DCAF7 inhibitors, we treated *DCAF7* KO cells with our compounds, and observed no further increase in nuclear TFEB level (Fig. S12A–B). Similarly, while DCAF7 inhibitor treatment increased lysosomal number (Lysotracker) and activity (Magic Red) in WT cells, *DCAF7* KO cells were insensitive to these agents (Fig. 4B, S12C–H). To assess the specificity of these compounds, we also tested their effects on known protein substrates of other closely related E3 ligases to DCAF7; DCAF7 inhibitors selectivity increased nuclear TFEB protein levels, without altering other substrates (Fig. S13A). In addition, both compounds exhibited minimal cellular toxicity at concentrations below 10 μM (Fig. S13B–C). We found that BC18630 displayed a superior pharmacokinetic profile in rodents compared to BC18813, and that BC18630 appeared to increase hepatic endo-lysosomal capacity in mice, as measured by dextran cascade blue uptake^27–29^ (Fig. S14A–C). Thus, these small molecule inhibitors of DCAF7 can function to increase basal TFEB levels and lysosomal activity *in vitro* and *in vivo*.

### DCAF7 inhibitors attenuate a wide range of viral infections including SARS-CoV-2 infection

As our DCAF7 inhibitors demonstrated the capacity to modulate basal TFEB levels and lysosomal activity, we next sought to explore the utility of these compounds in the setting of viral infection. DCAF7 inhibitors prevented virally-triggered TFEB degradation and dose-dependently reduced OC43 coronavirus infection (Fig. 4C, S15A). Consistent with previous observations^10^, coronavirus infection led to a significant increase in lysosomal pH (Fig. 4D). However, both BC18813 and BC18630 inhibited this virally-induced alkalization (Fig. 4D). These agents appeared to be relatively potent as we estimated that the reduction of HCoV-OC43 NP expression occurred at an IC50 of roughly 0.3 μM for both inhibitors (Fig. S15B, C). Further, both DCAF7 inhibitors strongly decreased the proportion of OC43 positive cells, (Fig. S15D, E). DCAF7 inhibitors also protected against 229E induced infection and cytopathic effects (Fig. S15F–J). Further, BC18813 demonstrated efficacy in maintaining TFEB protein level in the setting of infection with the structurally unrelated influenza virus, HI N1 (Fig. S15L).

Given the efficacy of this strategy to treat several relatively common coronaviruses, we next assessed the effect of modulating TFEB in the setting of live SARS-CoV-2 infection. We utilized the BSL3 facilities at the IIT Research Institute to test DCAF7 inhibitors in SARS-CoV-2 infection assays with Calu-3 cells. Both compounds significantly reduced SARS-CoV-2 viral RNA load and cellular infection as measured by SARS-CoV-2 nucleoprotein immunofluorescence (Fig. 4E–G, S16A–C). To further characterize the utility of our DCAF7 inhibitors, we next assessed an *in vivo* model of SARS-CoV-2 infection. We chose to study BC18630, given its favorable pharmacokinetic profile (Fig. 14A–C). We employed a Syrian hamster model of SARS-CoV-2 infection and treated with vehicle or two doses of BC18630 (oral BID at 20mg/kg and 50 mg/kg for 5 days) (Fig. 4H). We observed that BC18630 dose-dependently reduced lung viral titer (PFU) at all time points (Fig. 4I). The higher dose strategy produced a >90% viral load reduction at day 4 and 6. Immunohistochemical staining of lung slices for SARS-CoV-2 N-protein (NP) at day 4 post infection revealed significantly reduced epithelial NP-positive signal with BC18630 treatment (Fig. 4J–K). BC18630 also reduced the observed level of lung injury, with the higher dose particularly reducing inflammatory infiltrates (Fig. 4L). In summary, BC18630 appears to decrease viral titer, reduce viral protein abundance, and prevent adverse pathophysiological changes in the lungs of hamsters infected with SARS-CoV-2. These data demonstrate the utility of pharmacological-based methods to modulate TFEB levels as an anti-viral strategy.

## Discussion

Previous studies have demonstrated that many viruses have evolved mechanisms to circumvent or hijack the endo-lysosomal pathway^1,30,31^. Our data demonstrates that viral infection induces the proteasomal mediated degradation of TFEB. We observed that PAK2 activation is a critical priming step for TFEB degradation, as this kinase is activated by viral infection, generating a phospho-degron required for subsequent DCAF7 recognition (see model in Fig S17). Other groups have independently characterized the PAK kinase family, including PAK2, as associated with viral infectivity, including recent studies with SARS-CoV-2^32,33^. Intriguingly, either PAK2 deletion or genetic/pharmacological DCAF7 inhibition ablated viral-induced TFEB degradation, thereby maintaining lysosomal fitness and improving the host response. While our study has concentrated on TFEB during infection, the efficacy of DCAF7 inhibitors to modulate TFEB activity under basal conditions suggests potential application of this approach for other, non-infectious conditions associated with impaired auto-lysosomal activity. Indeed, pharmacological activation of TFEB represents an attractive target for a growing number of diseases^5,34–36^.

DCAF7 inhibitors showed efficacy against SARS-CoV-2 in cell and hamster-based infection models. With the rapid spread of SARS-CoV-2, and the high burden of acute respiratory failure, there is an urgent need for additional off-the-shelf therapies. Several small molecules have been investigated but each has drawbacks. For instance, remdesivir showed only modest clinical effects, that were most pronounced in severely ill patients ^37,38^. In contrast to viral-centered interventions, modulators of the DCAF7-TFEB-endo-lysosomal axis function to augment intrinsic cellular defense mechanisms. One appeal of this strategy is that it should hypothetically remain efficacious against future novel viral strains that might emerge. Uninvestigated in this study is the immunological consequences of TFEB augmentation. Increased lysosomal fitness among the sentinel epithelial compartment may also enhance antigen presentation generating a more potent immune-mediated clearance response. Taken together, our observations demonstrate a novel mechanism of TFEB regulation and reveal a host-centric, endo-lysosomal focused strategy to limit viral infections.

## Supplementary Material

1
