# Supplementary material for: Modulation of lysosomal function as a therapeutic approach for coronaviral infections
Source: Res Sq. 2021 Apr 23:rs.3.rs-419305. Preprint. [Version 1] doi: 10.21203/rs.3.rs-419305/v1 (PMC8132244; doi:10.21203/rs.3.rs-419305/v1)
Supplement: 1 [file 5ba6371a56f93e76eaf94a0b.pdf]

Supplemental Data

Modulation of lysosomal function as a therapeutic approach for coronaviral infections

Travis B. Lear[1,2*], Mads B. Larsen[1*], Bo Lin[1*], Qing Cao[1], Irene Alfaras[1], Jason R. Kennerdell[1], Laura Salminen[1], Daniel P. Camarco[1], Karina C. Lockwood[1], Jing Ma[3], Jie Liu[1], Jay X. Tan[1], Michael M. Myerburg[3], Yanwen Chen[1], Claudette St Croix[4], Yusuke Sekine[1], John W. Evankovich[1,3], Toren Finkel[1,2,5#], Bill B. Chen[1,2,3#] and Yuan Liu[1,3#]

[1] Aging Institute, University of Pittsburgh/UPMC, Pittsburgh, PA 15219, USA
[2] Vascular Medicine Institute, University of Pittsburgh, Pittsburgh, PA, 15213, USA
[3] Department of Medicine, Division of Pulmonary, Allergy and Critical Care Medicine, Acute Lung Injury Center of Excellence, University of Pittsburgh, Pittsburgh, PA 15213, USA
[4] Center for Biologic Imaging, University of Pittsburgh School of Medicine, University of Pittsburgh, Pittsburgh, PA 15261, USA
[5] Department of Medicine, Division of Cardiology, University of Pittsburgh, Pittsburgh, PA 15213, USA

*These authors contributed equally to this work

Address Correspondence to:
Yuan Liu, PhD #
Bill B. Chen, PhD #
Toren Finkel, MD, PhD #
University of Pittsburgh
Aging Institute, Department of Medicine
558 Bridgeside Point 1
100 Technology Drive
Pittsburgh, PA 15219
E-mail: yul119@pitt.edu, chenb@upmc.edu, finkelt@pitt.edu

Methods

CONTACT FOR REAGENT AND RESOURCE SHARING

Please contact Y.L. (yul119@pitt.edu) for reagents and resources generated in this study.

EXPERIMENTAL MODEL AND SUBJECT DETAILS

Cell Culture— BEAS-2B and MLE-12 cells from ATCC were cultured in HITES media supplemented with 10% fetal bovine serum (FBS). HCT-8 (ATCC® CCL-244™) cells were cultured in RPMI 1640 medium (Gibco, ATCC modification, A1049101) supplemented with 10 % FBS (Gibco) and Penicillin-Streptomycin (Gibco, 10, 000 U/ml, 15140163). MRC-5 (ATCC® CCL-171™) and MDCK (ATCC® CCL-34™) cells were cultured in Eagle's Minimum Essential Medium (EMEM) (ATCC® 30-2003™) supplemented with 16 % FBS and Penicillin-Streptomycin. Knockout cell lines were prepared using lentivirus. Sequences for target genes were created with GPP sgRNA Designer[2] and cloned into pLENTI-CRISPR-vs2[3]. Lentiviral particles were generated by co-expression of sgRNA encoded pLENTI-CRISPR-vs2 with psPAX2 and pMD2.G in HEK293T. Target cells were incubated with lentivirus prior to antibiotic selection and generation of monoclonal populations. Validation of knockout was determined by immunoblotting (See Supplemental Table 2 for list of antibodies and other materials). Cells were treated with compound at indicated doses for indicated times, or at the following doses: Carfilzomib, 1μM; Leupeptin, 1μM; Bafilomycin A1, 1μM. Cytosolic and Nuclear fractions were separated using NE-PER Nuclear and Cytoplasmic Extraction Reagents (Thermo).

Viral Propagation and Inoculation— The propagation methods and times are based on ATCC recommendations.

Human coronavirus OC43, Betacoronavirus 1, ATCC® VR-1558™, LOT: 70034234

HCT-8 cells were cultured on T75 flasks to 90 % confluence. The growth media was removed, and the cells were washed twice with serum free medium (RPMI 1640 medium with Penicillin-Streptomycin). 300 ul of the stock OC43 was diluted in 5 ml of serum free medium, and the virus dilution was adsorbed on the cells for 1 h at 34 °C with 5% CO_2. The adsorption was ended by adding 10 ml of serum free medium on the cells, and the virus was propagated for five days at 34 °C with 5% CO_2. The viral supernatant was collected by centrifugation at 1000 g for 10 min RT. To propagate more virus after the stock solution finished, 1 ml of the supernatant was added in 9 ml of serum free medium for adsorption on a 90% confluent T175 flask of cells, and it was ended by adding 20 ml serum free medium per flask and propagated as previously described.

Human coronavirus 229E, ATCC® VR-740™, LOT: 70034235

MRC-5 cells were cultured on T75 flasks to 90 % confluence. The growth media was removed, and the cells were washed twice with serum free medium (EMEM with Penicillin-Streptomycin). 300 ul of the stock 229E was diluted in 5 ml of serum free medium, and the virus dilution was adsorbed on the cells for 1 h at 34 °C with 5% CO_2. The adsorption was ended by adding 20 ml of serum free medium on the cells, and the virus was propagated for four days at 34 °C with 5% CO_2. The viral supernatant was collected by centrifugation at 1000 g for 10 min RT. To propagate more virus after the stock solution finished, 1 ml of the virus dilution was added in 9 ml of serum free medium for adsorption on a 90% confluent T175 flask of cells, and it was ended by adding 20 ml serum free medium per flask and propagated as previously described.

Cell Inoculation

Compound and virus dilutions were prepared in Dulbecco's Modified Eagle Medium (DMEM low glucose) (Gibco, 11885092) with 2 % FBS (Gibco) and Penicillin-Streptomycin (Gibco). Occasionally, specific medium for each cell line (RPMI/EMEM) was used with 2 % FBS and

Penicillin-Streptomycin for viral treatments. The calculated MOI of the virus was noted in experiment.

In vivo PK study of DCAF7 inhibitor— Pharmacokinetic studies of DCAF7 inhibitors were conducted at Touchstone Biosciences. Compounds were assayed in Sprague-Dawley rats. Male SD Rats were fed a standard laboratory rodent diet and housed in individual cages on a 12-hour light and 12-hour dark cycle with room temperature maintained at 22 ± 3°C and relative humidity at 50 ± 20%. Animals were typically fasted overnight before dosing, with food returned after the 6-hour blood samples are obtained. Water was provided ad libitum throughout the study. Animals were dosed via gavage needle for oral administration at 10-20 mg/kg (10 mL/kg) or via tail vein injection for intravenous administration at 2-5 mg/kg (2-5 mL/kg). All blood samples (30-80 µL per sample) were taken via appropriate vein (saphenous or jugular vein) at 5, 15, and 30 min and 1, 2, 4, 6, 8, and 24 h after dosing. Blood samples were collected in Greiner MiniCollect K2EDTA tubes, placed on ice, and within 30 minutes, centrifuged at 15,000 g for 5 min to obtain plasma samples. All plasma samples were prepared as follows. Three volumes of acetonitrile containing internal standard were added to one volume of plasma to precipitate proteins. Samples were centrifuged (3,000 g for 10 min) and supernatant removed for analysis by LC-MS/MS. Calibration standards and quality controls were made by preparation of a 1 mg/mL stock solution and subsequently a series of working solutions in methanol: water (1/:1, v/v) which were spiked into blank plasma to yield a series of calibration standard samples in the range of 1 ng/mL to 10 µg/mL and quality control samples at three concentration levels (low, middle and high). All incurred PK/PD plasma samples were treated identically to the calibration standards and quality control samples. LC-MS/MS analysis was performed utilizing multiple reaction monitoring for detection of characteristic ions for each drug candidate, additional related analytes and internal standard. Compound plasma concentrations were measured to determine a concentration vs. time profile. The area under the plasma concentration vs time curve (AUC) was calculated using the linear trapezoidal method. Fitting of the data to obtain pharmacokinetic parameters is generally carried out using non-compartmental analysis. All parameters were expressed for individual animals as well as mean, standard deviation, and coefficient of variation.

Murine model of Hepatic Lysosomal Activity — Lysosomal activity in mice was determined through dextran cascade blue uptake [4,5]. All procedures were approved by the University of Pittsburgh Institutional Animal Care and Use Committee. 10-12 week old C57BL/6 mice were given BC18630 (50 mg/kg, daily for four days) through i.p injection. 2 h after the last dose, mice were i.v. injected with dextran cascade blue as previously described[5]. Mice were then euthanized, and liver tissue were fixed, sectioned and imaged for dextran cascade blue fluorescence and stained for LAMP1 (lysosome).

In vitro SARS-CoV-2 infection assay— SARS-CoV-2 infectivity was assayed through infection of Calu-3 at the CRO IIT Research Institute (Chicago, USA). Human lung cancer Calu-3 cells were maintained in Eagle's Minimum Essential Medium with 10% FBS. Cells were cultured in a 96-well plate until reaching 80-90% confluency. Test articles were tested against wildtype USA-WA1/2020 (SARS-CoV-2) in six replicates. Compounds were serially diluted, and cells pretreated for 24 hours. After pretreatment, the test articles were removed and then incubated with a standardized virus concentration at 37°C ± 2°C in 5.0% +/- 1% CO2 for 75 ± 15 min. Following the 75 ± 15 min incubation, virus inoculum was removed, cells washed, and appropriate wells overlaid with test or control articles in 0.2 mL EMEM2 (EMEM with 2% FBS) and incubated in a humidified chamber at 37°C ± 2°C in 5 ± 2% CO2. At 48 ± 6 and 72 ± 6 hours post inoculation, from each plate, 120 µl of the supernatant from each well was collected for subsequent analysis by real-time PCR. The concentration of virus in the cell culture supernatants was determined by a real-time PCR assay. Briefly, samples kept at ≤ -65°C were thawed and centrifuged to remove cellular debris. RNA was

extracted using the Quick-RNA Viral Kit (Zymo Research) according to the manufacturer's protocol. Real-time PCR was performed using the following PCR cycling conditions: 50°C for 15 min (RT), then 95°C for 2 min (denature), then 40 cycles of 10 s at 95°C, 45 s at 62°C. Virus titer by real-time PCR was performed according to IITRI SOPs.

In vivo SARS-CoV-2 infection assay— SARS-CoV-2 infectivity was assayed through Syrian Golden Hamster models of infection. ChemDiv Inc was contracted to conduct *in vivo* SARS-CoV-2 infection assays. Animals were allowed to acclimate to their environment for two weeks prior to the assay. Briefly, BC18630 was given in two doses, 20 and 50 mg/kg and was administered PO, twice a day, for 5 days. The first dose of the drugs was administered 1 hour before SARS-CoV-2 infection (10^3 CPE50 of SARS-CoV-2 virus at 100 µl per animal), the next dose was given 6 hours after infection. Hamsters were euthanized at day 2, 4 and 6. Lungs were collected, and the antiviral activity was calculated based on the viral titer (using PFU assay) of pooled lung samples. Lungs were also collected, fixed and stained for pathology analysis.

METHOD DETAILS

Immunoblotting—Cells were lysed in RIPA buffer supplemented with EDTA-free protease inhibitor tablet on ice. Cell lysates were sonicated at 20% amplification for 12 seconds and centrifuged at 12,000 g for 10 minutes at 4°C. Supernatants were collected and normalized for the total protein concentrations, mixed with 6X protein sample buffer, and incubated at 42°C for 10 minutes. Sample lysate was resolved using 4-20% acrylamide PROTEAN® TGX™ precast gels from Bio-Rad and electrophoresed in Tris-Glycine-SDS (TGS) buffer. The proteins were then electro-transferred to nitrocellulose membranes. Blots were incubated in 15 ml of blocking buffer for 1 h at room temperature, before incubation in 10 ml of the primary antibody solution (1:1000 dilution) overnight at 4°C. Afterwards, three 10-minute washes were performed in 15 ml Tris-buffered saline + 0.05% Tween-20 (TBST). Blots were then incubated with 10 ml of the secondary antibody solution for 1 h at room temperature. After three 10-minute washes in 15 ml TBST, blots were then developed using West Femto Maximum Sensitivity Substrate from Thermo Scientific, and imaged using ChemiDoc Imaging System from Bio-Rad.

Real-time PCR— Total RNA was extracted using the RNA Extraction Miniprep Kit from Bioland Scientific, or using RNeasy Plus Mini Kit from Qiagen following the manufacturer's protocol. cDNA was prepared using the High-Capacity RNA-to-cDNA Kit from Applied Biosystems. SYBR Green Real-Time PCR Master Mixes from Applied Biosystems were used in qPCR. Primer sequences are detailed in the oligonucleotide table (Supplemental Table 3).

High-Throughput Liquid Handling— A Thermo Scientific custom HTS platform and Agilent Bravo automated liquid-handling platform were used to transfer contents of an FDA-approved compound library into assay plates. Biotek EL406 washer dispenser was used to distribute reagents or cell solutions into assay plates. For multiple plates operation, the plate and liquid handling sequence and intervals were controlled through the Agilent VWORKs software.

FDA-Approved Compound Library Screening— TFEB-EGFP BEAS-2B cells were seeded to a final density of 2500 cells per well in black 384 well plate with glass bottoms. Cells were treated with OC43 for a 24h period before exposure to the FDA compound library. The FDA-approved compound library (Selleck, 100nL per drug) was stamped to 384 well plate using CyBio Well vario (Analytik Jena). Compounds were then added to cells with the final concentrations of 2 µM. After 18 h of treatment, cells were then fixed in 4% paraformaldehyde, counterstained with DAPI, and

fluorescence was detected using Cytation5 High Content Imager (BioTek). Nuclear TFEB fluorescent signal was calculated using Gen5 software (BioTek).

Plasmid Preparation and Cloning— TFEB and DCAF7 coding sequences were subcloned into pcDNA3.1D-V5-HIS (Invitrogen) through TOPO cloning. Point mutants (Lysine→Arginine; Serine→Arginine) were generated through QuikChange II XL Site-Directed Mutagenesis Kit (Aglient). pRK5-HA-Ubiquitin-WT was a gift from Ted Dawson (Addgene plasmid # 17608; RRID:Addgene_17608 [6]). All plasmid constructs were verified by DNA sequencing (Genewiz).

Plasmid Transfection— Plasmid transfections were conducted using nucleofection in BEAS-2B and MLE-12 cells using Nucleofector II (Amaxa). X-tremeGENE HP DNA transfection reagent or Lipofectamine 3000 transfection reagent was used for plasmid transfections.

Ubiquitination Assay — TFEB-V5-HIS WT and mutants in pcDNA3.1D or TFEB-EGFP were co-expressed with HA-Ubi and DCAF7 in BEAS-2B cells for 18hr, prior to proteasomal inhibition where indicated, lysis and precipitation with Dynabead HIS-resin (Thermo) or GFP-Trap beads (ChromoTek). Precipitate was eluted in 1x Laemmli Protein Sample Buffer at 95°C for 10 min and resolved through SDS-PAGE immunoblotting.

TFEB Mass Spectrometry— TFEB-EGFP MLE-12 cells or control MLE-12 cells were incubated with GFP-Trap magnetic resin (ChromoTek) followed by washing and freezing of resin. Samples were processed and analyzed by MSBioworks (Ann Arbor, MI). Briefly, samples were boiled at 100°C for 15 minutes in 60 µL of 1.5X LDS buffer. Beads were removed and half of each submitted sample was processed by SDS-PAGE using a 10% Bis-Tris NuPAGE gel (Invitrogen) with the MES buffer system. The mobility region was excised into 10 equal sized segments and in-gel digestion was performed on each using a robot (ProGest, DigiLab) with the following protocol: Washed with 25mM ammonium bicarbonate followed by acetonitrile, reduced with 10mM dithiothreitol at 60°C followed by alkylation with 50 mM iodoacetamide at RT, digested with sequencing grade trypsin (Promega) at 37°C for 4 h, quenched with formic acid and the supernatant was analyzed directly without further processing.
Half of each digested sample was analyzed by nano LC-MS/MS with a Waters NanoAcquity HPLC system interfaced to a ThermoFisher Q Exactive. Data were filtered using at 1% protein and peptide FDR and requiring at least two unique peptides per protein.
For phospho-proteomics studies, TFEB-GFP MLE-12 cells were treated with vehicle or carfilzomib (1 µM for 4 h) prior to trypsinization and snap freezing. Cell lysate was sent to MSBioworks. Briefly, Samples were boiled at 100°C for 15 minutes in 60µL of 1.5X LDS buffer. Beads were removed and half of each submitted sample was processed by SDS-PAGE using a 4-12% Bis-Tris NuPAGE gel (Invitrogen) with the MOPS buffer system. The target band (TFEB+GFP) was excised and processed by in-gel digestion using a robot (ProGest, DigiLab) Data were filtered using at 1% protein and peptide FDR and requiring at least two unique peptides per protein.

Immunocytochemistry— BEAS-2B, MRC-5, or HCT-8 cells were seeded in 384-well glass-bottom plates (Cellvis, 5000 cells/well) prior to the indicated treatment. Viral inoculations were treated for 24-72 h prior to collection. Following treatment, cells were fixed (4% paraformaldehyde), permeabilized (0.5% Triton X-100), and the nucleus was counterstained with DAPI or Hoechst33342. Fluorescent signals were imaged using Image Express (Molecular Devices) for HCS experiments, or a Leica SP8 Confocal. Signal and nuclear-cytosol ratio were quantified using Cell Profiler [7].

Protein Half-Life Assay — To measure protein stability, the ribosomal inhibitor cycloheximide was used to halt protein synthesis, and TFEB protein stability was measured through immunoblotting. BEAS-2B cells, either WT or *DCAF7* Knockout (KO) or BEAS-2B cells transfected with V5-tagged WT or K232R TFEB plasmid were treated with cycloheximide (CHX) at 0.1mg/mL for the indicated times before cell lysis and processing for immunoblotting. TFEB protein level was detected via immunoblotting, and densitometry was performed using Fiji-ImageJ [8].

Cell Viability Assessment— Cell viability was tested using CellTiter-Glo 2.0 Cell Viability Assay (Promega). 20µl reagent was dispensed directly into each well of the 384-well tissue culture plates prior to luminescence signal acquisition by Cytation 5 plate reader.

GFP-TFEB nuclear localization protocol —TFEB-EGFP cells were plated @ 2,500 cells/well in 384-well glass bottom plates (Cellvis) in DMEM/F12, 3.2 g/L glucose (Gibco) containing 10% FBS, 25 mM HEPES, P/S, L-Glut. Cells were left at room temperature for 30-60 minutes before transferring to a standard tissue culture incubator. Six hours later, an equal volume of DMEM/F12, 1 g/L glucose (Gibco) containing 2% FBS, 25 mM HEPES, P/S, L-Glut was added, and the cells returned to the TC incubator. The next day cells were fixed by adding a half volume of pre-warmed 8% PFA/PBS containing 10 µg/L Hoechst 33342 (Invitrogen) and incubated for 1 hour. The cells were washed twice in PBS using a Biotek ELx405 automated washer and plates sealed. The cells were imaged using an ImageXpress Micro automated imager using a 10x objective and native camera resolution (Molecular Devices). The nuclear Hoechst and TFEB-EGFP signal were recorded. Using the Hoechst signal to define the nuclei, CellProfiler software was used to calculate the ratio of nuclear-to-cytoplasmic TFEB-EGFP localization for each cell. One field was used per well and the median nuclear-to-cytoplasmic TFEB-EGFP signal reported. For this calculation, the cytoplasmic signal was defined as the average GFP signal in a ring six pixels wide around the nucleus and the nuclear signal was defined as the average GFP signal in the nucleus.

Lysosomal number and activity— Cells were plated in 96-well glass bottom plates (Cellvis) in 100 µL total volume with test drugs and left at room temperature for 30-60 min to allow cells to attach prior to transfer to a tissue culture incubator. The next day, 70 µL media was aspirated and 15 µL media containing 10 µg/mL Hoechst 33342 (Invitrogen) and the following reagents added: For Lysotracker Red staining – CellTracker Green 1:1,000 and LysoTracker Red 1:2,500; for Magic Red activity - CellTracker Green 1:1,000 and Magic Red 1:125. The plate was returned to the incubator for 80 minutes. Next, the media was aspirated and 250 µL imaging solution added (150 mM NaCl, 5 mM KCl, 1 mM MgCl2, 2 mM CaCl2, 5% FBS, 1 g/L glucose, 20 mM HEPES, pH 7.4). The cells were then immediately imaged using an ImageXpress Micro XLS automated imager with 10x objective and the appropriate image filters (Molecular Devices). CellProfiler software was used to calculate the mean LysoTracker or Magic Red signal per cell, using the CellTracker signal as a mask to outline each cell. One field was used per well and the median signal for each well reported.

GFP-TFEB live-cell imaging — For live-cell imaging, TFEB-EGFP stable cells were plated in glass-bottom 96-well plates in normal growth media. The next day, the media was removed and replaced with modified imaging solution (140 mM NaCl, 2.5 mM KCl, 1 mM MgCl2, 1.8 mM CaCl2, 1 mM sodium phosphate monobasic, non-essential amino acids, MEM amino acid supplements, pen/strep, glutamax, 2 g/L sodium bicarbonate, 2% FBS) and supplemented with 0.05 µg/mL Hoechst 33342. Cells were incubated for 2 hours, the test drug was added, and imaging was started immediately using ImageXpress Micro XLS equipped with an environmental chamber. TFEB-EGFP nuclear-to-cytoplasmic signal was calculated as described above.

Lysosensor Yellow/Blue staining (pH)— Cells were plated in opaque black 96-well plates in normal growth media and allowed to attach. The media was then exchanged for DMEM with 1 g/L glucose, 2% FBS, Pen/strep and containing the test compounds. The next day, media was removed and 2 µM lysosensor yellow/blue was added to the imaging solution without FBS for 3 minutes at room temperature. The cells were then washed three times in the same buffer and incubated in the same buffer for 12 minutes before the signal was read using a BMG ClarioStar microplate reader (329-15 nm excitation/540-20 nm emission and 384-15 nm excitation/540-20 nm emission). For estimation of pH, wells on the same plate were incubated in potassium rich buffer containing the ionophores nigericin and monensin with pH 3.5 – 5.5 (10 mM NaCl, 135 mM KCl, 1g/L glucose, 1 mM CaCl2, 1 mM MgCl2, 10 µM nigericin, 2 µM monensin, 20 mM MES) to generate a standard curve. Lysosomal pH was determined from the ratio of light excited at 329 nm over 384 nm and interpolated from the standard curve using GraphPad Prism.

Construction of the lyso-Keima reporter (pLenti-CMV-Neo ss-Keima-LAMP1-mGFP) — To target the pH-sensitive fluorescent protein Keima into the lumen of lysosome, the sequence of Keima was inserted after the signal sequence (ss) of human LAMP1. Monomeric Keima was PCR amplified from the mito-Keima construct [9] using forward primer: 5’-GTGAGCGTGATCGCCAAGCAG-3’ and reverse primer: 5’-TTTCACCATAAACATGCCCAGCAGGGAGTG-3’. Human LAMP1 ss (1 – 29 aa) and LAMP1 (30 – 417 aa)-GFP sequence were PCR amplified from LAMP1-mGFP (Addgene #34831) using forward primer: 5’-CTCAAGCTTCGAATTACCATGGCGGCCCCCGGCAGCGC-3’ and reverse primer 5’- GGCGATCACGCTCACTGCTGCTGACGCACAATGC-3’, and forward primer: 5’-ATGTTTATGGTGAAAAATGGC-3’ and reverse primer 5’-GTCGACTGCAGAATTTCACTTGTACAGCTCGTC-3’, respectively. These PCR fragments were cloned into EcoRI-digested pENTR4 vector using In-Fusion assembly system (Takara). The ss-Keima-LAMP1-GFP sequence was then cloned into the pLenti-CMV-Neo vector using Gateway cloning system (ThermoFisher). Substitutions of three amino acids in the Keima amino acid sequence (Cys 102 to Val, Ser 105 to Thr, and Asn 106 to Gln) were found to facilitate the localization of the lyso-Keima reporter to lysosomes.

In-Cell ELISA Assays — BEAS-2B cells were plated in white 384-well plates (164610, Thermo) in complete DMEM at 7,500 cells per well and 293A cells were plated in poly-l-lysine-coated white 384-well plates (164610, Thermo) in complete DMEM at 15,000 cells per well. The plates were incubated at room temperature for 30–60 min before being transferred to a tissue culture incubator. The next day, test compounds and viral inoculates were prepared in glucose-free DMEM medium with 2% FBS, l-glutamine and pen/strep (P/S), HEPES. DMEM medium with 4.5 g/L glucose, 2% FBS, l-glutamine, pen/strep and HEPES was included for determination of non-specific background signal. The growth medium was removed from cells by flicking the plate upside down a few times and blotting the remaining liquid onto tissue paper. The cells were then washed in glucose-free DMEM medium with 2% FBS, l-glutamine, pen/strep and HEPES. This medium was removed as described above. Immediately thereafter, 30 µl of compound solution was added using an Agilent Bravo liquid handler and the cells were transferred to an incubator. The next day, cells were fixed by adding an equal volume of pre-warmed 8% paraformaldehyde (PFA)/PBS and allowed to sit for 30 min. The cells were washed twice in TBS using a Biotek 405TS automated washer. TBS was aspirated and cells permeabilized by adding 15 µl TBS/0.5% TX100, for 15 min. Cells were washed three times in TBS containing 0.05% Tween-20 (TBS-T). TBS-T was aspirated, and cells were blocked by adding 15 µl of TBS-T/1% BSA, for 15–60 min. The blocking solution was aspirated, and 15 µl of the primary antibody solution (OC43, EMDMillipore) in TBS-T/1% BSA) was added. The plates were sealed and incubated at 4 °C overnight or 2 h at room temperature. Next, the cells were washed four times in TBS-T, the TBS-T was aspirated and 15 µl of secondary antibody solution was added (αRabbit-HRP 1:20,000

(Invitrogen) in TBS-T/5% dry milk powder). Plates were sealed and incubated for 1 h at room temperature. The cells were then washed four times in TBS-T, the TBS-T was aspirated and 25 µl of (Enhanced chemiluminescent) ECL reagent was added. Twenty minutes later, luminescence was measured using a BMG ClarioStar microplate reader.

Kinase RNAi Library Screening— TFEB-EGFP BEAS-2B cells were seeded at a final density of 2000 cells per well in 384-well glass bottom plates before transfection with the MISSION siRNA Kinase panel (SigmaAldrich) using the XtremeGene siRNA transfection reagent (Roche). After 72 h, cells were fixed in 4% paraformaldehyde, counterstained with Hoechst33342, and fluorescence was detected using Cytation5 High Content Imager (BioTek). Nuclear to cytosolic ratio was calculated using the Gen5 software (BioTek).

In vitro protein binding assays— Protein binding assays were conducted as previously described [10]. Briefly, PAK2 or DCAF7 protein was immunoprecipitated from 1 mg of BEAS-2B cell lysate using 1:100 antibody dilution (Cell Signaling, Abcam). Protein was precipitated in IP buffer (50 mM Tris HCl pH 7.6, 150 mM NaCl, 0.25 % v/v Triton-X-100) for 4 hours at 4°C, then coupled to protein A/G agarose resin for an additional two hours. TFEB binding mutants were prepared through PCR cloning for *in vitro* synthesis using TnT expression kits and allowed to bind to immunoprecipitated PAK2 or DCAF7 overnight. Resin was washed and protein was eluted in 1x Laemmli buffer at 88°C for 5 minutes prior to immunoblotting analysis.

Molecular Docking Studies— The docking experiments were carried out using Discovery Studio 3.5 (BIOVIA). DCAF7 homology model was constructed using the Nurf55 WD domain crystal structure (2XYI.pdb)[11]. Using molecular docking analysis and score-ranking operations on the predicted DCAF7 WD repeat domain 3-D structure, we assessed potential ligands that might fit the domain cavity. These docking experiments were conducted using the LibDock program from Discovery Studio 3.5. A library containing 3 million small molecule compounds (ChemDiv, INC) was first used to screen potential ligands for the domain. The top score-ranking molecules were selected and further evaluated in vitro. We were able to identify and confirm the initial hit compound BC1753 *in vitro* and initiated a subsequent structural activity relationship (SAR) campaign.

Cellular thermal shift assay— Our procedure is based on previous literature [12]. HEK cells were transfected with DCAF7 plasmid overnight, prior to treatment with vehicle or BC18813 (3 µM for 1 h). Cells were collected and resuspended in 10 ml of PBS supplemented with EDTA-free protease inhibitor tablet. Cell solutions were aliquoted to 10 PCR microtubes evenly. Using the PCR thermocycler generating temperature gradient, each aliquot was incubated at a certain temperature between 40-58°C with 2°C interval for 3 min, then at room temperature for 3 min. Samples were immediately snap frozen in liquid nitrogen and 2 cycles of freeze-thaw followed. After vortexing briefly, samples were transferred to 1.7 ml microcentrifuge tubes for centrifuging at 20,000 g, 4°C for 20 min. Supernatants were carefully acquired and used for subsequent immunoblotting analysis.

Proximity Ligation Assay— The TFEB interaction with DCAF7 was measured by a Proximity Ligation Assay (PLA) using Duolink PLA kit (Sigma). Briefly, BEAS-2B cells were seeded in 384-well glass-bottom plates and treated with increasing concentrations of a DCAF7 inhibitor compound. Following treatment, cells were fixed in 4% PFA, permeabilized, blocked, and incubated with an anti-DCAF7 (Thermo) and anti-TFEB (Santa Cruz Biotechnology) antibody overnight. Cells were washed and incubated with PLA secondary antibody, ligase, polymerase, fluorescent dye, and counterstained with Hoechst33342. The PLA signal was visualized with Leica SP8 confocal, and the PLA signal per cell was calculated using Cell Profiler.

Histology and Immunohistochemistry— Following sacrifice, hamster lung samples were excised and immediately fixed in 10% formalin for 24 hours. Lung tissue was dehydrated with ethanol before embedding in paraffin to generate tissue blocks. Histological staining was conducted at the McGowen Transplant Institute at the University of Pittsburgh. Samples were sectioned using a microtome and processed for staining. Slides were stained for H&E or for immunohistochemistry of the SARS-CoV-2 nucleoprotein (Invitrogen, ma17403) at 1:100 dilution following the manufacturer's instructions. Stained lung slides were visualized using Cytation5 microscope (BioTek), and representative images were captured. Immunohistochemical quantification was conducted as previously described [13]. Briefly, image fields were chosen from each condition and RGB files were deconvoluted using ImageJ H-DAB algorithm [8]. Hematoxylin and DAB signal was thresholded; hematoxylin signal was used to estimate cell count, and DAB mean grey value was calculated. DAB mean grey value per cell number was calculated for each field, and ratios for each treatment normalized to mean vehicle were reported.

Synthetic Procedure for BC18813 and BC18630 —

N-(7-fluoro-1*H*-indol-5-yl)-2-pyridin-4-ylpyrido[2,3-*d*]pyrimidin-4-amine. hydrochloride (BC18813)

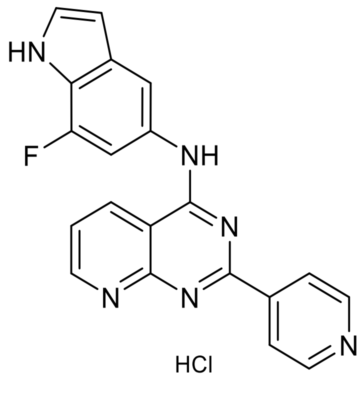

BC18813

Scheme 1. Synthesis of 7-fluoro-1*H*-indol-5-amine

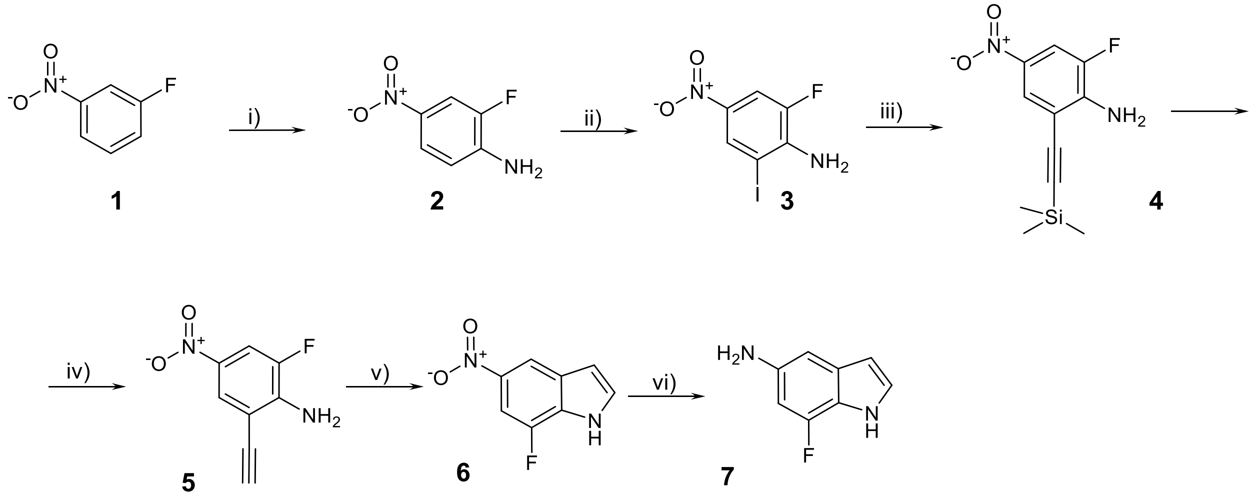

Reagents and conditions: (i) KOt-Bu, 1-amino-1,3,4-triazole, DMSO, ambient temperature; *ii)* NIS, AcOH, ambient temperature; *iii)* TMSA, CuI, $Pd(PPh_3)_4$, TEA, THF, ambient temperature; *iv)*

K_2CO_3, MeOH, ambient temperature; *v)* KOt-Bu, NMP, 50ºC; *vi)* H2, 10% Pd/C, EtOH, ambient temperature.

Step 1. Synthesis of compound 2

A solution of m-fluoro-nitrobenzene 1 (15.4 g, 0.11 mol) and 1-amino-1,3,4-triazole (9.2 g, 0.11 mol) in 100 mL of DMSO was added dropwise to a stirred solution of potassium tert-butoxide (12.3 g, 0.11 mol) in 200mL of DMSO. The reaction mixture was stirred overnight at ambient temperature, quenched with 600 mL of saturated aqueous solution of ammonium chloride, and extracted twice with ether. The combined organic layers were washed with water, dried over magnesium sulfate, and concentrated. The residue was subjected to silica column chromatography eluting with hexane/EtOAc to afford compound 2 (3.3 g, 19%).

Step 2. Synthesis of compound 3

N-Iodosuccinimide (5.24 g, 23.3 mmol) was added portion wise to a stirred solution of compound 2 (3.3 g, 21.1 mmol) in 30 mL of acetic acid. The resulting mixture was stirred overnight at ambient temperature and poured into ice cold water. The formed precipitate was filtered off, washed thoroughly with water, and dried to afford compound 3 (5.47 g, 92%).

Step 3. Synthesis of compound 4

Compound 3 (5.47 g, 19.4 mmol) and TEA (5.38 mL, 38.8 mmol) were dissolved in 30 mL of THF. The reaction vessel was evacuated and backfilled with argon several times. CuI (369 mg, 1.94 mmol) and $Pd(PPh_3)_4$ (1.12 g, 0.97 mmol) were added followed by addition of trimethylsilylacetylene (3.22 mL, 23.3 mmol). The reaction mixture was stirred overnight at ambient temperature, filtered through a of Celite pad, and evaporated to dryness under reduced pressure. The residue was subjected to silica column chromatography eluting with hexane/DCM to afford compound 4 (3.7 g, 76%).

Step 4. Synthesis of compound 5

Potassium carbonate (1.48 g, 10.7 mmol) was added to a stirred solution of Compound 4 (2.7 g, 10.7 mmol) in 50 mL of MeOH. The resulting mixture was stirred at ambient temperature until reaction completion (TLC monitoring), filtered through Celite pad, and evaporated to dryness under reduced pressure. Obtained crude compound 5 was used for the next step without further purification.

Step 5. Synthesis of compound 6

Potassium tert-butoxide (2.39 g, 21.3 mmol) was added to a stirred solution of the crude product 5 obtained at the previous step in 30 mL of NMP. The resulting mixture was stirred at 50ºC overnight, diluted with EtOAc, and washed three times with water. The combined organic layers were concentrated under reduced pressure, and the residue was subjected to silica column chromatography eluting with hexane/DCM to afford compound 6 (1.57 g, 81% over two steps).

Step 5. Synthesis of compound 7

A mixture of compound 6 (1.57 g, 8.7 mmol), 10% Pd on charcoal (0.15 g), and EtOH was stirred vigorously in in a hydrogen atmosphere until reaction completion (TLC monitoring), filtered through Celite pad, and evaporated to dryness under reduced pressure to afford (1.24 g, 95%) of the crude product 7 pure enough to be used at the next step.

Scheme 2. Synthesis of *N*-(7-fluoro-1*H*-indol-5-yl)-2-pyridin-4-ylpyrido[2,3-*d*]pyrimidin-4-amine. hydrochloride (BC18813)

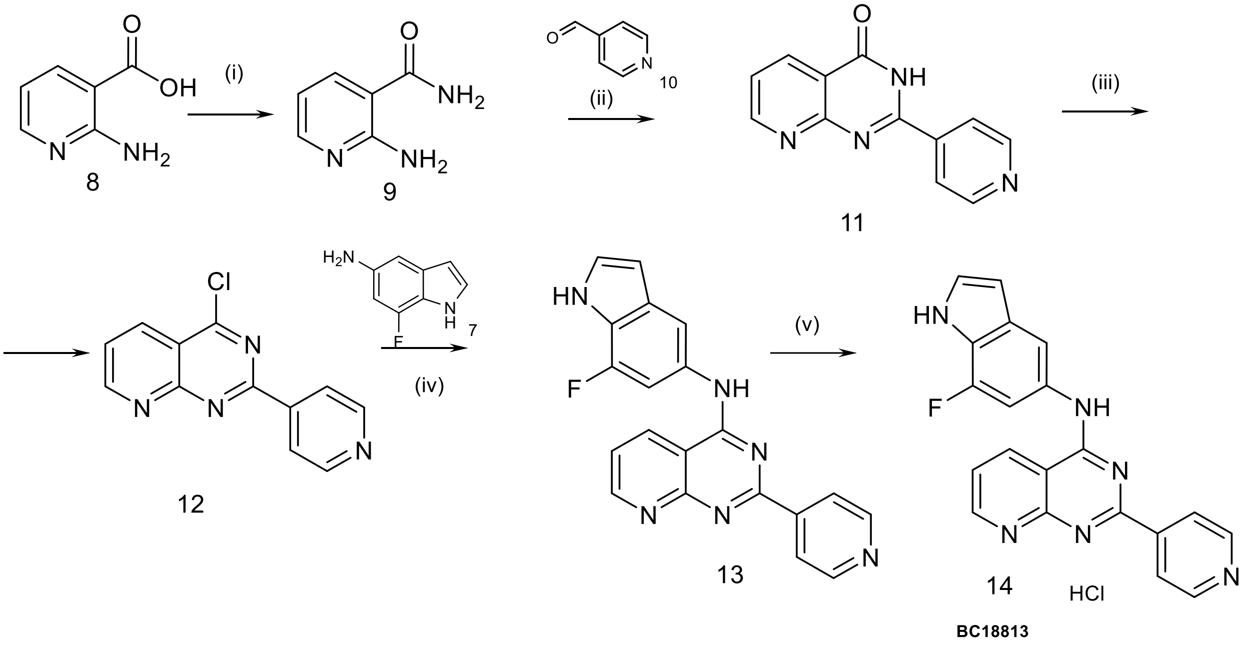

Reagents and conditions: (*i*) DMF, HOBT, EDC·HCl, NH_4Cl, Et_3N,12h,r.t; (*ii*) 4-pyridinecaboxaldehyde, DMSO, 100°C, overnight; (*iii*)$POCl_3$, PCl_5, reflux, 12 h, (*iv*) aniline A, K_2CO_3, DMF, r.t, overnight, (*v*) HCl

Step 1. Synthesis of compound 9.

A mixture of 2-aminonicotinic acid (11.04 g, 80.0 mmol), HOBT (10.8 g 80.0 mmol), EDC·HCl (11.47 g, 120.0 mmol), Et_3N (16.16 g, 160.0 mmol) and DMF (200 mL) was stirred overnight at ambient temperature and then concentrated under reduced pressure. The residue was partitioned between DCM (300 mL) and saturated aqueous solution of NaHCO3 (300 mL). The organic layer was separated, and the aqueous one extracted with DCM. The combined organic layers were dried over Na_2SO_4 and concentrated under reduced pressure. The residue was subjected to silica column chromatography eluting with EtOAc to afford compound 9 (9.27 g, 84%)

Step 2. Synthesis of compounds 11.

A mixture of anthranilamide 9 (4.5 g, 32.8 mmol), 4-pyridinecarboxaldehyde (3.86 g, 36.1 mmol), and DMSO (50 mL) was stirred at 100°C for 16 h, cooled to ambient temperature, and diluted with water (200 mL). Formed precipitate was collected by filtration, washed with water, and dried to afford compound 11 (4.3 g, 58%).

Step 3. Synthesis of compounds 12.

A mixture of the compound 11 (2.55 g, 11.37 mmol), PCl_5 (2.61 g, 12.5 mmol), and $POCl_3$ 52.35 g, 341.0 mmol) was stirred and heated under reflux for 6h, cooled to ambient temperature, and poured into ice. The obtained solution was neutralized with saturated aqueous solution of $NaHCO_3$ and extracted twice with EtOAc. Combined organic layers were washed with water, dried over Na_2SO_4 and concentrated under reduced pressure to afford compound 12 (2.60 g, 94%) pure enough to be used at the next step without further purification.

Step 4. Synthesis of compounds 13.

A mixture of 7-fluoro-1*H*-indol-5-amine 7 (1.5 g, 9.98 mmol), compound 12 (2.42 g, 9.98 mmol), K_2CO_3 (2.06 g, 14.98 mmol), and DMF (10 mL) was stirred at room temperature overnight and poured into water. Formed precipitate was filtered off, washed with water, dried, and subjected to HPLC purification to afford compound 13 (1.53g, 45%).

Step 5. Synthesis of compounds 14 (BC18813).

A 3M solution of HCl (1.5 mL) in ether was added to a stirred mixture of compound 13 (0.88 g; 2.25 mmol) and EtOH (20 mL). The mixture was vigorously stirred at ambient temperature for 15 min. The precipitate was filtered off, washed with acetone, and dried to afford target compound 14 (0.92 g, 99%).

^{1}H NMR (400.4 MHz, DMSO-d_6, δ): 9,11 - 9.18 (m, 2H); 8.98 (d, J = 5.9 Hz, 2H); 8.66 (d, J = 5.9 Hz, 2H); 7.75 – 7,84 (m, 2H); 7.42 – 7.49 (m, 2H), 6.62 (t, J = 2.9 Hz). MS (ESI): m/z 357.4 $[M+H]^+$.

N-1*H*-indol-5-yl-2-pyridin-4-ylquinazolin-4-amine, hydrochloride (*BC18630)*

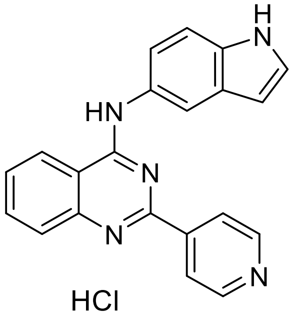

BC18630

Scheme 1. Synthesis of *N*-1*H*-indol-5-yl-2-pyridin-4-ylquinazolin-4-amine, hydrochloride (*BC18630)*

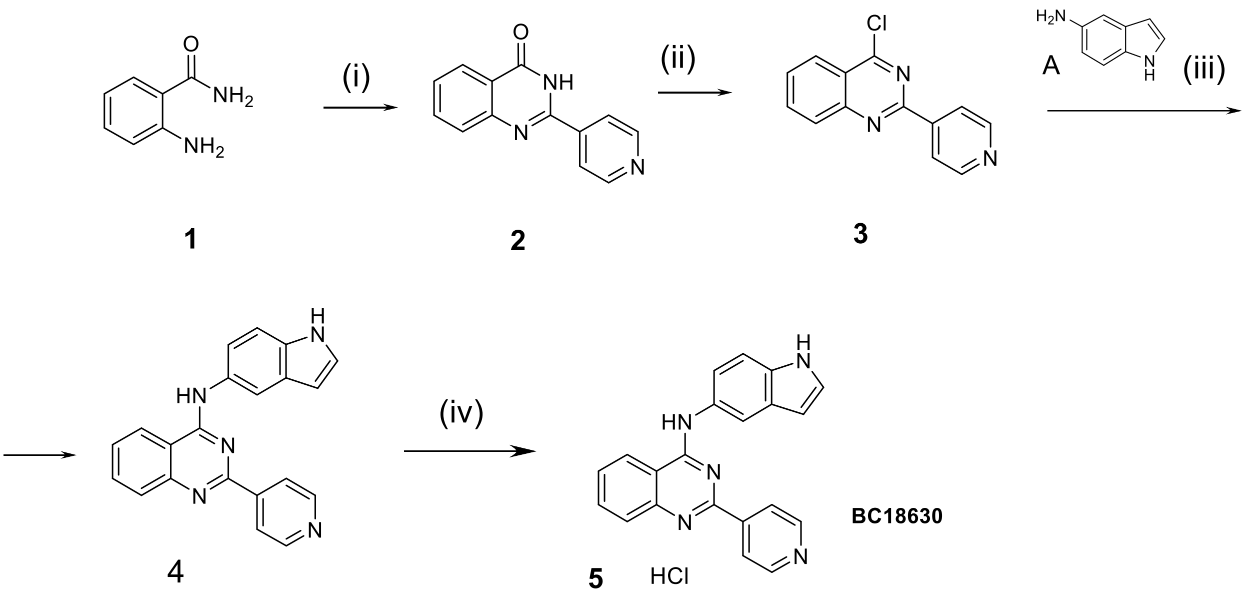

Reagents and conditions: (*i*) 4-pyridinecaboxaldehyde, DMSO, 100°C, overnight; (*ii*) $POCl_3$, PCl_5, reflux, 12 h; (*iii*), aniline A, K_2CO_3, DMF, r.t, overnight; (*iv*) HCl

Step 1. Synthesis of compound 2.

A mixture of antranilamide 1 (1.4 g, 10.35 mmol), 4-pyridinecarboxaldehyde (1.22g, 11.39 mmol), and DMSO (25 mL) was stirred at 100°C for 16 h, cooled to ambient temperature, and poured into ice cooled water (200 mL). Formed precipitate was collected by filtration, washed with water, and dried to provide compound 2 (1.93 g, 84 %).

Step 2._Synthesis of compounds 3.

The mixture of the compound 2 (1.57 g, 7.03 mmol), PCl_5 (1.61g, 7.73 mmol), and $POCl_3$ (32.4g, 210.9 mmol) was stirred and heated under reflux for 6h, cooled to ambient temperature, and poured into ice. The resulting mixture was neutralized with saturated aqueous solution of $NaHCO_3$ and extracted twice with EtOAc. Combined organic layers were washed with water, dried over Na_2SO_4 and concentrated under reduced pressure to afford compound 3 (1.96g, 83%) that was used for the next step without further purification.

Step 3._Synthesis of compounds 4.

A mixture of indol-5-amine A (1.07 g, 8.10 mmol), compound 3 (1.96 g, 8.10 mmol), K_2CO_3 (1.35 g, 9.72 mmol), and DMF (10 mL) was stirred at ambient temperature overnight and poured into ice cold water. The formed precipitate was filtered off, washed with water, dried, and subjected to HPLC purification to afford compound 4 (0.94 g, 40 %)

Step 5._Synthesis of compounds 5 (BC18630).

A 3M solution of HCl (1.7 mL) in ether was added to a stirred mixture of compound 4 (0.94 g; 2.28 mmol) and EtOH (20 mL). The mixture was vigorously stirred at ambient temperature for 15 min.

The precipitate was filtered off, washed with acetone, dried, and dissolved in min. amount of water. Obtained solution was lyophilized to afford target compound 5 (1 g, 96 %).

^{1}H NMR (400.4 MHz, DMSO-d_6, δ): 11.28 (br. s, 1H); 10.74 (br.s, 1H); 9.0 (d, J = 5.4 Hz, 2H); 8.78 (d, J = 7.8 Hz, 1H); 8.63 (d, J = 5.4 Hz, 2H); 8.10 (d, J = 8.3 Hz, 1H); 8.07 – 8.13 (m, 1H); 7.91 – 7.94 (br. m, 1H); 7.76 – 7.82 (m, 1H); 7.51 – 7.54 (br. m, 2H); 7.42 (t, J = 2.9 Hz). MS (ESI): m/z 338.2 $[M+H]^+$.

QUANTIFICATION AND STATISTICAL ANALYSIS

Statistical comparisons were performed in GraphPad Prism 9. All statistical details of experiments can be found in the figure legend. Unpaired two-tailed Student's *t*-test was used to compare two groups. Comparisons of more than two groups were tested with one-way or two-way ANOVA with post-hoc test of multiple comparisons. Significance was determined by $p < 0.05$ or greater and is indicated in figure legends.

DATA AND SOFTWARE AVAILABILITY

The published article includes all datasets generated or analyzed during this study.

Supplemental References:

1 Robertson, H., Hayes, J. D. & Sutherland, C. A partnership with the proteasome; the destructive nature of GSK3. *Biochemical pharmacology* **147**, 77-92, doi:10.1016/j.bcp.2017.10.016 (2018).

2 Kim, H. K. *et al.* Deep learning improves prediction of CRISPR–Cpf1 guide RNA activity. *Nature Biotechnology* **36**, 239-241, doi:10.1038/nbt.4061 (2018).

3 Sanjana, N. E., Shalem, O. & Zhang, F. Improved vectors and genome-wide libraries for CRISPR screening. *Nature methods* **11**, 783-784, doi:10.1038/nmeth.3047 (2014).

4 Koval, M. & Pagano, R. E. Sorting of an internalized plasma membrane lipid between recycling and degradative pathways in normal and Niemann-Pick, type A fibroblasts. *J Cell Biol* **111**, 429-442, doi:10.1083/jcb.111.2.429 (1990).

5 Sun, N. *et al.* Measuring In Vivo Mitophagy. *Mol Cell* **60**, 685-696, doi:10.1016/j.molcel.2015.10.009 (2015).

6 Lim, K. L. *et al.* Parkin mediates nonclassical, proteasomal-independent ubiquitination of synphilin-1: implications for Lewy body formation. *J Neurosci* **25**, 2002-2009, doi:10.1523/jneurosci.4474-04.2005 (2005).

7 McQuin, C. *et al.* CellProfiler 3.0: Next-generation image processing for biology. *PLOS Biology* **16**, e2005970, doi:10.1371/journal.pbio.2005970 (2018).

8 Schindelin, J. *et al.* Fiji: an open-source platform for biological-image analysis. *Nature Methods* **9**, 676-682, doi:10.1038/nmeth.2019 (2012).

9 Katayama, H., Kogure, T., Mizushima, N., Yoshimori, T. & Miyawaki, A. A sensitive and quantitative technique for detecting autophagic events based on lysosomal delivery. *Chem Biol* **18**, 1042-1052, doi:10.1016/j.chembiol.2011.05.013 (2011).

10 Lear, T. *et al.* Ubiquitin E3 ligase FIEL1 regulates fibrotic lung injury through SUMO-E3 ligase PIAS4. *The Journal of experimental medicine* **213**, 1029-1046, doi:10.1084/jem.20151229 (2016).

11 Nowak, A. J. *et al.* Chromatin-modifying complex component Nurf55/p55 associates with histones H3 and H4 and polycomb repressive complex 2 subunit Su(z)12 through partially overlapping binding sites. *The Journal of biological chemistry* **286**, 23388-23396, doi:10.1074/jbc.M110.207407 (2011).

12 Jafari, R. *et al.* The cellular thermal shift assay for evaluating drug target interactions in cells. *Nat Protoc* **9**, 2100-2122, doi:10.1038/nprot.2014.138 (2014).

13 Crowe, A. R. & Yue, W. Semi-quantitative Determination of Protein Expression using Immunohistochemistry Staining and Analysis: An Integrated Protocol. *Bio Protoc* **9**, doi:10.21769/BioProtoc.3465 (2019).

14 Roczniak-Ferguson, A. *et al.* The transcription factor TFEB links mTORC1 signaling to transcriptional control of lysosome homeostasis. *Sci Signal* **5**, ra42, doi:10.1126/scisignal.2002790 (2012).

15 Falcón-Pérez, J. M., Nazarian, R., Sabatti, C. & Dell'Angelica, E. C. Distribution and dynamics of Lamp1-containing endocytic organelles in fibroblasts deficient in BLOC-3. *J Cell Sci* **118**, 5243-5255, doi:10.1242/jcs.02633 (2005).

Supplemental Figure Legends:

Supplementary Figure 1. Viral infection reduces the nuclear pool of TFEB protein in a time-dependent manner. A-B. Immunoblot analysis of BEAS-2B lysate 72 hours after either OC43 (A) or 229E (B) infection. **C-D** Viral RNA quantification through real-time PCR of BEAS-2B following OC43 (C) or 229E (D) infection. OC43 and 229E mRNA signal is normalized to MOI=0.03 condition. Data represent ±SEM (n=3). **E-F.** Immunoblot analysis of fractionated lysate from BEAS-2B harvested at the indicated times following OC43 (0.03 MOI) (**E**) or 229E (0.03 MOI) (**F**) infection. *, $p<0.05$; ***, $p<0.001$; ****, $p<0.0001$; compared to control or as indicated by one-way ANOVA with Dunnett's multiple comparisons (C-D).

Supplementary Figure 2. DCAF7 is necessary and sufficient for TFEB ubiquitination with Lysine-232 as a candidate ubiquitin acceptor site. A. Coomassie protein staining of TFEB-EGFP pulldown prior to mass spec analysis for TFEB-interacting proteins. EGFP only and TFEB-EGFP are indicated. **B.** Transfection of increasing amounts of the E3 ligase DCAF7 dose-dependently reduced TFEB levels in BEAS-2B cells. **C**. DCAF7 increased TFEB ubiquitination in cell-based ubiquitination assay. Ubiquitin protein densitometry in TFEB pulldown was quantified; data represent mean ±SEM (n=3). **D.** Schematic of TFEB protein domains. Lysine-232 is a putative ubiquitination site. **E.** Immunoblot analysis of TFEB WT and K232R expressed in BEAS-2B cells and treated with cycloheximide (CHX) demonstrating longer half-life of the K232R mutant. **F.** Ubiquitination assay of WT TFEB and TFEB K232R mutant when co-expressed with DCAF7. **G.** *DCAF7* KO decreased TFEB poly-ubiquitination in cell-based ubiquitination assay. Data represent mean ±SEM (n=3). Carfilzomib, CFZ. **, $p<0.01$ compared to WT or control by unpaired two-sided t-test (C, G).

Supplementary Figure 3. DCAF7 deletion augments TFEB nuclear localization and prevents viral-induced TFEB degradation and infection A. Immunofluorescent microscopy of WT and *DCAF7* knockout (KO) BEAS-2B cells expressing EGFP-tagged TFEB. Scale bar indicates 25 µm. **B.** Immunoblot analysis of WT or *DCAF7* knockout (KO) BEAS-2B cells treated with the indicated initial MOI of OC43 virus and assessed 3 days post infection. **C.** Quantification of cell viability at day 3 post OC43 infection. Data from CellTiterGlo2.0 readings were normalized to untreated control and represent mean ±SEM (n=6). **D.** In-cell ELISA analysis of WT or *DCAF7* KO BEAS-2B cells treated with increasing MOI's of OC43 with subsequent detection of OC43 Nucleoprotein (NP) levels (72h). Data were normalized to WT MOI=0.03 NP levels and represent mean ±SEM (n=6). **E.** Immunoblot analysis of WT or *DCAF7* knockout (KO) BEAS-2B cells treated with the indicated MOI a dose course of H1N1 Influenza A virus and assessed 3 days post infection. NS, $p>0.05$; *, $p<0.05$; **, $p<0.01$; ***, $p<0.001$; ****, $p<0.0001$; compared to vehicle or control or as indicated by two-way ANOVA with Tukey's multiple comparisons (C-D).

Supplementary Figure 4. Reconstitution of DCAF7 restores viral susceptibility in *DCAF7* knockout cells. A. Immunoblot analysis of WT, *DCAF7* KO, or *DCAF7* KO with reconstituted WT DCAF7 BEAS-2B cells treated with the indicated MOI of OC43 (48h). **B.** Quantification of viability in WT, *DCAF7* KO, or reconstituted *DCAF7* KO cells following OC43 infection as measured with CellTiterGlo2.0. Data were normalized to control and represent mean ±SEM (n= 3). NS, $p>0.05$; *, $p<0.05$; ****, $p<0.0001$; as compared to vehicle or control or as indicated by two-way ANOVA with Tukey's multiple comparisons (B).

Supplementary Figure 5. TFEB interaction with PAK2 affects response to viral infection. A. Viability of cells treated as in Fig. 3E were measured with CellTiterGlo2.0. Data was normalized to control and represent mean ±SEM (n=6). **B.** Schematic of TFEB protein domains and deletion mapping to uncover the critical region for PAK2 binding. **C-D.** Binding assays between TFEB

deletion mutants and immunoprecipitated PAK2 protein. TFEB WT and mutant proteins were *in vitro* synthesized using transcription and translation (TnT) kit and were incubated with PAK2 immunoprecipitate overnight. Following binding, the pulldown was washed and eluted for immunoblot analysis. NS, p>0.05; *, p<0.05; **, p<0.01; ***, p<0.001; ****, p<0.0001; compared to vehicle or control or as indicated by two-way ANOVA with Tukey's multiple comparisons (A).

Supplementary Figure 6. The TFEB Phospho-Degron mutant is resistant to DCAF7 viral-induced degradation and infection. A. Schematic of TFEB phospho-proteomics experiment. Cells were treated with vehicle or the proteasomal inhibitor MG132 for 4 hr prior to LC/MS analysis of TFEB phosphorylation status to determine putative phospho-degron sites. Candidate sites are listed in Supplemental Table 1. **B.** TFEB phospho-mutant is resistant to DCAF7-induced poly-ubiquitination. **C.** Microscopic images of the cellular localization of TFEB-EGFP mutants with Ser-Ala mutations at the indicated amino acid residues. **D.** Immunoblot analysis of BEAS-2B cells transfected with WT and serine phospho-mutant TFEB. Transfected cells, where indicated, were infected with OC43 along with carfilzomib (CFZ) co-treatment. TFEB-HIS-pull down (PD) was used to measure phospho-serine levels for the indicated TFEB WT or mutant proteins. **E-F.** TFEB (E) and OC43 (F) protein densitometries were corrected to β-actin and normalized to WT control treatment or Empty MOI=0.03 treatment; data represent mean ±SEM (n=3). The phospho-mutant is resistant to degradation and reduces OC43 NP expression. NS, p>0.05; *, p<0.05; **, p<0.01; ***, p<0.001; ****, p<0.0001; compared to vehicle or control or as indicated by two-way ANOVA with Tukey's multiple comparisons (E-F).

Supplementary Figure 7. Development and validation of DCAF7 small molecular inhibitors. A. An *in silico* homology modeling of DCAF7 WD-repeat protein domain using the corresponding region in NURF55 (PDB: 2XYI). Modeling identified a potential cavity for small molecule binding. **B.** Immunoblot analysis of BEAS-2B cells treated with screening hit BC1753 (18h) for compounds effect on TFEB protein level. **C.** Immunofluorescent microscopy of TFEB-EGFP-BEAS-2B cells treated with a dose course of BC1753 (18h) to detect TFEB nuclear localization. **D.** BC1753 increases autophagic flux as measured by GFP-tagged LC3 punctae in the presence or absence bafilomycin A (BafA; 10 μM). **E.** BC1753 acidified lysosomal pH as detected by Lyso-Keima fluorescence. **F.** Hit-to-lead development of screening hit BC1753 to BC18630 and BC18813.

Supplementary Figure 8. Small molecule DCAF7 inhibitors prevents DCAF7-mediated TFEB degradation A. Quantification of a thermal shift assay of DCAF7 protein incubated with vehicle (DMSO) or BC18813 and heated prior to precipitation and immunoblot analysis. DCAF7 protein densitometry was normalized to lowest tested temperature (n=3). **B-C** TFEB cellular ubiquitination assay. TFEB ubiquitination is decreased with DCAF7 inhibitor BC18813 (**B**) or BC18630 (**C**) treatment. Carfilzomib, CFZ. **D.** Proximity Ligation Assay of TFEB and DCAF7. BC18630 decreased TFEB-DCAF7 association in the nucleus. Quantification of PLA signal per cell. Data represent mean and interquartile range in violin plot (n=11-31 cells). *, p<0.05; **, p<0.01; ***, p<0.001; ****, p<0.0001; indicative of different curve to fit each data set by F-test (A), or as compared to control as indicated by one-way ANOVA with Dunnett's multiple comparisons (D).

Supplementary Figure 9. Small molecule DCAF7 inhibitors increase TFEB nuclear localization A-D. Fluorescent imaging of endogenous TFEB nuclear localization in BEAS-2B cells treated with increasing concentrations of BC18813 (A-B) or BC18630 (C-D). TFEB nuclear-cytosolic ratio was quantified (B, D); data represent mean ±SEM (n=6). **E-H.** Fluorescent imaging of the kinetics of TFEB-EGFP nuclear localization with two different concentrations of BC18813 (E-F) or BC18630 (G-H). TFEB nuclear to cytosolic ratio was calculated and normalized to time 0 (F, H), data represent mean ±SEM, (n=6). *, p<0.05; **, p<0.01; ***, p<0.001; ****, p<0.0001;

as compared to vehicle/control or as indicated by one-way ANOVA with Dunnett's multiple comparisons (B, D).

Supplementary Figure 10. DCAF7 inhibitors dose-dependently increases TFEB nuclear localization in multiple cell lines. **A-D.** Fluorescent microscopy of endogenous TFEB protein localization in HCT-8 (**A-B**), and MRC-5 (**C-D**) cells treated with the indicated concentrations of BC18813 or BC18630 (18 h). **B, D.** Quantification of TFEB nuclear localization in HCT-8 (**B**) and MRC-5 (**D**) cells. Data represents TFEB nuclear to cytosolic ratio, mean ±SEM (n=6). *, p<0.05; **, p<0.01; **, p<0.001; ****, p<0.0001; as indicated by one-way ANOVA with Tukey's multiple comparisons (B, D).

Supplementary Figure 11. DCAF7 inhibitors stimulate expression of TFEB transcription targets. A-D. qPCR analysis of known lysosomal transcriptional targets of TFEB obtained from BEAS-2B cells treated for the indicated time with either BC18813 (A) or BC18630 (B) (both at 1 µM) or with increasing concentrations of BC18813 (C) or BC18630 (D) and harvested at 18 h. Data represent fold change in indicated target mRNA level relative to control treatment; mean ±SEM (n=3-6). *, p<0.05; **, p<0.01; ***, p<0.001; ****, p<0.0001; as compared to vehicle or control or as indicated by one-way ANOVA with Dunnett's multiple comparisons (A-D).

Supplementary Figure 12. DCAF7 small molecule inhibitors increase lysosomal number and activity. A-B. Immunoblot analysis WT or *DCAF7* knockout (KO) BEAS-2B cells treated with increasing concentrations of BC18813 (18h). Compound increases TFEB levels in WT cells only. (B) TFEB protein densitometry was corrected to β-actin and normalized to WT vehicle treatment; data represent mean ±SEM (n=3). **C-D.** Fluorescent micrograph of WT or *DCAF7* KO BEAS-2B cells treated with BC18630 and stained with LysoTracker, a measure of lysosomal number. (D) LysoTracker fluorescence was quantified, data represent median LysoTracker signal from each sample, mean ±SEM (n=3). **E-H.** Fluorescent micrograph of lysosomal activity in WT or *DCAF7* KO BEAS-2B cells treated with BC18813 (E-F) or BC18630 (G-H) as assessed by Magic Red intensity. Magic Red fluorescence quantified (F, H); data represent each well's median Magic Red signal intensity per cell, mean ±SEM (n=3). NS, p>0.05; *, p<0.05; **, p<0.01; ***, p<0.001; ****, p<0.0001; as indicated by one-way ANOVA with Tukey's multiple comparisons (D, F, H).

Supplementary Figure 13. DCAF7 inhibitors are specific for modulating TFEB levels and do not cause cell death. A. Off-targeting assay for DCAF7 inhibitors BC18630 and BC18813 in BEAS-2B cells. Known protein substrates of the various E3 ligases denoted in the figure. These E3 ligases were chosen since they exhibit the highest structurally homology to DCAF7. **B-C**. Calu-3 cells were treated with increasing concentrations of BC18813 (B) or BC18630 (C) for 96 hours prior to cell viability measurements with CellTiterGlo2.0. Data represent mean ±SEM (n=4).

Supplementary Figure 14. BC18630 shows favorable plasma pharmacokinetics profile an lysosomal efficacy *in vivo*. A-B. Pharmacokinetics of BC18630 in male rats administered at 5mg/kg intravenously (**A**) or 10 mg/kg through oral gavage (**B**). Data represent plasma concentrations at different time points from individual animals (n=3 per dose/route). **C.** Confocal imaging of mouse hepatic tissue following treatment with vehicle or BC18630 (i.p 50mg/kg for 4 days). 2 h after the last dose, mice were i.v. injected with Dextran cascade blue, a fluorescent dye that accumulates in the hepatic endo-lysosomal compartment. Liver samples were fixed, embedded, sectioned, and prepared for immunofluorescence detection of lysosomal abundance by LAMP1 staining.

Supplementary Figure 15. Small molecule DCAF7 inhibitors are protective against coronaviral infection *in vitro*. A. Immunoblot analysis of OC43 infected BEAS-2B cells treated

with BC18630 demonstrates maintenance of TFEB levels and reduced viral load with increasing compound concentration. **B-C.** In-cell ELISA analysis of OC43 infected HCT-8 cells treated with the indicated concentrations of BC18813 (B) and BC18630 (C). OC43 infectivity was detected by OC43 Nucleoprotein (NP) expression. Data represent mean value (n=4). **D-E.** HCT-8 cells treated as in panel (B-C) but assessed by quantifying the percent of OC43-positive, HCT-8 cells through high-content fluorescent imaging of OC43 Nucleoprotein. Data represent mean value (n=3-4). **F.** Phase micrograph of cytopathic effects from *alpha* coronavirus 229E infection in MRC-5 cells in the absence (-) or presence (+) of BC18813 (72h). **G-H.** Quantification of cell number by CellTiterGlo2.0 following infection with *alpha* coronavirus 229E and treatment with BC18813 (G) or BC18630 (H). Data represent mean (n= 3-4). **I-J.** qPCR analysis of 229E viral RNA following infection and treatment with increasing concentrations of BC18813 (I) or BC18630 (J). Data represent mean ±SEM (n= 3). **L.** Immunoblotting of MCDK cells treated with a dose course of BC18813 and infected with H1N1 for 72 hours. Compounds maintain TFEB protein levels and reduce viral protein in a concentration-dependent fashion. NP, nucleoprotein. NS, $p>0.05$; ****, $p<0.0001$; as indicated by one-way ANOVA with Tukey's multiple comparisons (I-J).

Supplementary Figure 16. BC18813 decreases SARS-CoV-2 infection of Calu-3 cells. A-C. Cell-based SARS-CoV-2 infection assay. Briefly, Calu-3 human lung cells were pre-treated with the indicated concentrations of BC18813 for 4 h prior to incubation with SARS-CoV-2 virus (USA-WA-1/2020, MOI 0.01). After a 75 min inoculation, media was replaced with fresh media containing the indicated concentrations of compound. Supernatant samples were taken after 48 hours for viral RNA detection (**A**). Data represent mean ±SEM (n=6), and IC50 values were determined by sigmoidal nonlinear regression. Cells were fixed and stained for SARS-CoV-2 nucleoprotein (NP) for fluorescent microscopy and quantification of viral signal (**B-C**). Data represent mean ±SEM (n=6), and IC50 values were determined by sigmoidal nonlinear regression. *, $p<0.05$; ***, $p<0.001$; ****, $p<0.0001$; as indicated by one-way ANOVA with Dunnett's multiple comparisons (A, C).

Supplementary Figure 17: Schematic of proposed model. Viral infection activates PAK2 kinase that phosphorylates TFEB to create phospho-degron motif. Phosphorylated TFEB is then recognized as a substrate for the CRL4-DCAF7 E3 ligase complex, in which the DCAF7 subunit facilitates the ubiquitination and proteasomal degradation of TFEB. Chemical inhibition of DCAF7 by small molecules preserves TFEB protein, and increases expression of key TFEB transcription targets, thus enhancing lysosomal biogenesis and activity. Inhibiting pathogen-induced TFEB protein degradation maintains endo-lysosomal activity including lysosomal acidification thereby limiting viral infectivity.

Supplemental Figure Data

Figure S1

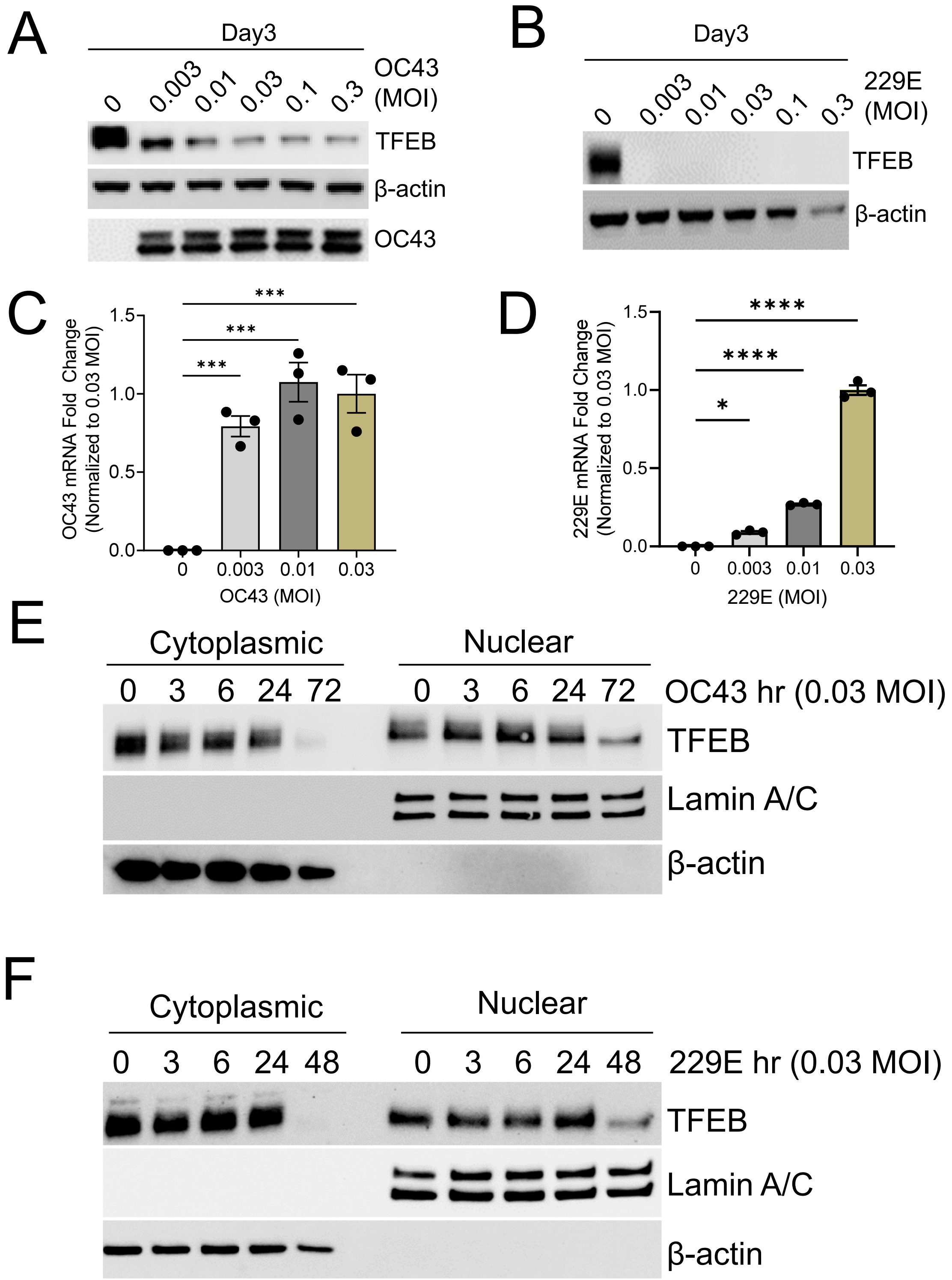

A
Day3
0
0.003
0.01
0.03
0.1
0.3
OC43 (MOI)
TFEB
β-actin
OC43
B
Day3
0
0.003
0.01
0.03
0.1
0.3
229E (MOI)
TFEB
β-actin
C
OC43 mRNA Fold Change (Normalized to 0.03 MOI)
1.5
1.0
0.5
0.0
***
***
***
0
0.003
0.01
0.03
OC43 (MOI)
D
229E mRNA Fold Change (Normalized to 0.03 MOI)
1.5
1.0
0.5
0.0
****
****
*
0
0.003
0.01
0.03
229E (MOI)
E
Cytoplasmic
Nuclear
0 3 6 24 72
0 3 6 24 72
OC43 hr (0.03 MOI)
TFEB
Lamin A/C
β-actin
F
Cytoplasmic
Nuclear
0 3 6 24 48
0 3 6 24 48
229E hr (0.03 MOI)
TFEB
Lamin A/C
β-actin

Figure S2

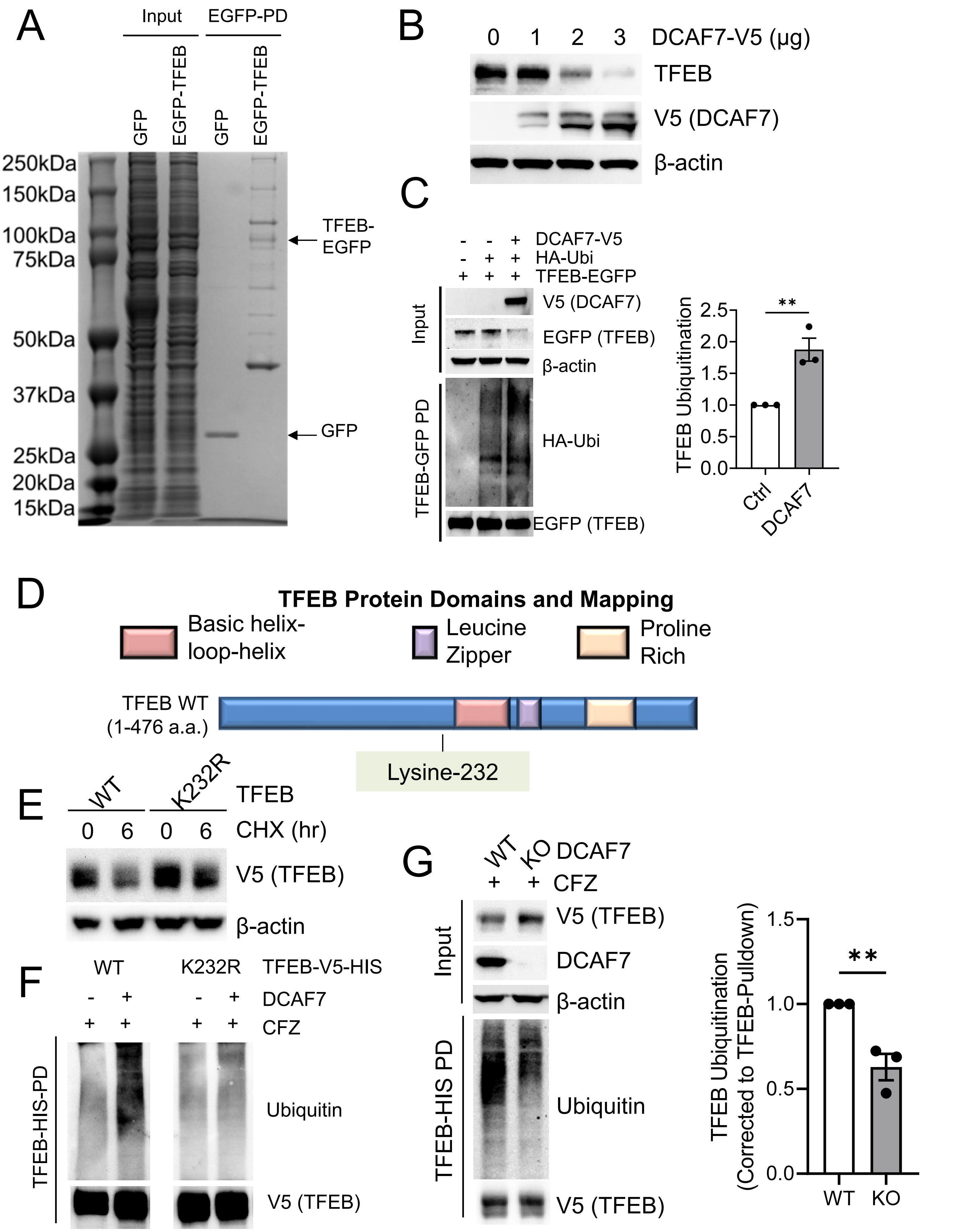

A
Input
EGFP-PD
GFP
EGFP-TFEB
GFP
EGFP-TFEB
250kDa
150kDa
100kDa
75kDa
50kDa
37kDa
25kDa
20kDa
15kDa
TFEB-EGFP
GFP
B
0 1 2 3 DCAF7-V5 (μg)
TFEB
V5 (DCAF7)
β-actin
C
DCAF7-V5
HA-Ubi
TFEB-EGFP
Input
V5 (DCAF7)
EGFP (TFEB)
β-actin
TFEB-GFP PD
HA-Ubi
EGFP (TFEB)
TFEB Ubiquitination
**
Ctrl
DCAF7
D
TFEB Protein Domains and Mapping
Basic helix-loop-helix
Leucine Zipper
Proline Rich
TFEB WT (1-476 a.a.)
Lysine-232
E
WT
K232R
TFEB
0 6 0 6 CHX (hr)
V5 (TFEB)
β-actin
F
WT K232R TFEB-V5-HIS
DCAF7
CFZ
TFEB-HIS-PD
Ubiquitin
V5 (TFEB)
G
WT KO DCAF7
CFZ
Input
V5 (TFEB)
DCAF7
β-actin
TFEB-HIS PD
Ubiquitin
V5 (TFEB)
TFEB Ubiquitination (Corrected to TFEB-Pulldown)
**
WT
KO

Figure S3

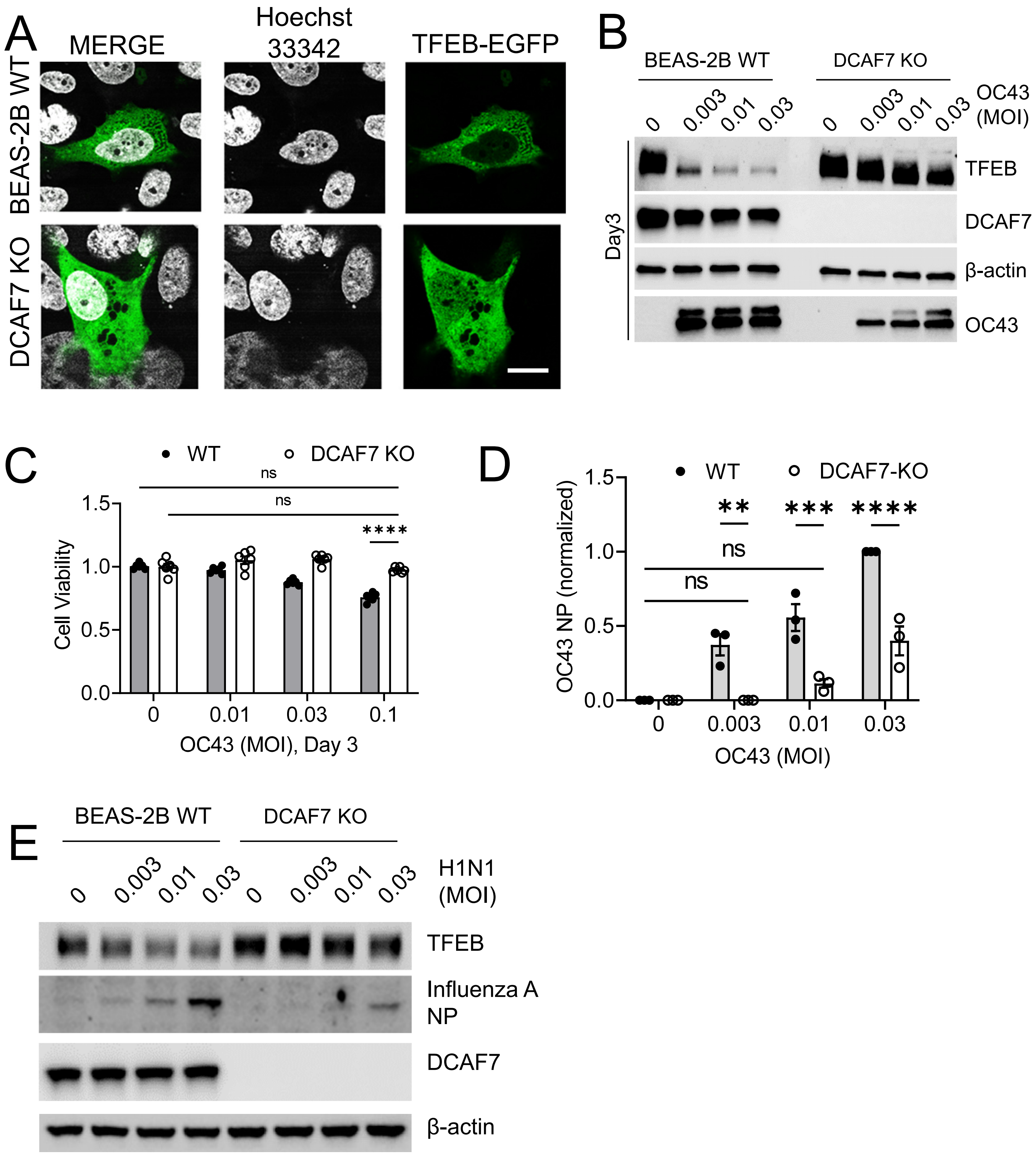

A
MERGE
Hoechst 33342
TFEB-EGFP
BEAS-2B WT
DCAF7 KO
B
BEAS-2B WT
DCAF7 KO
0
0.003
0.01
0.03
OC43 (MOI)
Day3
TFEB
DCAF7
β-actin
OC43
C
WT
DCAF7 KO
ns
****
Cell Viability
1.5
1.0
0.5
0.0
0.1
OC43 (MOI), Day 3
D
WT
DCAF7-KO
**
***
****
OC43 NP (normalized)
OC43 (MOI)
E
H1N1 (MOI)
Influenza A NP

Figure S4

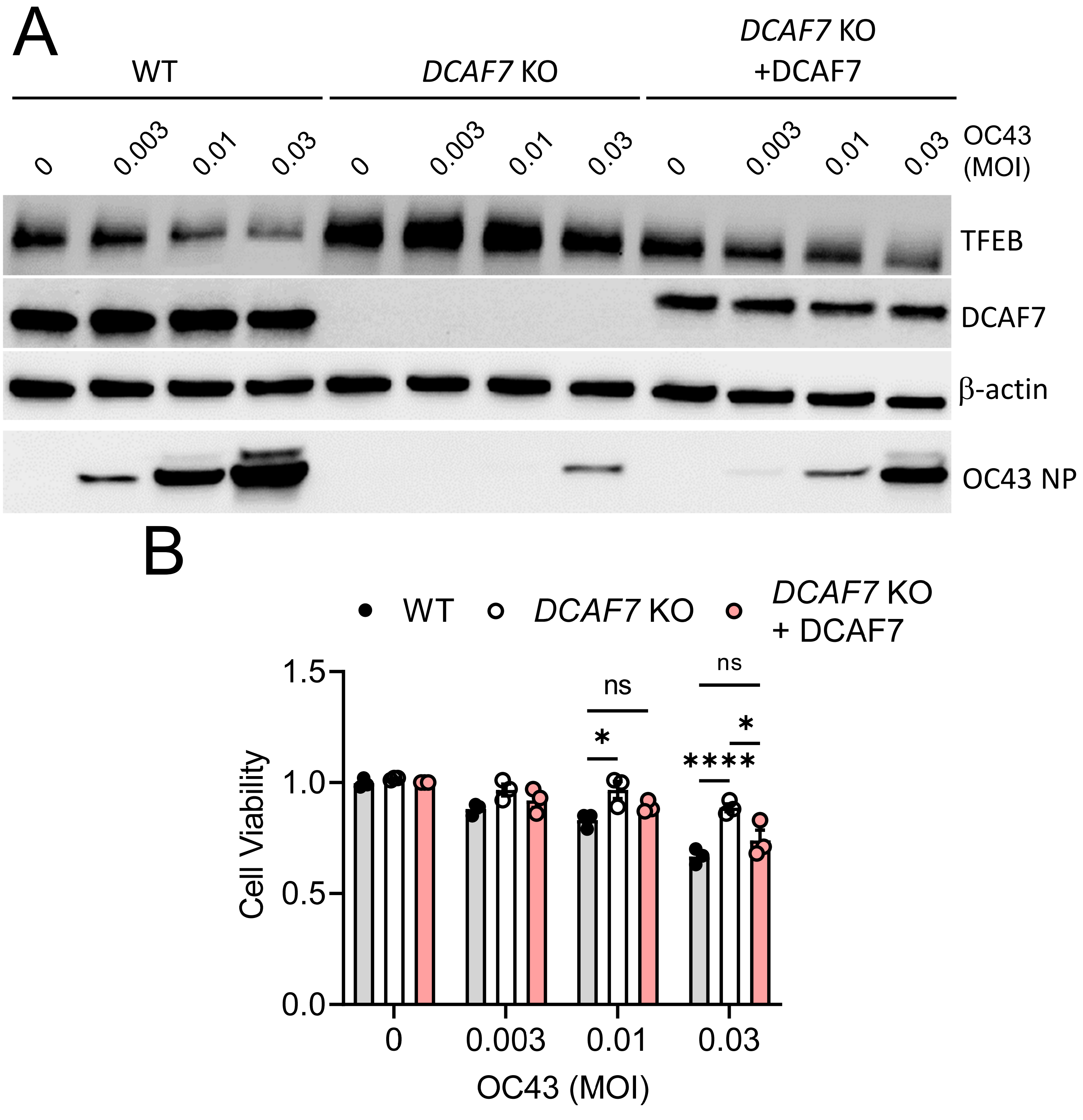
A
WT
DCAF7 KO
DCAF7 KO
+DCAF7
0
0.003
0.01
0.03
OC43
(MOI)
TFEB
DCAF7
β-actin
OC43 NP
B
WT
DCAF7 KO
DCAF7 KO
+ DCAF7
Cell Viability
1.5
1.0
0.5
0.0
ns
*
****
OC43 (MOI)

Figure S5

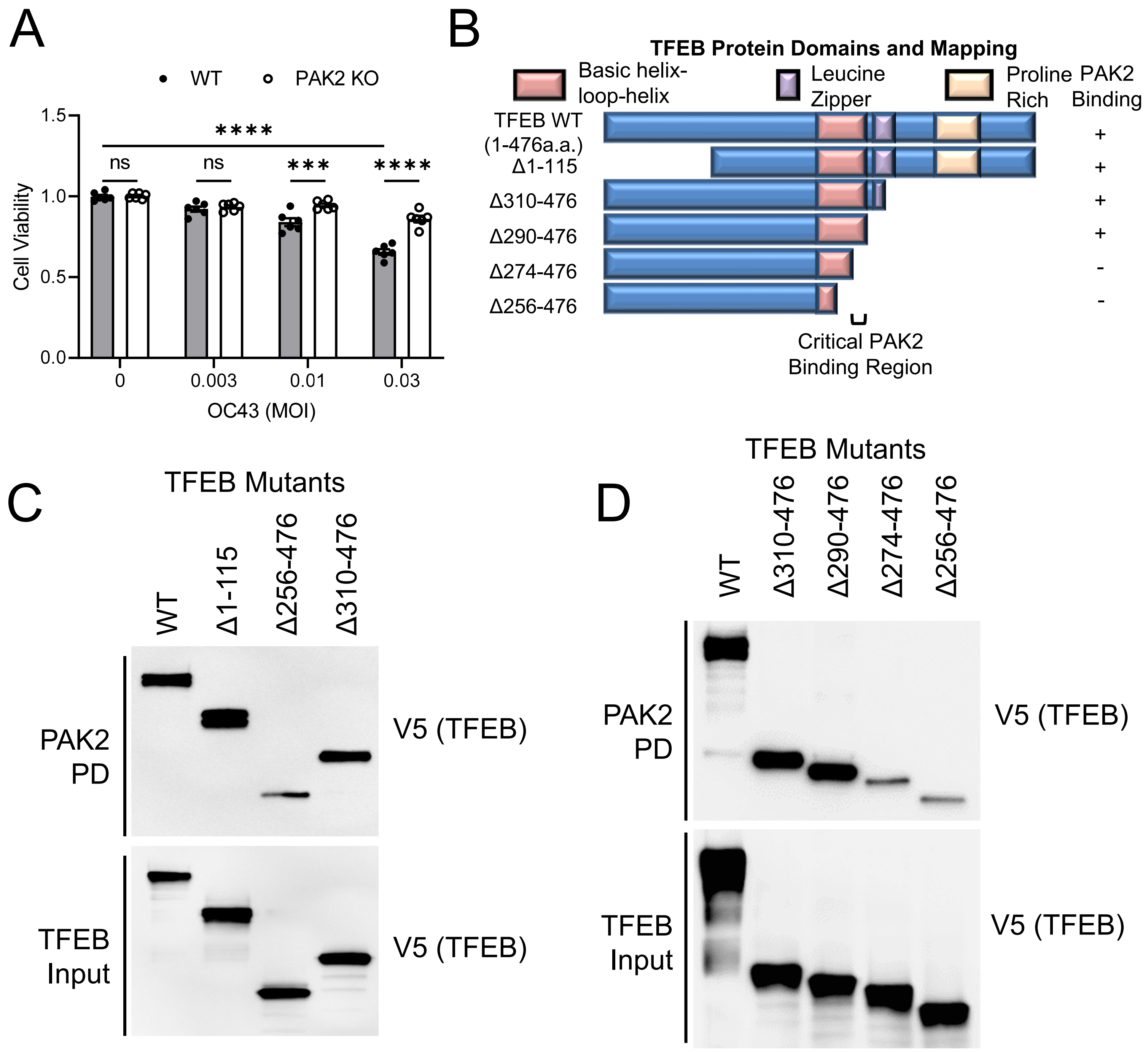
A
WT
PAK2 KO
Cell Viability
1.5
1.0
0.5
0.0
****
ns
ns
***
****
0
0.003
0.01
0.03
OC43 (MOI)
B
TFEB Protein Domains and Mapping
Basic helix-loop-helix
Leucine Zipper
Proline Rich
PAK2 Binding
TFEB WT (1-476a.a.)
Δ1-115
Δ310-476
Δ290-476
Δ274-476
Δ256-476
+
+
+
+
-
-
Critical PAK2 Binding Region
C
TFEB Mutants
WT
Δ1-115
Δ256-476
Δ310-476
PAK2 PD
TFEB Input
V5 (TFEB)
V5 (TFEB)
D
TFEB Mutants
WT
Δ310-476
Δ290-476
Δ274-476
Δ256-476
PAK2 PD
TFEB Input
V5 (TFEB)
V5 (TFEB)

Figure S6

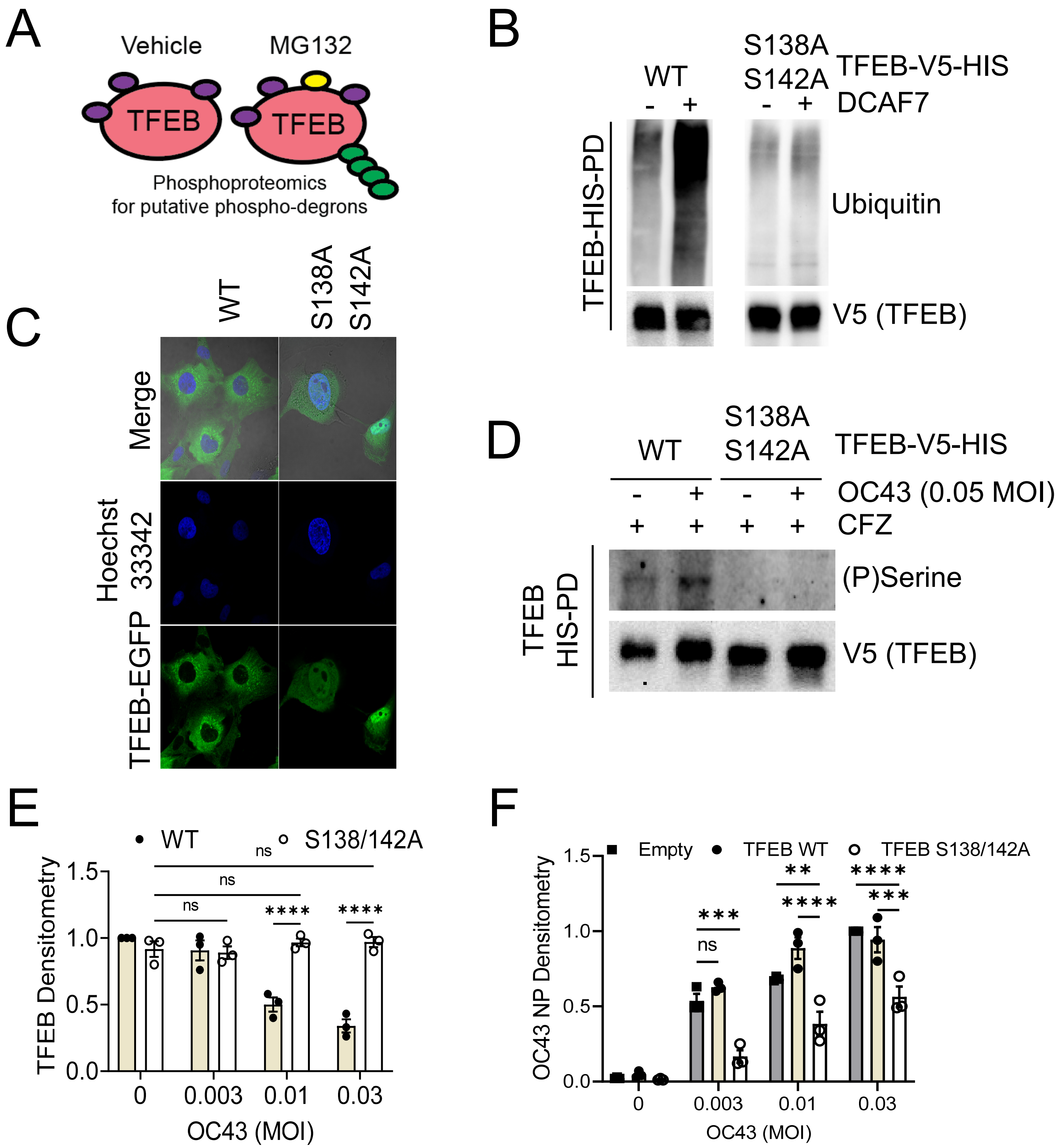

A
Vehicle
MG132
TFEB
TFEB
Phosphoproteomics
for putative phospho-degrons
B
WT
S138A
S142A
TFEB-V5-HIS
DCAF7
TFEB-HIS-PD
Ubiquitin
V5 (TFEB)
C
WT
S138A
S142A
Merge
Hoechst 33342
TFEB-EGFP
D
WT
S138A
S142A
TFEB-V5-HIS
OC43 (0.05 MOI)
CFZ
TFEB HIS-PD
(P)Serine
V5 (TFEB)
E
WT
S138/142A
TFEB Densitometry
OC43 (MOI)
ns
****
F
Empty
TFEB WT
TFEB S138/142A
OC43 NP Densitometry
OC43 (MOI)
ns
**
***
****

Figure S7

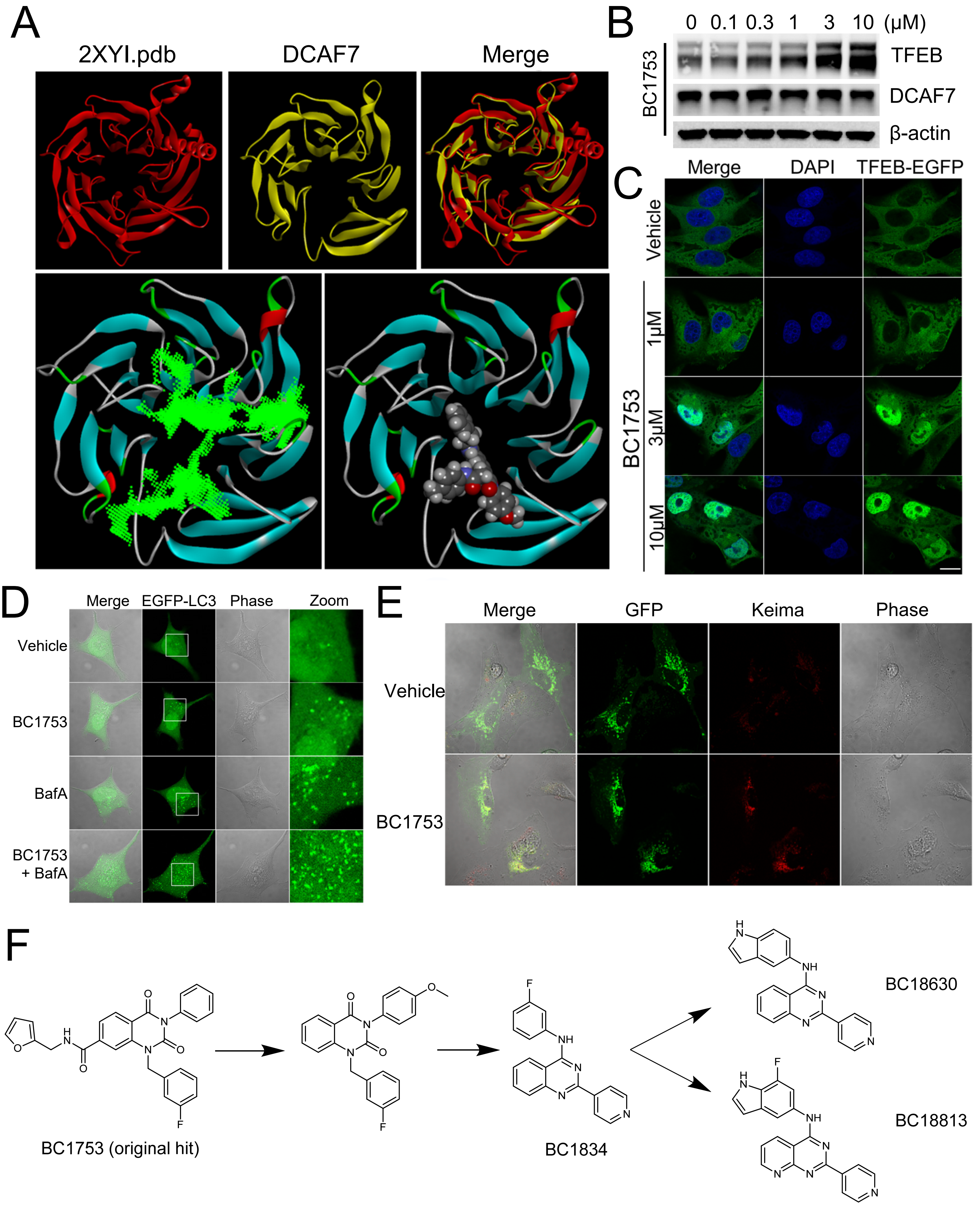

A
2XYI.pdb
DCAF7
Merge
B
0 0.1 0.3 1 3 10 (μM)
BC1753
TFEB
DCAF7
β-actin
C
Merge
DAPI
TFEB-EGFP
Vehicle
BC1753
1μM
3μM
10μM
D
Merge
EGFP-LC3
Phase
Zoom
Vehicle
BC1753
BafA
BC1753
+ BafA
E
Merge
GFP
Keima
Phase
Vehicle
BC1753
F
BC1753 (original hit)
BC1834
BC18630
BC18813

Figure S8

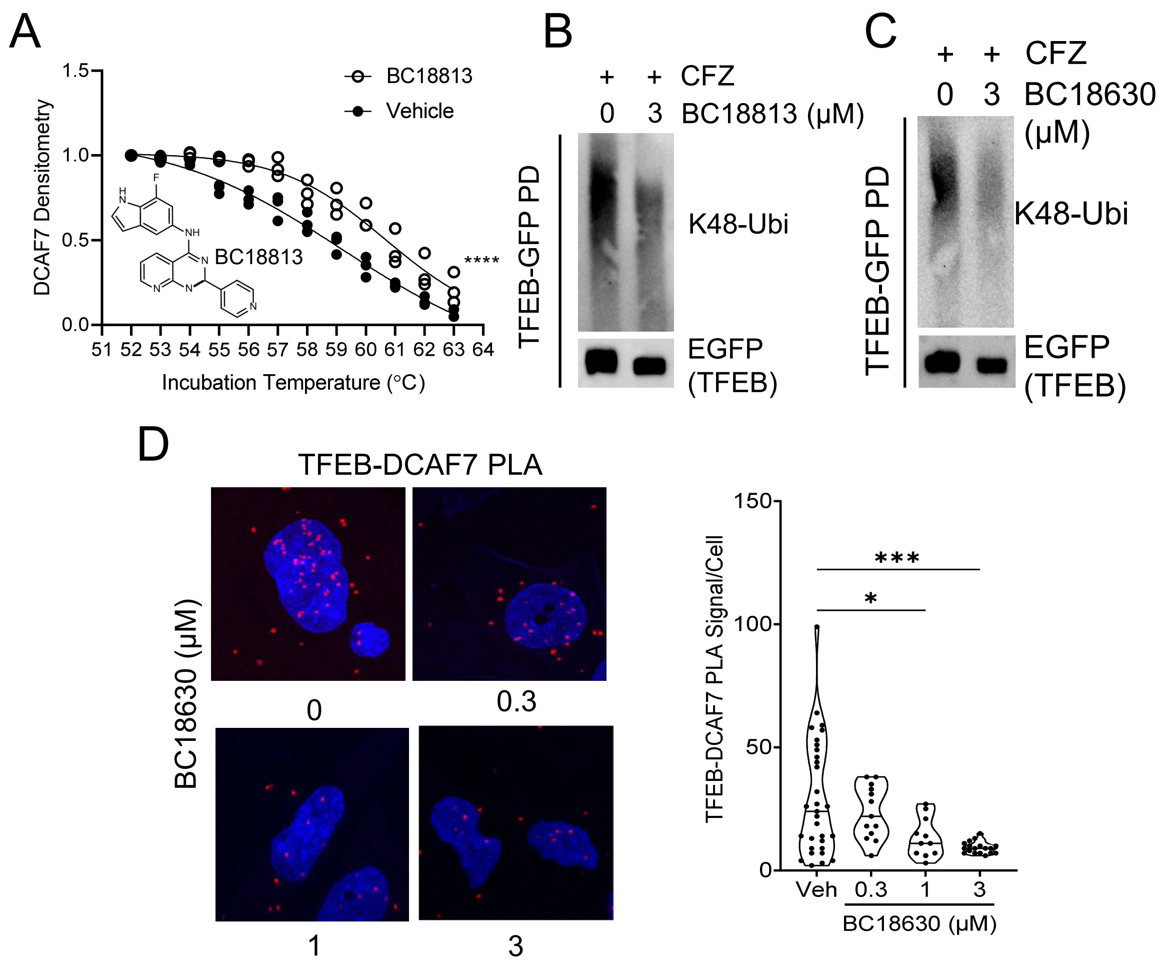

A
BC18813
Vehicle
DCAF7 Densitometry
1.5
1.0
0.5
0.0
BC18813
****
51 52 53 54 55 56 57 58 59 60 61 62 63 64
Incubation Temperature (°C)
B
+ + CFZ
0 3 BC18813 (μM)
TFEB-GFP PD
K48-Ubi
EGFP (TFEB)
C
+ + CFZ
0 3 BC18630 (μM)
TFEB-GFP PD
K48-Ubi
EGFP (TFEB)
D
TFEB-DCAF7 PLA
BC18630 (μM)
0
0.3
1
3
TFEB-DCAF7 PLA Signal/Cell
150
100
50
0
***
*
Veh 0.3 1 3
BC18630 (μM)

Figure S9

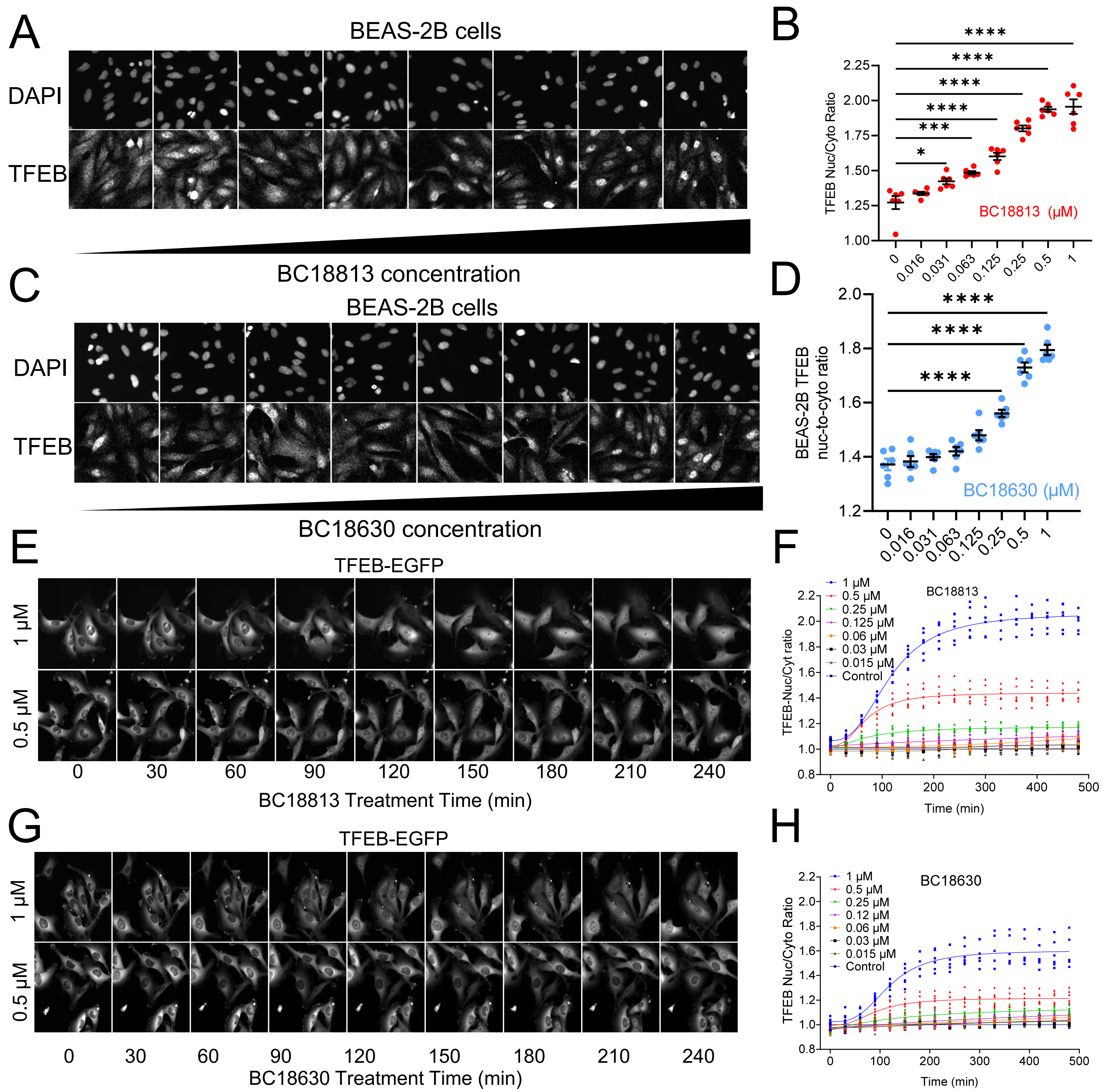

A
BEAS-2B cells
DAPI
TFEB
BC18813 concentration
B
TFEB Nuc/Cyto Ratio
BC18813 (μM)
C
BEAS-2B cells
DAPI
TFEB
BC18630 concentration
D
BEAS-2B TFEB nuc-to-cyto ratio
BC18630 (μM)
E
TFEB-EGFP
1 μM
0.5 μM
0
30
60
90
120
150
180
210
240
BC18813 Treatment Time (min)
F
BC18813
TFEB-Nuc/Cyt ratio
Time (min)
1 μM
0.5 μM
0.25 μM
0.125 μM
0.06 μM
0.03 μM
0.015 μM
Control
G
TFEB-EGFP
1 μM
0.5 μM
BC18630 Treatment Time (min)
H
BC18630
TFEB Nuc/Cyto Ratio
Time (min)
0.12 μM

Figure S10

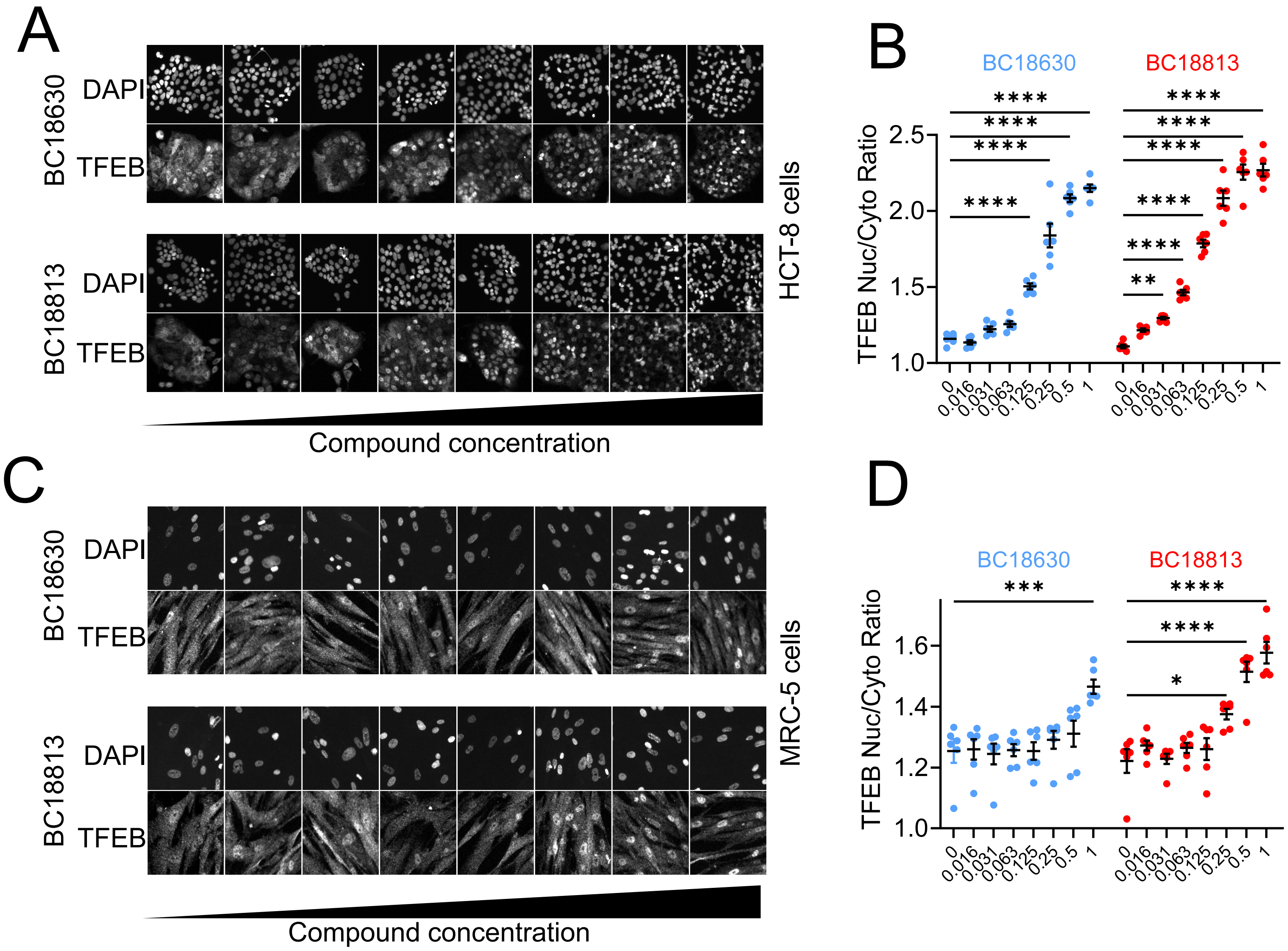
A
BC18630
DAPI
TFEB
BC18813
DAPI
TFEB
HCT-8 cells
Compound concentration
B
BC18630
BC18813
TFEB Nuc/Cyto Ratio
2.5
2.0
1.5
1.0
****
**
0
0.016
0.031
0.063
0.125
0.25
0.5
1
C
BC18630
DAPI
TFEB
BC18813
DAPI
TFEB
MRC-5 cells
Compound concentration
D
BC18630
BC18813
TFEB Nuc/Cyto Ratio
1.6
1.4
1.2
1.0
***
****
*

Figure S11

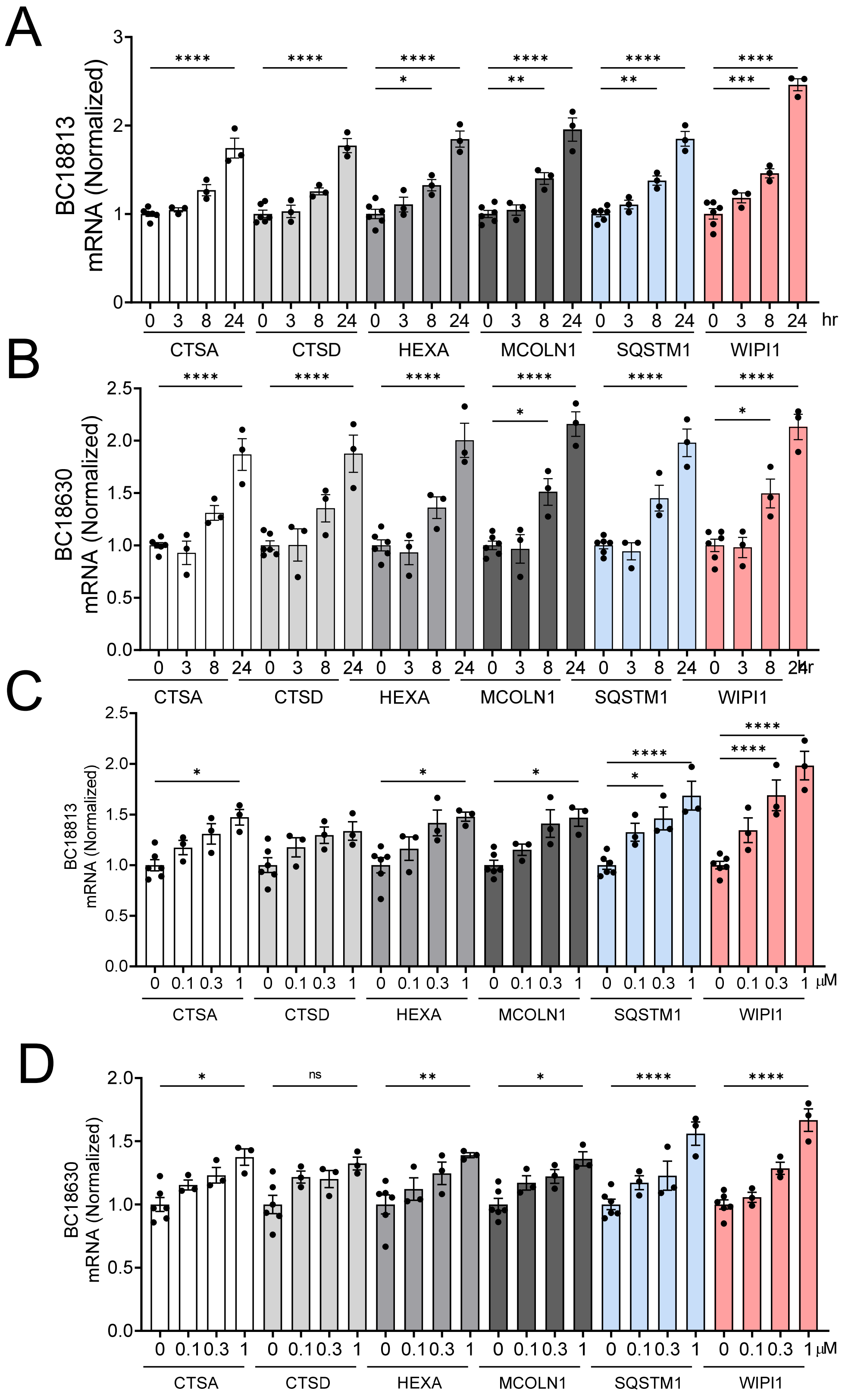
A
BC18813 mRNA (Normalized)
0 3 8 24 hr
CTSA CTSD HEXA MCOLN1 SQSTM1 WIPI1
B
BC18630 mRNA (Normalized)
0 3 8 24 hr
CTSA CTSD HEXA MCOLN1 SQSTM1 WIPI1
C
BC18813 mRNA (Normalized)
0 0.1 0.3 1 μM
CTSA CTSD HEXA MCOLN1 SQSTM1 WIPI1
D
BC18630 mRNA (Normalized)
0 0.1 0.3 1 μM
CTSA CTSD HEXA MCOLN1 SQSTM1 WIPI1

Figure S12

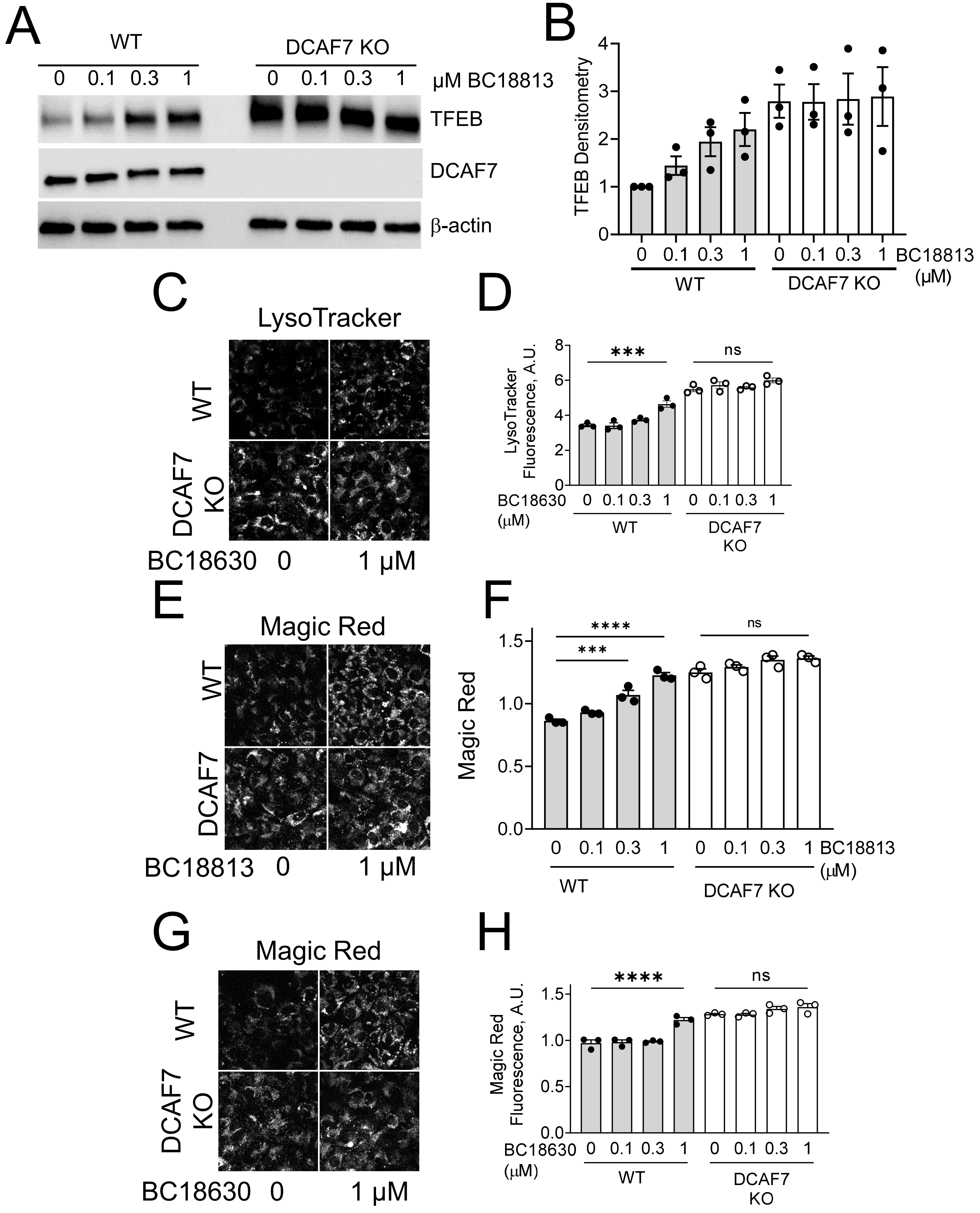
A
WT
DCAF7 KO
0 0.1 0.3 1 0 0.1 0.3 1 μM BC18813
TFEB
DCAF7
β-actin
B
TFEB Densitometry
0 1 2 3 4
0 0.1 0.3 1 0 0.1 0.3 1 BC18813 (μM)
WT
DCAF7 KO
C
LysoTracker
WT
DCAF7 KO
BC18630 0 1 μM
D
LysoTracker Fluorescence, A.U.
0 2 4 6 8
***
ns
BC18630 (μM) 0 0.1 0.3 1 0 0.1 0.3 1
WT
DCAF7 KO
E
Magic Red
WT
DCAF7
BC18813 0 1 μM
F
Magic Red
0.0 0.5 1.0 1.5
****
***
ns
0 0.1 0.3 1 0 0.1 0.3 1 BC18813 (μM)
WT
DCAF7 KO
G
Magic Red
WT
DCAF7 KO
BC18630 0 1 μM
H
Magic Red Fluorescence, A.U.
0.0 0.5 1.0 1.5
****
ns
BC18630 (μM) 0 0.1 0.3 1 0 0.1 0.3 1
WT
DCAF7 KO

Figure S13

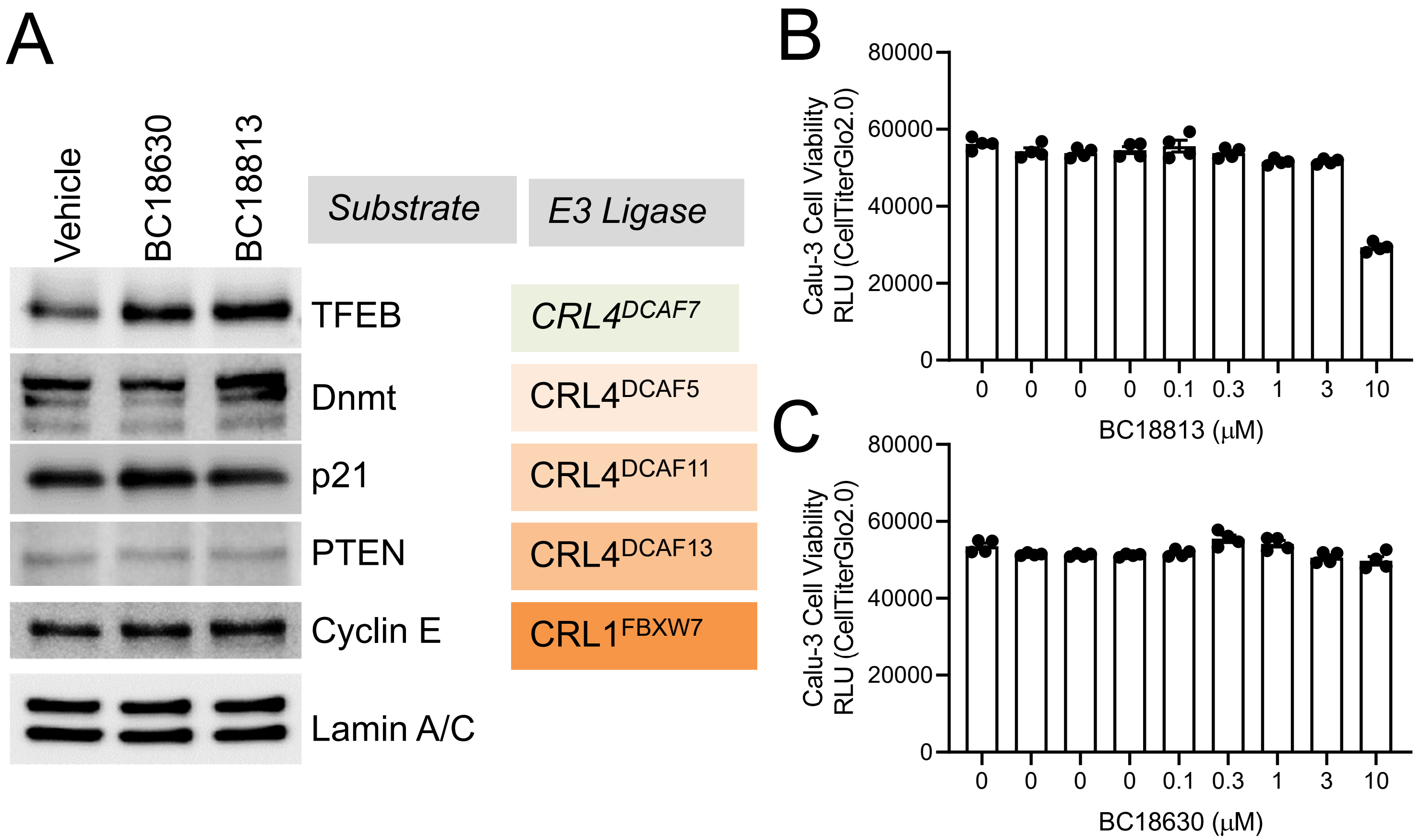
A
Vehicle
BC18630
BC18813
Substrate
E3 Ligase
TFEB
CRL4DCAF7
Dnmt
CRL4DCAF5
p21
CRL4DCAF11
PTEN
CRL4DCAF13
Cyclin E
CRL1FBXW7
Lamin A/C
B
Calu-3 Cell Viability
RLU (CellTiterGlo2.0)
80000
60000
40000
20000
0
0 0 0 0 0.1 0.3 1 3 10
BC18813 (µM)
C
Calu-3 Cell Viability
RLU (CellTiterGlo2.0)
80000
60000
40000
20000
0
0 0 0 0 0.1 0.3 1 3 10
BC18630 (µM)

Figure S14

BC18630 ADME Studies (PK, IV and PO)

A

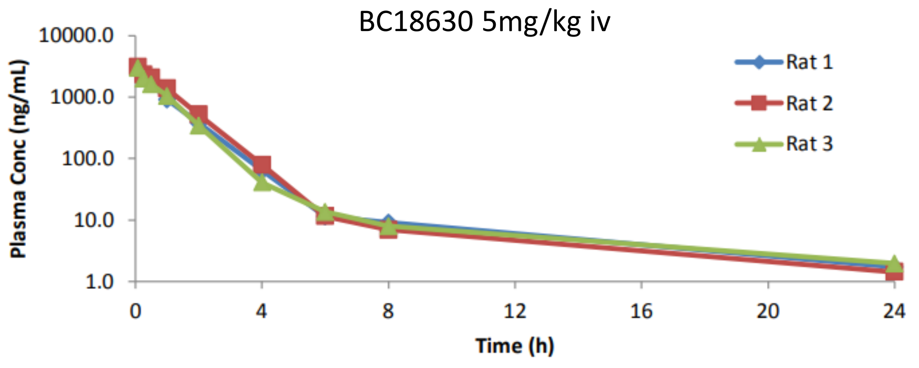

B

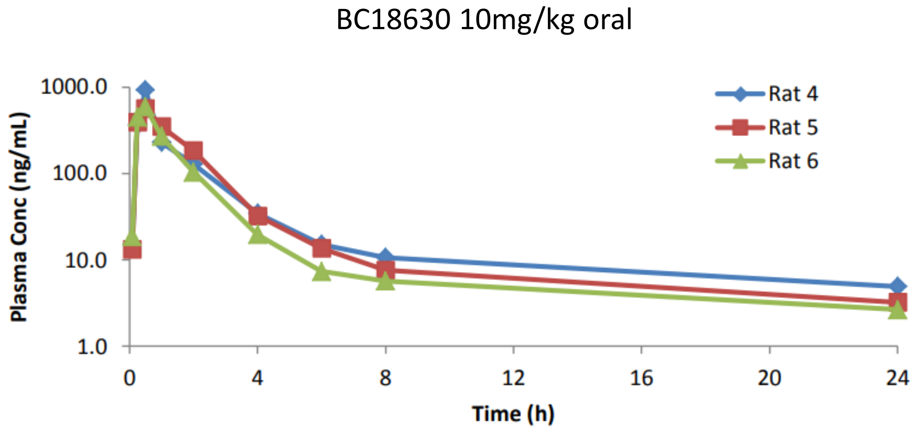

C

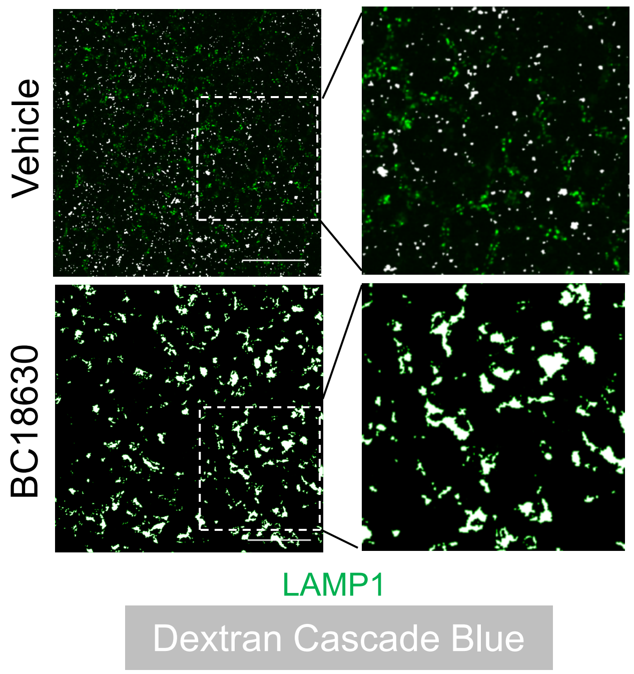

Figure S15

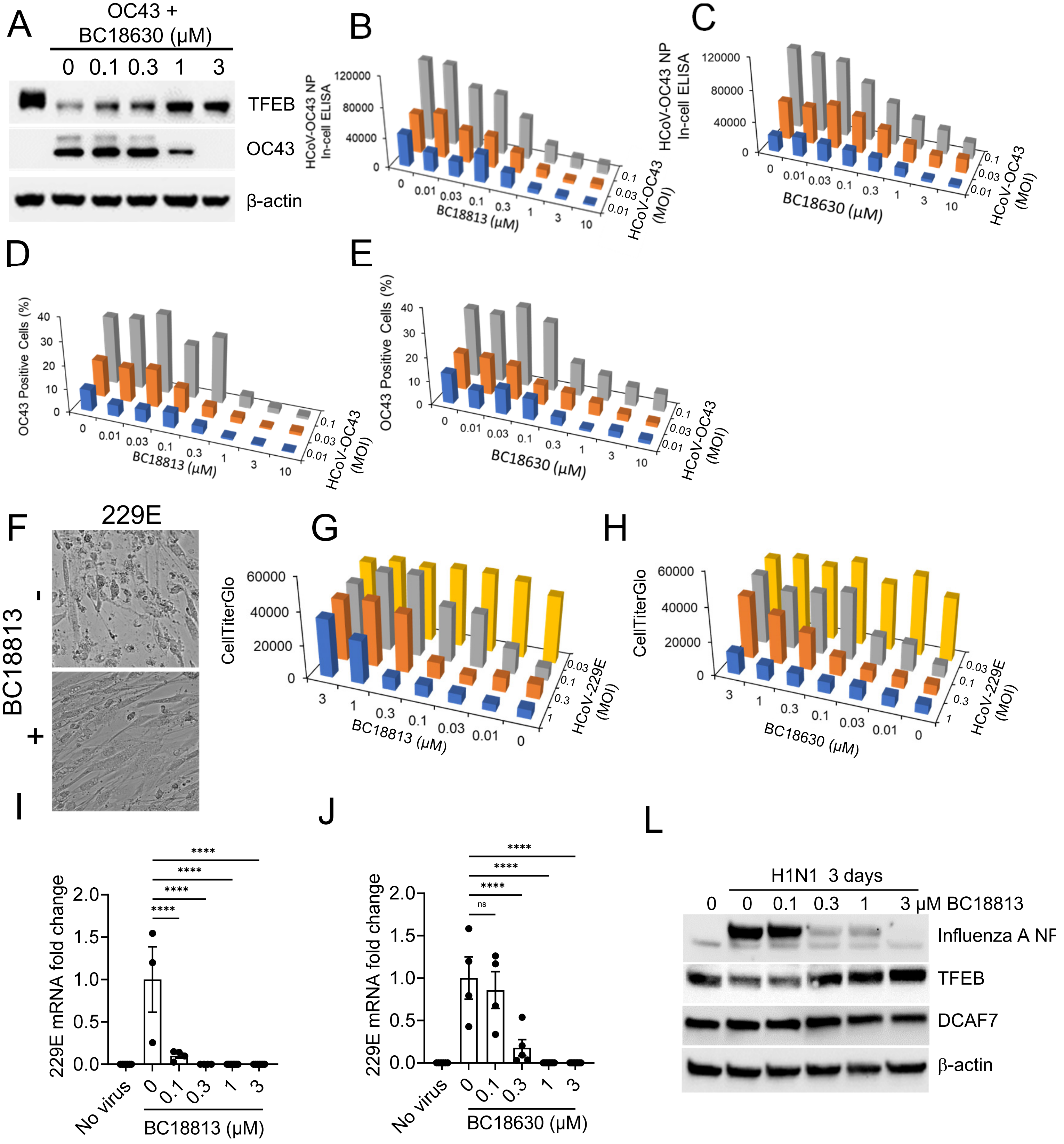

A
OC43 +
BC18630 (µM)
0 0.1 0.3 1 3
TFEB
OC43
β-actin
B
HCoV-OC43 NP In-cell ELISA
BC18813 (µM)
HCoV-OC43 (MOI)
C
HCoV-OC43 NP In-cell ELISA
BC18630 (µM)
HCoV-OC43 (MOI)
D
OC43 Positive Cells (%)
BC18813 (µM)
HCoV-OC43 (MOI)
E
OC43 Positive Cells (%)
BC18630 (µM)
HCoV-OC43 (MOI)
F
229E
BC18813
-
+
G
CellTiterGlo
BC18813 (µM)
HCoV-229E (MOI)
H
CellTiterGlo
BC18630 (µM)
HCoV-229E (MOI)
I
229E mRNA fold change
No virus
BC18813 (µM)
J
229E mRNA fold change
No virus
BC18630 (µM)
ns
L
H1N1 3 days
0 0 0.1 0.3 1 3 µM BC18813
Influenza A NP
TFEB
DCAF7
β-actin

Figure S16

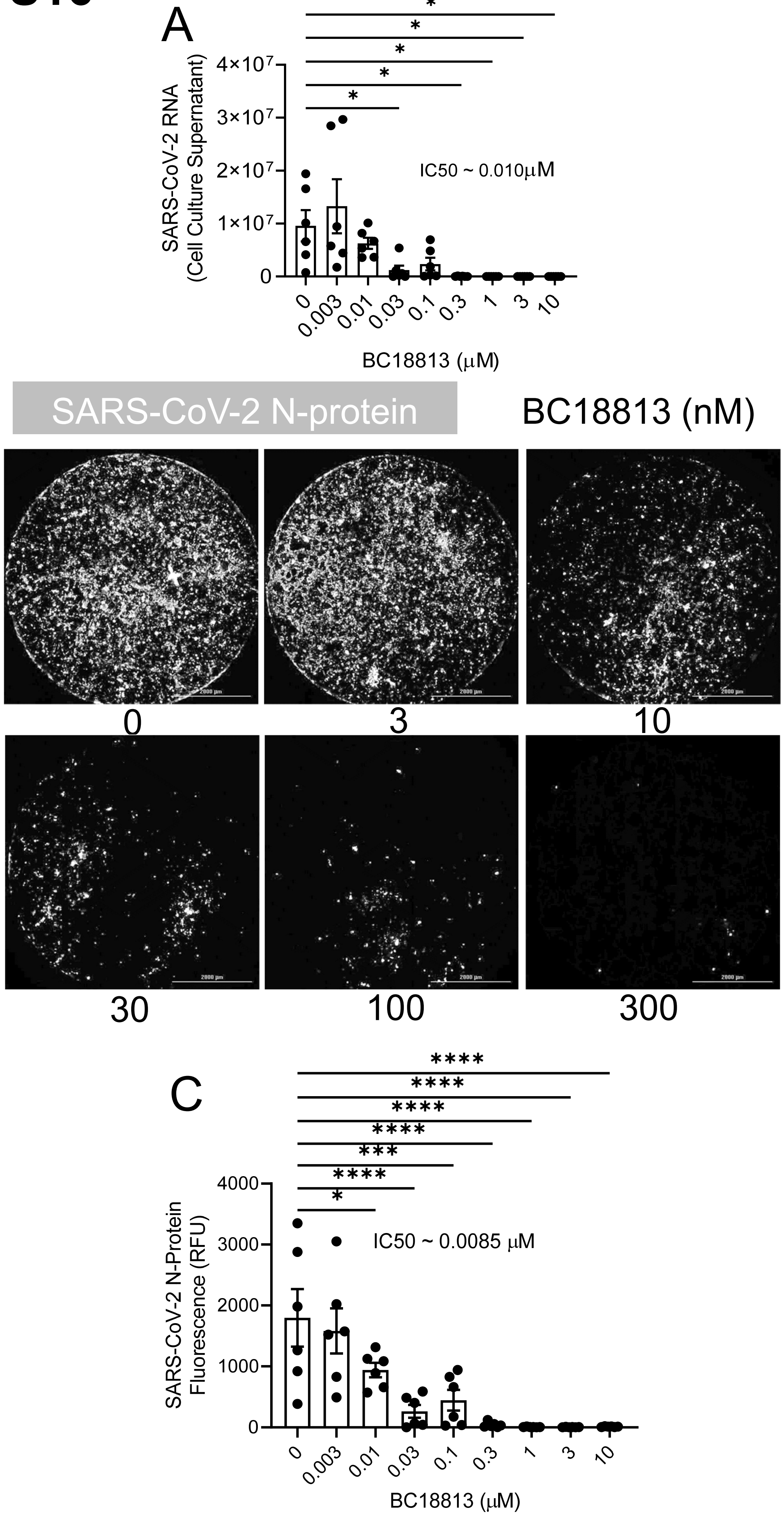

A
SARS-CoV-2 RNA (Cell Culture Supernatant)
4×10^7
3×10^7
2×10^7
1×10^7
0
IC50 ~ 0.010μM
0 0.003 0.01 0.03 0.1 0.3 1 3 10
BC18813 (μM)
B
SARS-CoV-2 N-protein
BC18813 (nM)
0
3
10
30
100
300
C
SARS-CoV-2 N-Protein Fluorescence (RFU)
4000
3000
2000
1000
0
IC50 ~ 0.0085 μM
0 0.003 0.01 0.03 0.1 0.3 1 3 10
BC18813 (μM)

Figure S17

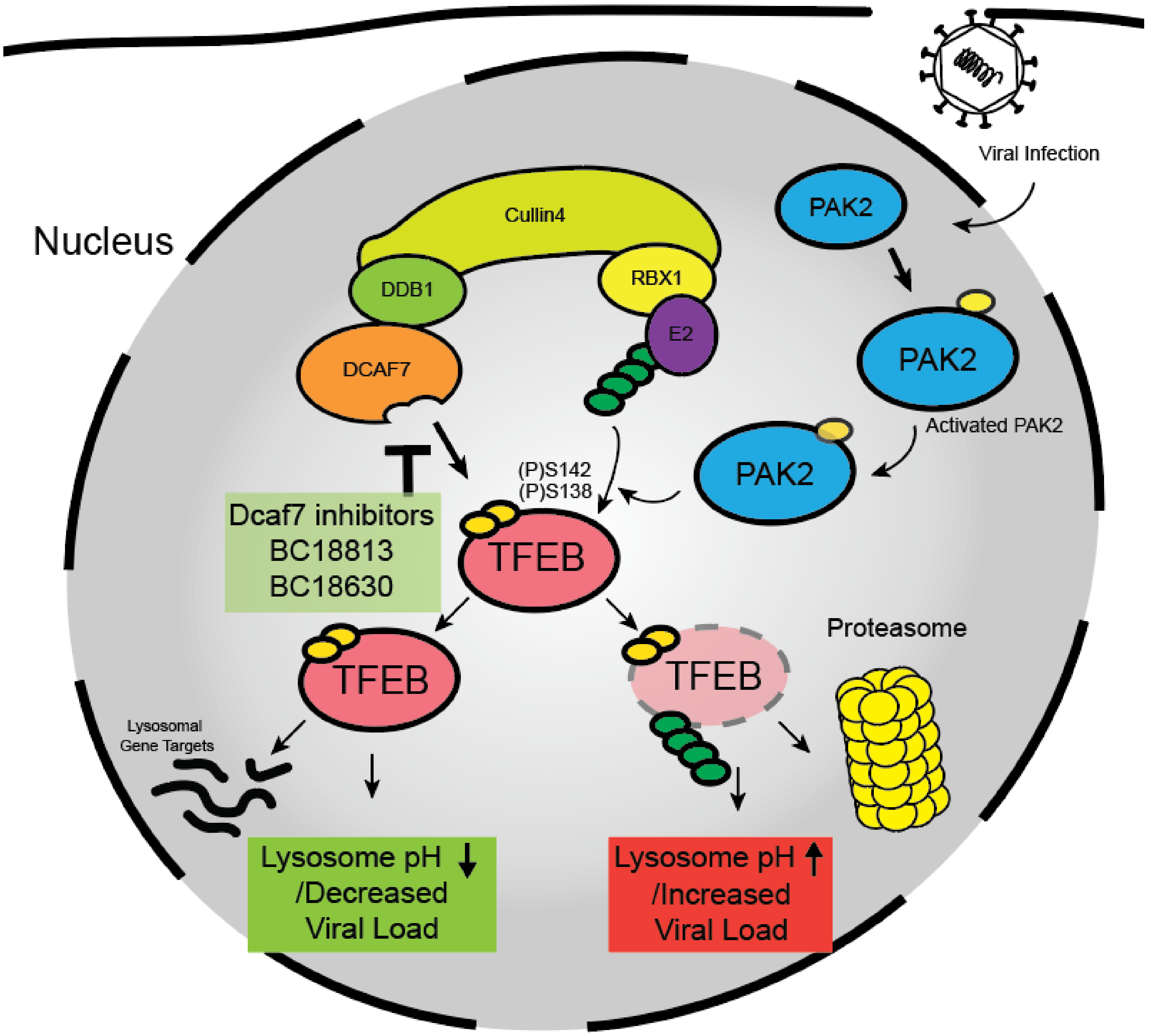

Viral Infection
Nucleus
Cullin4
DDB1
RBX1
E2
DCAF7
PAK2
PAK2
Activated PAK2
PAK2
(P)S142
(P)S138
Dcaf7 inhibitors
BC18813
BC18630
TFEB
TFEB
TFEB
Proteasome
Lysosomal
Gene Targets
Lysosome pH ↓
/Decreased
Viral Load
Lysosome pH ↑
/Increased
Viral Load

Supplemental Table 1. Mass Spectrometry Determination of TFEB Post-translation modification. BEAS-2B cells were treated with vehicle or the proteasomal inhibitor MG132 for 4 hours before collection and processing for mass spectrometry determination of TFEB phosphorylation status. Inhibition of TFEB proteasomal degradation revealed several modified serine residues that may serve as phospho-degron signals for ubiquitination. Note, S138 and S142 were only observed in the presence of MG132.

	Site	Modification	Best Ascore	Localization Probability
Vehicle	S122	Phosphorylation	120.35	1
Carfilzomib	S109	Phosphorylation	37.16	0.548994
	S114	Phosphorylation	42.94	0.999949
	S122	Phosphorylation	120.35	1
	S138	Phosphorylation	17.99	0.993003
	S142	Phosphorylation	17.99	0.993003
	S332	Phosphorylation	20.19	0.980938

Supplemental Table 2. Antibody and Resources

REAGENT or RESOURCE	SOURCE	IDENTIFIER
**Antibodies**		
β-actin	Invitrogen	Cat# MA5-15739, RRID:AB_10979409
GFP (4B10)	Cell Signaling Technologies	Cat# 2955, RRID:AB_1196614
OC43 (541-8F)	EMD Millipore	Cat# MAB9012, RRID:AB_95424
TFEB	Cell Signaling Technologies	Cat# 4240, RRID:AB_11220225
TFEB	Bethyl Laboratories	Cat# A303-673A, RRID:AB_11204751
GAPDH Antibody (ZG003)	Invitrogen	Cat# 39-8600, RRID:AB_2533438
HA Tag (2-2.2.14)	Invitrogen	Cat# 26183, RRID:AB_10978021
V5 Tag	Invitrogen	Cat# R960-25, RRID:AB_2556564
K48-linkage Specific Polyubiquitin	Cell Signaling Technologies	Cat# 4289, RRID:AB_10557239
PAK2 (3B5)	Cell Signaling Technologies	Cat# 4825, RRID:AB_10547135
Phospho-PAK2 (Ser20)	Cell Signaling Technologies	Cat# 2607, RRID:AB_2158759
Anti-WDR68 (DCAF7)	Abcam	Cat# ab70148, RRID:AB_1271483
Phospho-Serine	Millipore	Cat# AB1603, RRID:AB_390205
Lamin A/C (4C11)	Cell Signaling Technologies	Cat# 4777, RRID:AB_10545756
Cyclin E (HE111)	Santa Cruz Biotechnology	Cat# sc-248, RRID:AB_627362
Dnmt (H-12)	Santa Cruz Biotechnology	Cat# sc-271729, RRID:AB_10710384
PTEN (A2B1)	Santa Cruz Biotechnology	Cat# sc-7974, RRID:AB_628187
Influenza A Virus Nucleoprotein (C43)	Abcam	Cat# ab128193, RRID:AB_11143769
p21 Waf1/Cip1 (12D1)	Cell Signaling Technologies	Cat# 2947, RRID:AB_823586
SARS-CoV-2 Nucleoprotein	Invitrogen	Cat# MA1-7403, RRID:AB_1018420
TFEB (C-6)	Santa Cruz Biotechnology	Cat# sc-166736, RRID:AB_2255943
DCAF7	Invitrogen	Cat# PA5-54642, RRID:AB_2640409
LAMP1	Novus	Cat# NB100-77683, RRID:AB_1083191
Goat anti-Rat IgG (H+L) Cross-Adsorbed Secondary Antibody, Alexa Fluor 488	Invitrogen	Cat# A-11006, RRID:AB_2534074)
Goat anti-Rabbit IgG (H+L), Superclonal Recombinant Secondary Antibody, Alexa Fluor 647	Invitrogen	Cat# A27040, RRID:AB_2536101
Goat anti-Rabbit IgG (H+L) HRP	Invitrogen	Cat# 31460, RRID:AB_228341
Goat anti-Mouse IgG (H+L) HRP	Invitrogen	Cat# 31430, RRID:AB_228307

Bacterial and Virus Strains		
TOP10	Invitrogen	C404010
Pseudomonas aeruginosa	ATCC	Strain PA103
Staphylococcus aureus	ATCC	Strain 29213
Stbl3	Invitrogen	C737303
Human Betacoronavirus 1 (OC43)	ATCC	VR-1558
Human coronavirus 229E	ATCC	VR-740
Influenza A virus (H1N1)	ATCC	VR-99
SARS-CoV-2 (in vitro)	IIT Research Institute (Chicago, USA)	USA-WA1/2020
SARS-CoV-2 (in vivo)	ChemDIV (San Diego, USA)	Victoria/1/2020
**Chemicals, Peptides, and Recombinant Proteins**		
FDA-Approved Compound Library	Selleck	L1300
BC1753	ChemDiv	
BC18813	This Study	
BC18630	This Study	
Carfilzomib	Cayman Chemical	17554
Leupeptin	Cayman Chemical	14026
MG132	UBPBio	F1101
Bafilomycin A1	Cayman Chemical	11038
LysoTracker Red DND-99	Invitrogen	L7528
CellTracker Green CMFDA	Invitrogen	C2925
Lysosensor Yellow/Blue DND-160	Invitrogen	L7545
Hoechst33342	Invitrogen	H3570
DMEM/F-12	Gibco	11320082
EMEM	Gibco	670086
Fetal Bovine Serum	Gibco	26140079
Opti-MEM I Reduced Serum Medium	Gibco	31985062
Critical Commercial Assays		
pcDNA3.1 Directional TOPO Expression Kit	Invitrogen	K490001
Duolink In Situ Orange Starter Kit Mouse/Rabbit	MilliporeSigma	DUO92102
Dynabeads His-Tag Isolation and Pulldown	Invitrogen	10103D
Pierce Protein A/G Magnetic Beads	Thermo Scientific	88802
High-Capacity cDNA Reverse Transcription Kit	Applied Biosystems	4368814
SYBR Green PCR Master Mix	Applied Biosystems	4364344
In-Fusion Cloning	Takara	638910
Gateway Cloning	ThermoFisher	11791019
Phusion polymerase	New England Biolabs	M0530
Quick CIP	New England Biolabs	M0525
Quick Ligation Kit	New England Biolabs	M2200
Easy Prep RNA Miniprep Plus Kit	Bioland Scientific	R01-04
RNeasy Plus Mini Kit	Qiagen	74134
DC Protein Assay Reagent A/B/S	BioRad	500-0113/ 0114/ 0115
GFP-Trap Magnetic Agarose	ChromoTek	gtma-20
NE-PER Nuclear and Cytoplasmic Extraction Reagents	Thermo Scientific	78833
QuikChange II XL Site-Directed Mutagenesis Kit	Aglient	200521
X-tremeGENE siRNA Transfection Reagent	Sigma-Aldrich	4476115001
X-tremeGENE HP DNA Transfection Reagent	Sigma-Aldrich	6366244001
Lipofectamine 3000 Transfection Reagents	Invitrogen	L3000015
MagicRed Cathepsin B activity assay	ImmunoChemistry Technologies	937
CellTiter-Glo 2.0 Cell Viability Assay	Promega	G9243
TnT Quick Coupled Transcription/Translation kit	Promega	L1170

Experimental Models: Cell Lines		
MLE-12	ATCC	CRL-2110
BEAS-2B	ATCC	CRL-9609
BEAS-2B TFEB-EGFP stable	This Study	
BEAS-2B DCAF7 KO	This Study	
BEAS-2B DCAF7 KO, Re-expressed DCAF7	This Study	
BEAS-2B PAK2 KO	This Study	
BEAS-2B GFP-LC3	This Study	
HEK293A GFP-LC3 cell	Sigma	14050801
MRC-5	ATCC	CCL-171
HCT-8	ATCC	CCL-244
HEK293T	ATCC	CRL-1573
Calu-3	ATCC	HTB-55
Experimental Models: Organisms/Strains		
C57BL/6J male mice	Jackson Laboratories	
Sprague-Dawley rats	Touchstone Biosciences (in house)	
Syrian hamsters	ChemDIV (in house)	
**Recombinant DNA**		
Item	Source	Identifier
pLENTI-c-mGFP-P2A-Puro	Origene	PS100093
WT-DCAF7, pcDNA3.1D-V5-HIS	This study	
WT-DCAF7, pcDNA3.1D-HA	This study	
WT-TFEB, pcDNA3.1D-V5-HIS	This study	
Δ1-115 TFEB, pcDNA3.1D-V5-HIS	This study	
Δ256-476 TFEB, pcDNA3.1D-V5-HIS	This study	
Δ310-476 TFEB, pcDNA3.1D-V5-HIS	This study	
Δ290-476 TFEB, pcDNA3.1D-V5-HIS	This study	
Δ274-476 TFEB, pcDNA3.1D-V5-HIS	This study	
S138A TFEB, pcDNA3.1D-V5-HIS	This study	
S142A TFEB, pcDNA3.1D-V5-HIS	This study	
K232R TFEB, pcDNA3.1D-V5-HIS	This study	
S138A/S138A TFEB, pcDNA3.1D-V5-HIS	This study	
WT-TFEB, pEGFP-N1	Plasmid was a gift from Shawn Ferguson [14]	Addgene plasmid: 38119
S138A TFEB, pEGFP-N1	This study	
S142A TFEB, pEGFP-N1	This study	
S138A/S138A TFEB, pEGFP-N1	This study	
pRK5-HA-Ubiquitin-WT	Plasmid was a gift from Ted Dawson [6]	Addgene plasmid: 17608
pLenti-CMV-Neo ss-Keima-LAMP1-mGFP	This study	
lentiCRISPR v2	Plasmid was a gift from Feng Zhang [3]	Addgene plasmid: 52961
psPAX2	Plasmid was a gift from Didier Trono	Addgene plasmid: 12260
pMD2.G	Plasmid was a gift from Didier Trono	Addgene plasmid: 12259
LAMP1-mGFP	Plasmid was a gift from Esteban Dell'Angelica[15]	Addgene plasmid: 34831
Software and Algorithms		
Fiji ImageJ	[8]	
Graphpad	Prism	9.0

Gen5	Biotek	3.0
Discovery Studio	BIOVIA	3.5
CellProfiler	[7]	3.0

Supplemental Table 3. Oligonucleotides

Oligonucleotides	Sequence	Source
Ctrl siRNA	Control DsiRNA Negative Control (DS NC1)<br>rCrGrUrUrArArUrCrGrCrGrUrArUrArArUrArCrGrCrGrUAT<br>rArUrArCrGrCrGrUrArUrUrArUrArCrGrCrGrArUrUrArArCrGrArC	IDT
DCAF7 siRNA	hs.Ri.DCAF7.13.1<br>rCrCrUrCrArArGrCrArUrUrGrArUrArCrGrArCrArUrGrCAC<br>rGrUrGrCrArUrGrUrCrGrUrArUrCrArArUrGrCrUrUrGrArGrGrUrA	IDT
PAK2 siRNA	hs.Ri.PAK2.13.1<br>rGrGrUrGrArGrCrUrCrUrGrArUrArCrArGrUrGrArGrArAGA<br>rUrCrUrUrCrUrCrArCrUrGrUrArUrCrArGrArGrCrUrCrArCrCrArA	IDT
DCAF7 sgRNA	GCGGTGACTATCTCCGTGTG	IDT
PAK2 sgRNA	GTGTGCTCAAAATCAGATGG	IDT
MISSION® siRNA Human Gene Family Set	Kinase panel, 719 targets from Sigma-Aldrich	SI02100
WT TFEB in pcDNA3.1D-V5-HIS	Fwd: caccATGGCGTCACGCATAGGGT<br>Rev: CAGCACATCGCCCTCCTC	
TFEB S138A	Fwd: CTCCGCTGGCAACGCTGCTCCCAATAG<br>Rev: CTATTGGGAGCAGCGTTGCCAGCGGAG	IDT
TFEB S142A	Fwd: CAACAGTGCTCCCAATGCACCCATGGCCATGCTGC<br>Rev: GCAGCATGGCCATGGGTGCATTGGGAGCACTGTTG	IDT
TFEB S138A/S142A	Fwd: CTCCGCTGGCAACGCAGCTCCCAATGCAC (using S142A as template)<br>Rev: GTGCATTGGGAGCTGCGTTGCCAGCGGAG (using S142A as template)	IDT
TFEB to K232R	Fwd: GGCCCTGGCCAGAGAGCGGCAGAAGAAAGACAAT<br>Rev: ATTGTCTTTCTTCTGCCGCTCTCTGGCCAGGGCC	IDT
Δ1-115 TFEB in pcDNA3.1D-V5-HIS	Fwd: caccATGCCCCCACCAGCC<br>Rev: CAGCACATCGCCCTCCTC	IDT
Δ310-476 TFEB in pcDNA3.1D-V5-HIS	Fwd: caccATGGCGTCACGCATAGGGT<br>Rev: GTTGGTCATCTCCAGGCGG	IDT
Δ290-476 TFEB in pcDNA3.1D-V5-HIS	Fwd: caccATGGCGTCACGCATAGGGT<br>Rev: CTGCATCCTCCGGATGTAATCC	IDT
Δ274-476 TFEB in pcDNA3.1D-V5-HIS	Fwd: caccATGGCGTCACGCATAGGGT<br>Rev: GTTCCAGCGCACGTCCA	IDT
Δ256-476 TFEB in pcDNA3.1D-V5-HIS	Fwd: caccATGGCGTCACGCATAGGGT<br>Rev: GATGCGGTCATTGATGTTGAAC	IDT
WT DCAF7 in pcDNA3.1D-V5-HIS	Fwd: cacc ATGTCCCTGCACGGCAAAC<br>Rev: CACTCTGAGTATCTCCAGGCAGTTGTT	IDT
DCAF7 C-terminal HA-tag in pcDNA3.1D-V5-HIS	Fwd: cacc ATGTCCCTGCACGGCAAAC<br>Rev: TTA agcgtaatctggaacatcgtatgggta CACTCTGAGTATCTCCAGGCAGTTGTT	IDT
qPCR CTSA	Fwd: CAGGCTTTGGTCTTCTCTCCA<br>Rev: TCACGCATTCCAGGTCTTTG	IDT
qPCR CTSD	Fwd: AACTGCTGGACATCGCTTGCT<br>Rev: CATTCTTCACGTAGGTGCTGGA	IDT
qPCR HEXA	Fwd: CAACCAACACATTCTTCTCCA<br>Rev: CGCTATCGTGACCTGCTTTT	IDT
qPCR MCOLN1	Fwd: TTGCTCTCTGCCAGCGGTACTA<br>Rev: GCAGTCAGTAACCACCATCGGA	IDT

qPCR SQSTM1	Fwd: AAGCTGCCTTGTACCCAC<br>Rev: CGCTCCGATGTCATAGTTCTTG	IDT
qPCR WIPI1	Fwd: CTTCAAGCTGGAACAGGTCACC<br>Rev: CGGAGAAGTTCAAGCGTGCAGT	IDT
qPCR GAPDH	Fwd: TGCACCACCAACTGCTTAGC<br>Rev: GGCATGGACTGTGGTCATGAG	IDT
qPCR Actin	Fwd: CACCATTGGCAATGAGCGGTTC<br>Rev: AGGTCTTTGCGGATGTCCACGT	IDT
qPCR TFEB	Fwd: CAATACCCCCGTCCACTTCC<br>Rev: CCAGGTAGGACTGCACCTTC	IDT
qPCR 229E	Fwd: CAC TCG TTA TGT GGG TGA TG<br>Rev: CAC GGT TGT GAC AGT GAT T<br>Probe: 56-FAM/TT GGG CAT GGA ATC CTG AGG TCA A/36-TAMSp	IDT
qPCR OC43	Fwd: ATG TTA GGC CGA TAA TTG AGG ACT AT<br>Rev: AAT GTA AAG ATG GCC GCG TAT T<br>Probe: 56-FAM/CA TAC TCT GAC GGT CAC AAT /36-TAMSp/	IDT
Primers used for SARS-CoV-2 detection	IITRI<br>2019-nCoV_N1-F 5’-GACCCCAAAATCAGCGAAAT-3’<br>2019-nCoV_N1-R 5’-TCTGGTTACTGCCAGTTGAATCTG-3’<br>Probe:<br>2019-nCoV_N1-P<br>5’-FAM-ACCCCGCATTACGTTTGGTGGACC-BHQ1-3’.	N/A
Monomeric Keima_fwd	GTGAGCGTGATCGCCAAGCAG	This Study
Monomeric Keima_rev	TTTCACCATAAACATGCCCAGCAGGGAGTG	This Study
LAMP1 ss (1 – 29 aa)_fwd	CTCAAGCTTCGAATTACCATGGCGGCCCCCGGCAGCGC	This Study
LAMP1 ss (1 – 29 aa)_rev	GGCGATCACGCTCACTGCTGCTGACGCACAATGC	This Study
LAMP1 (30 – 417 aa)_fwd	ATGTTTATGGTGAAAAATGGC	This Study
LAMP1 (30 – 417 aa)_rev	GTCGACTGCAGAATTTCACTTGTACAGCTCGTC	This Study

Uncropped Blots

Fig. 1A

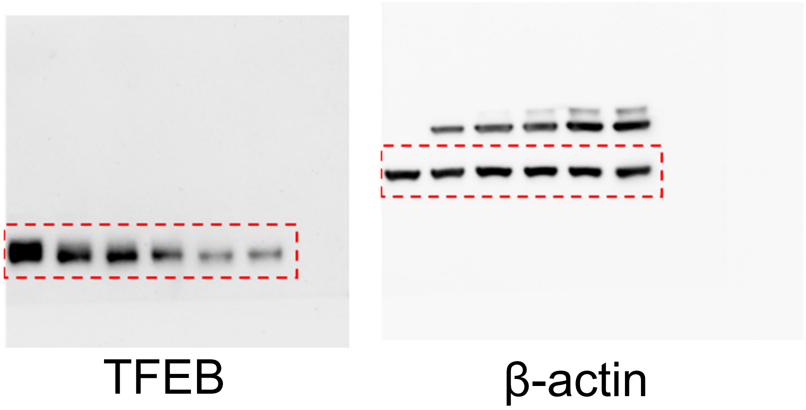

Fig. 1B

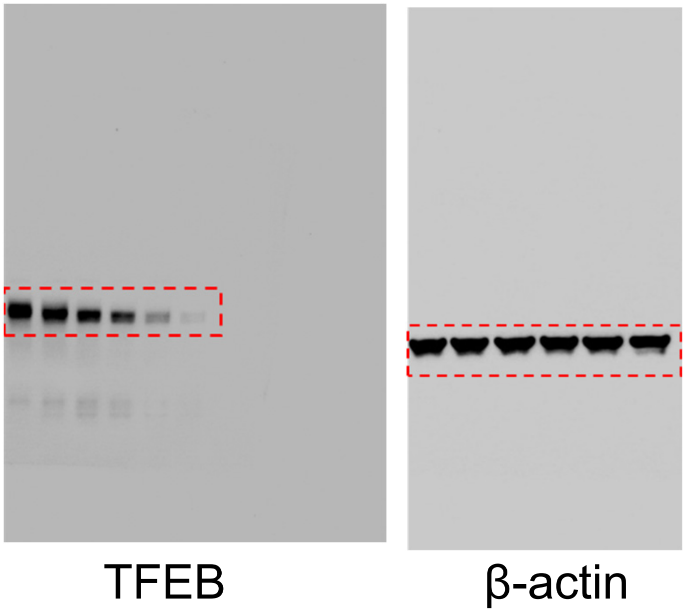

Fig. 1E

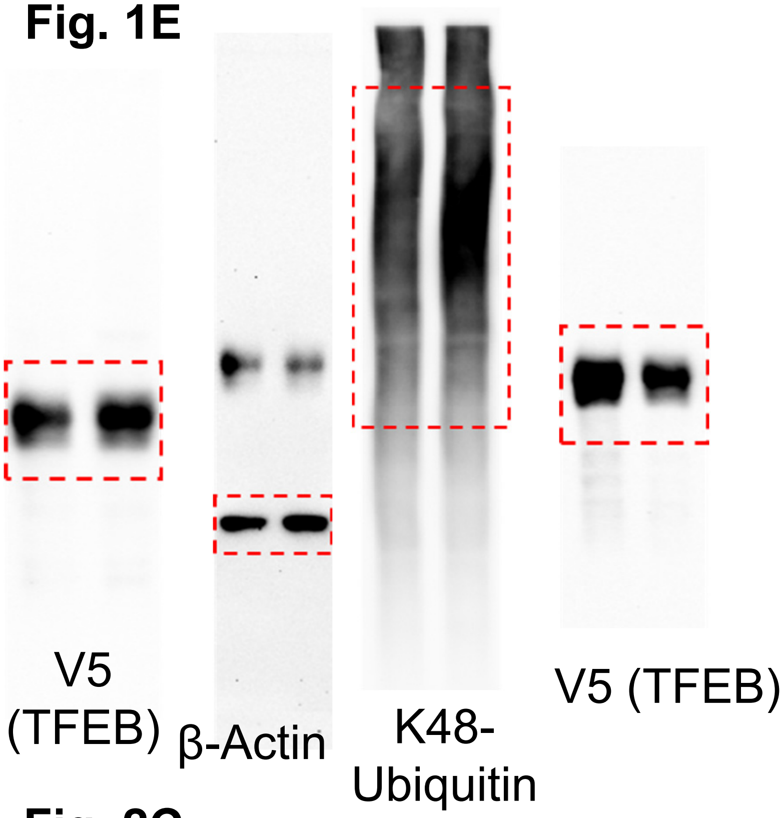

Fig. 2B

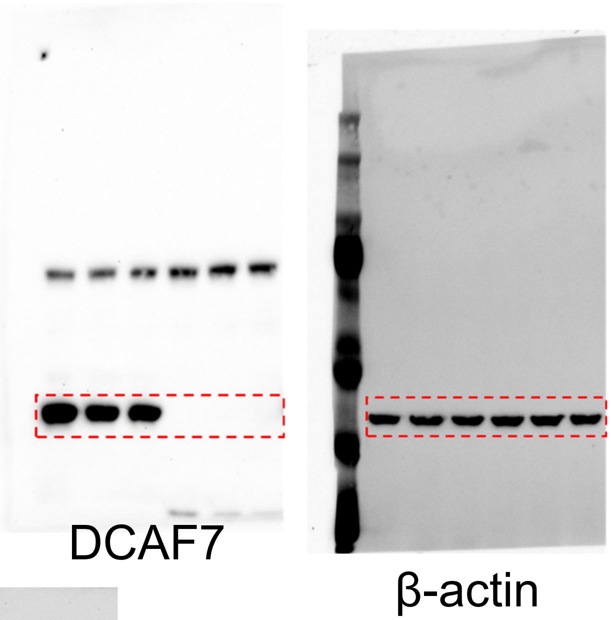

Fig. 2C

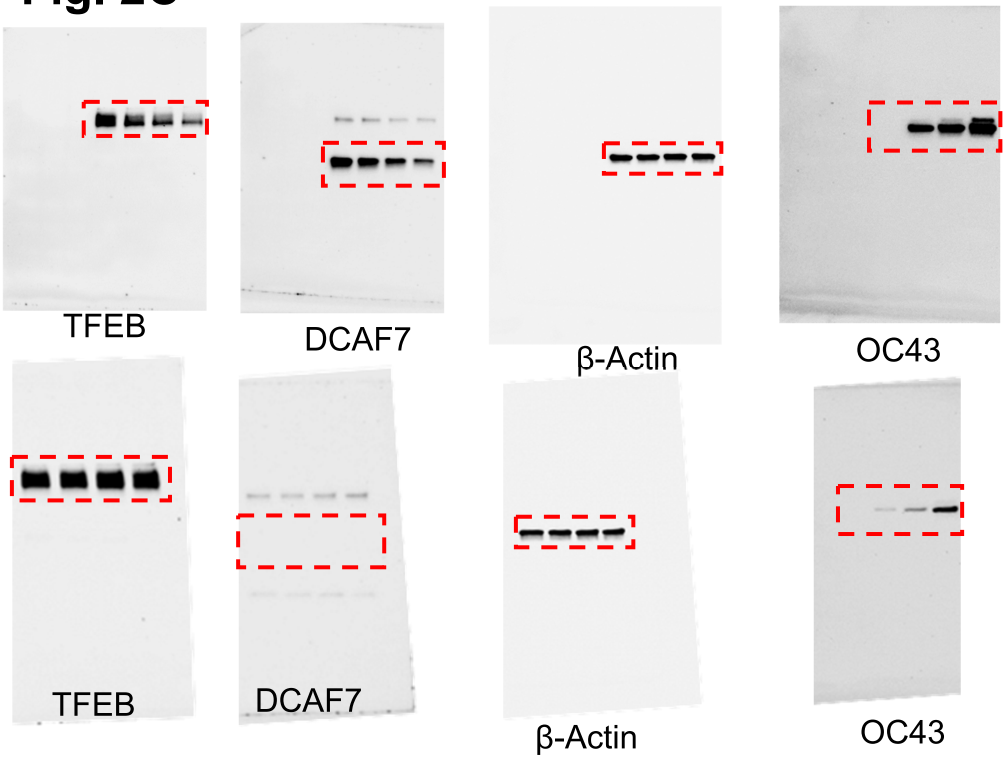

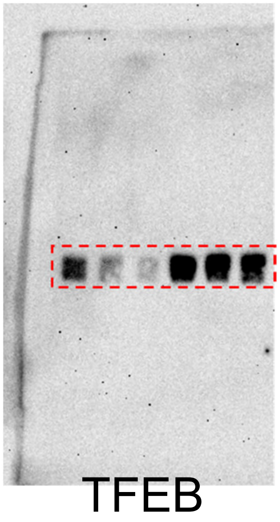

Fig. 3A

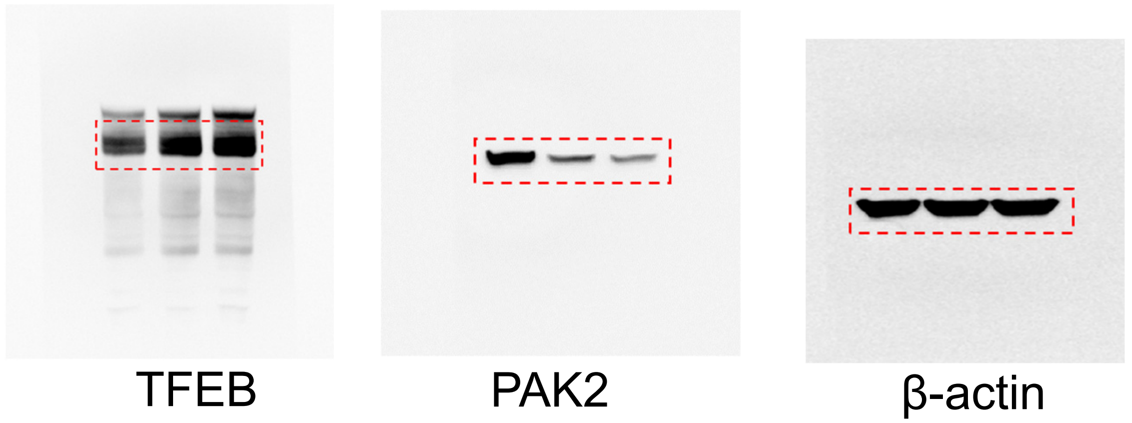

Fig. 3C

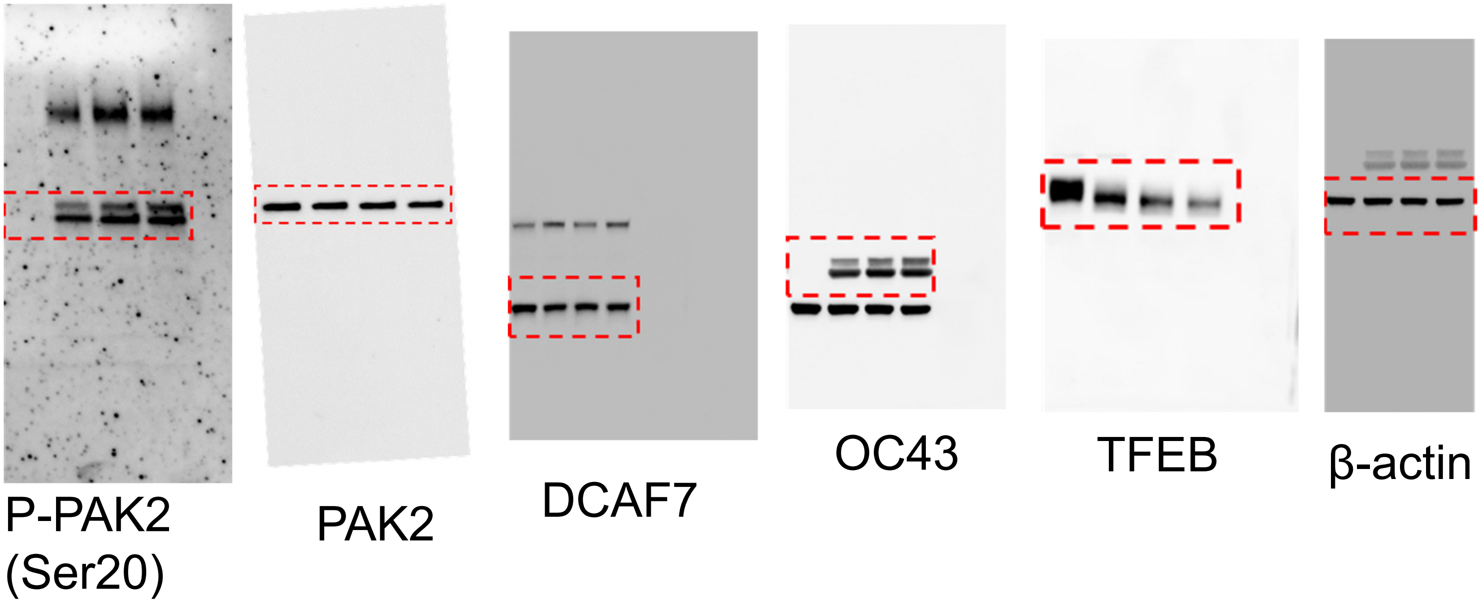

Fig. 3E

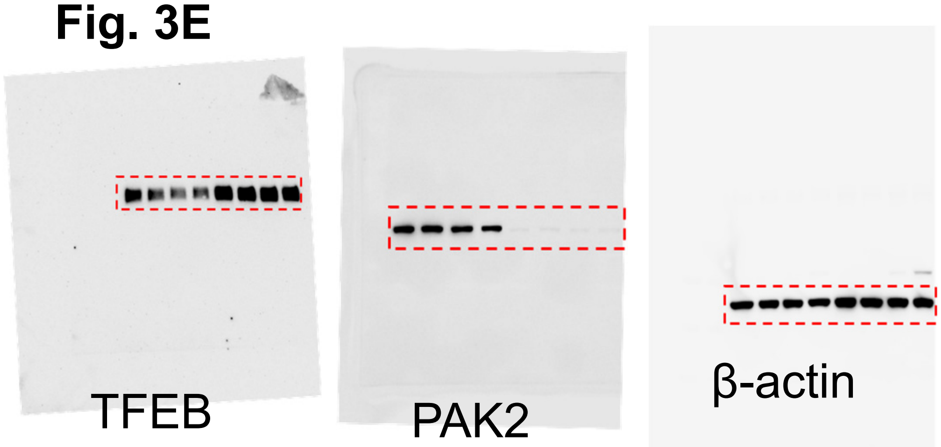

Fig. 3I

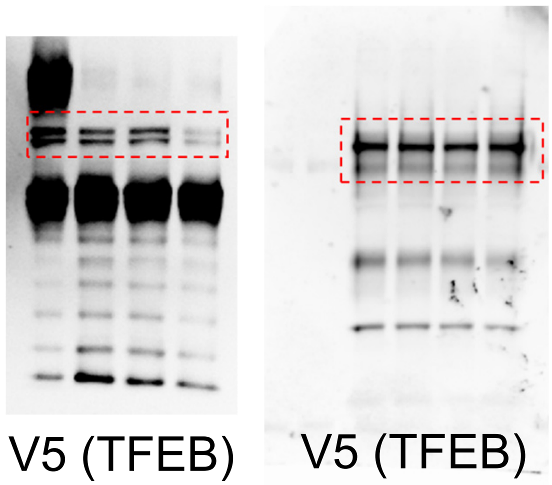

Fig. 3J

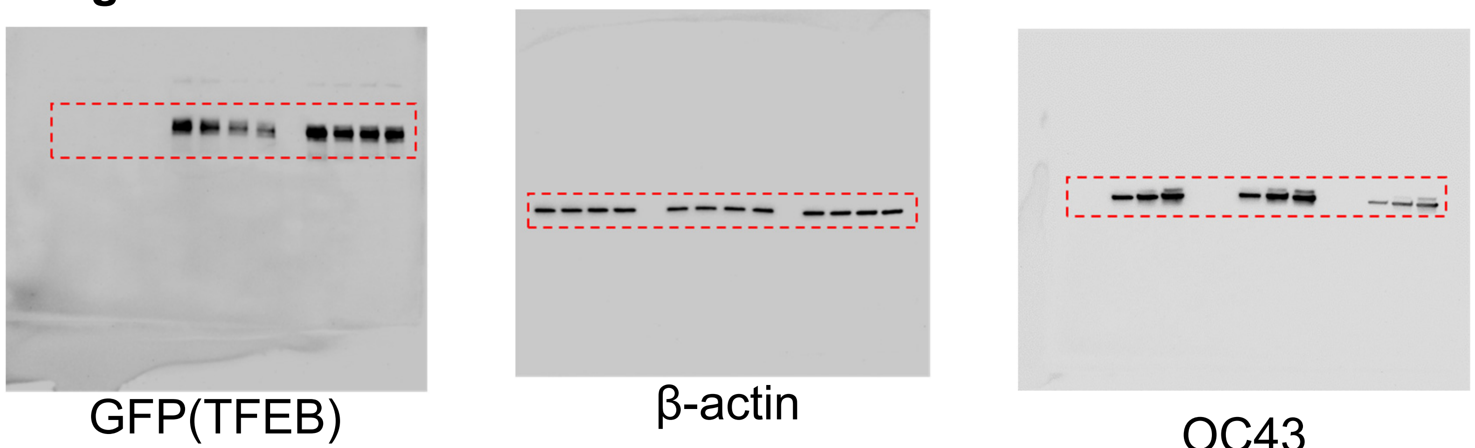

Fig. 4C

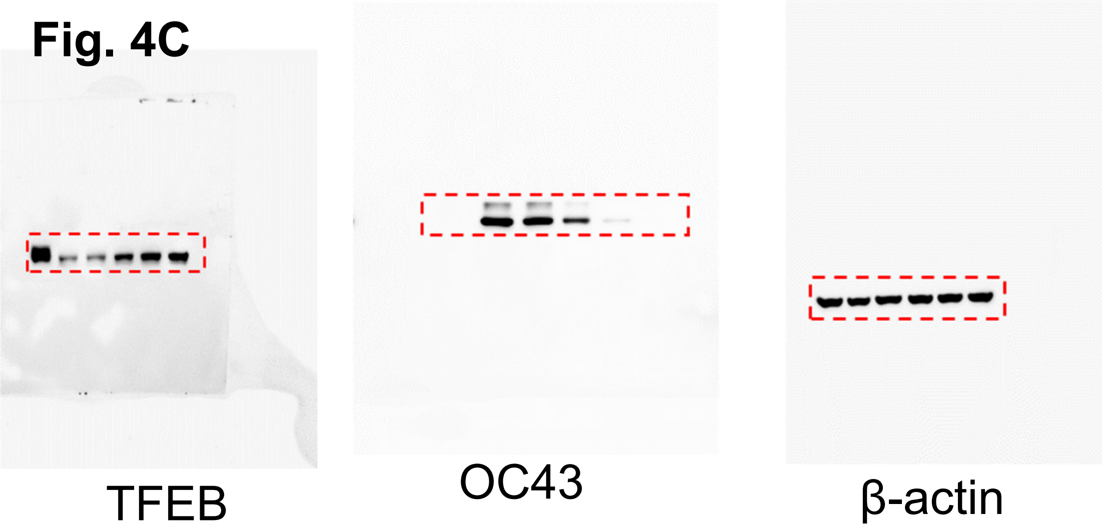

Fig. S1B

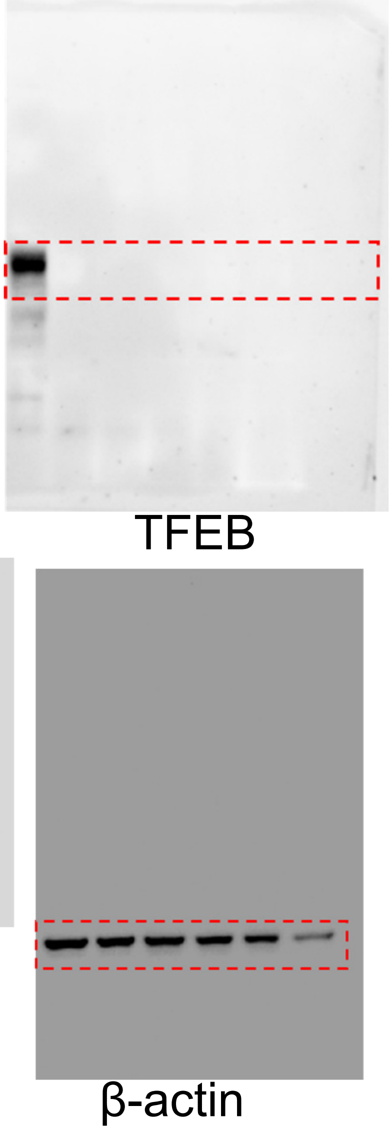

Fig. S1A

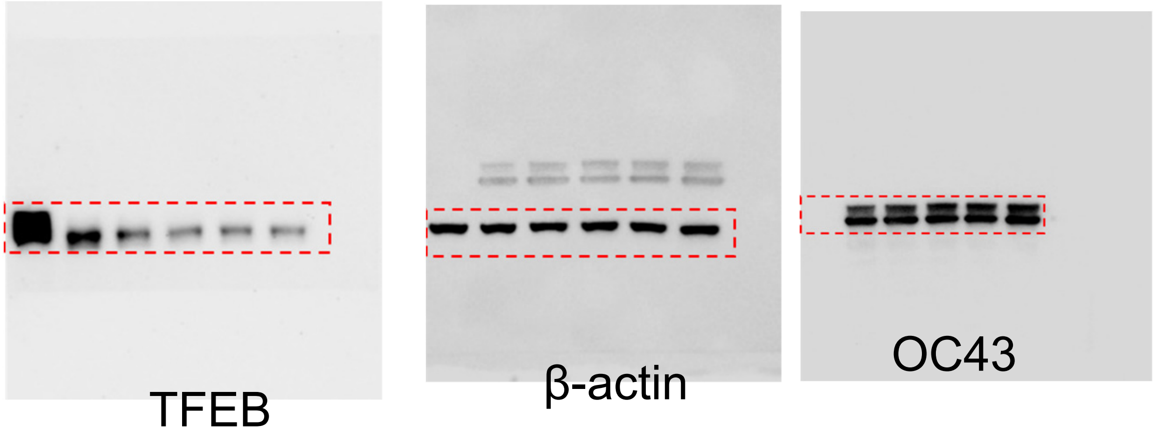

Fig. S1E

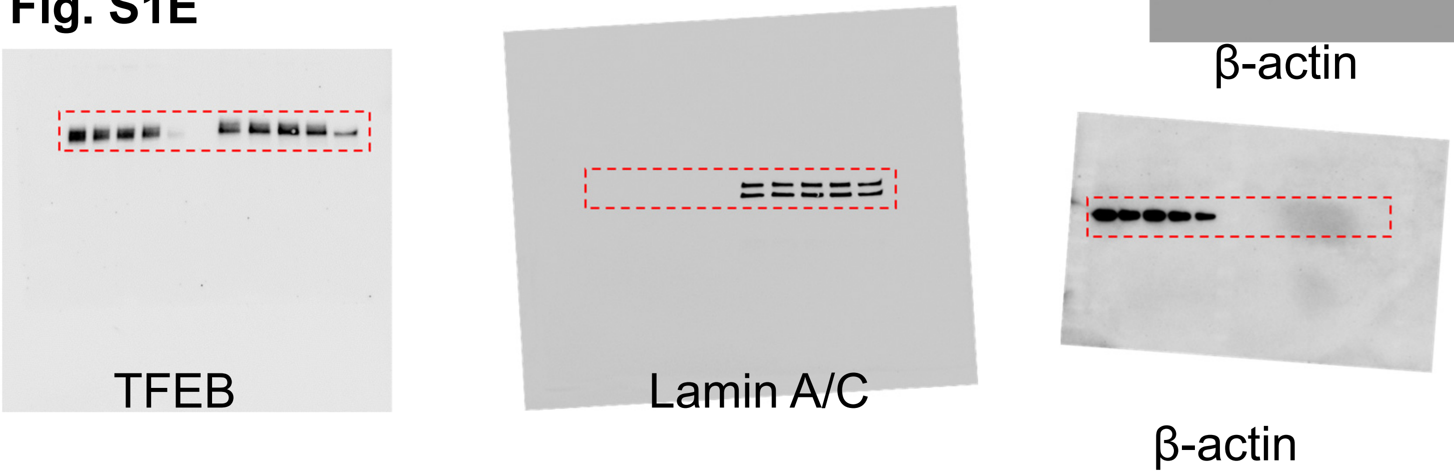

Fig. S1F

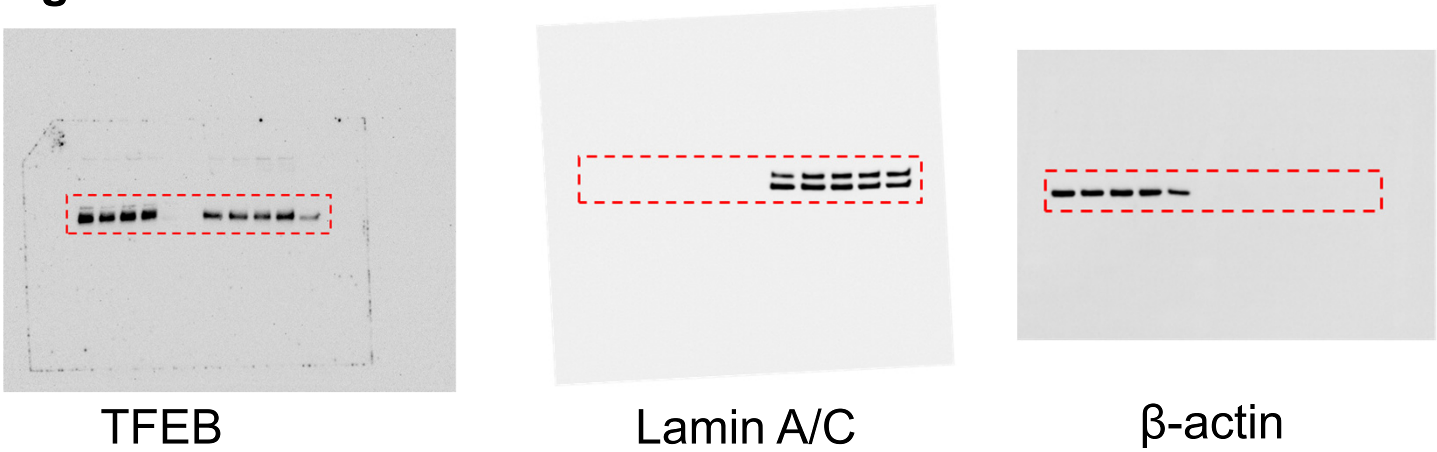

Fig. 2A

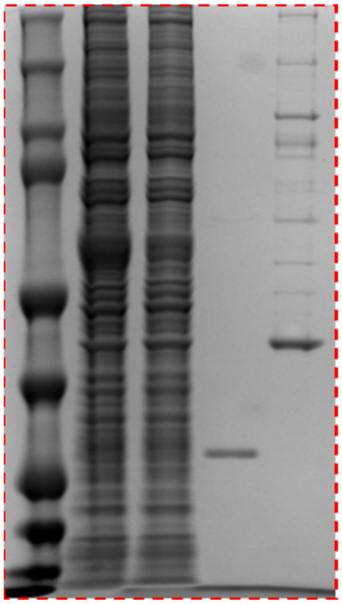

Coomassie Blue

Fig. 2B

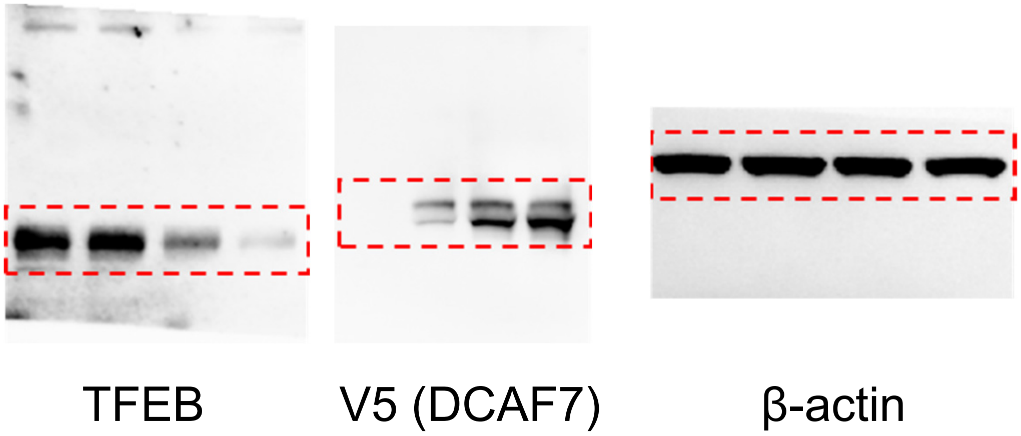

Fig. 2C

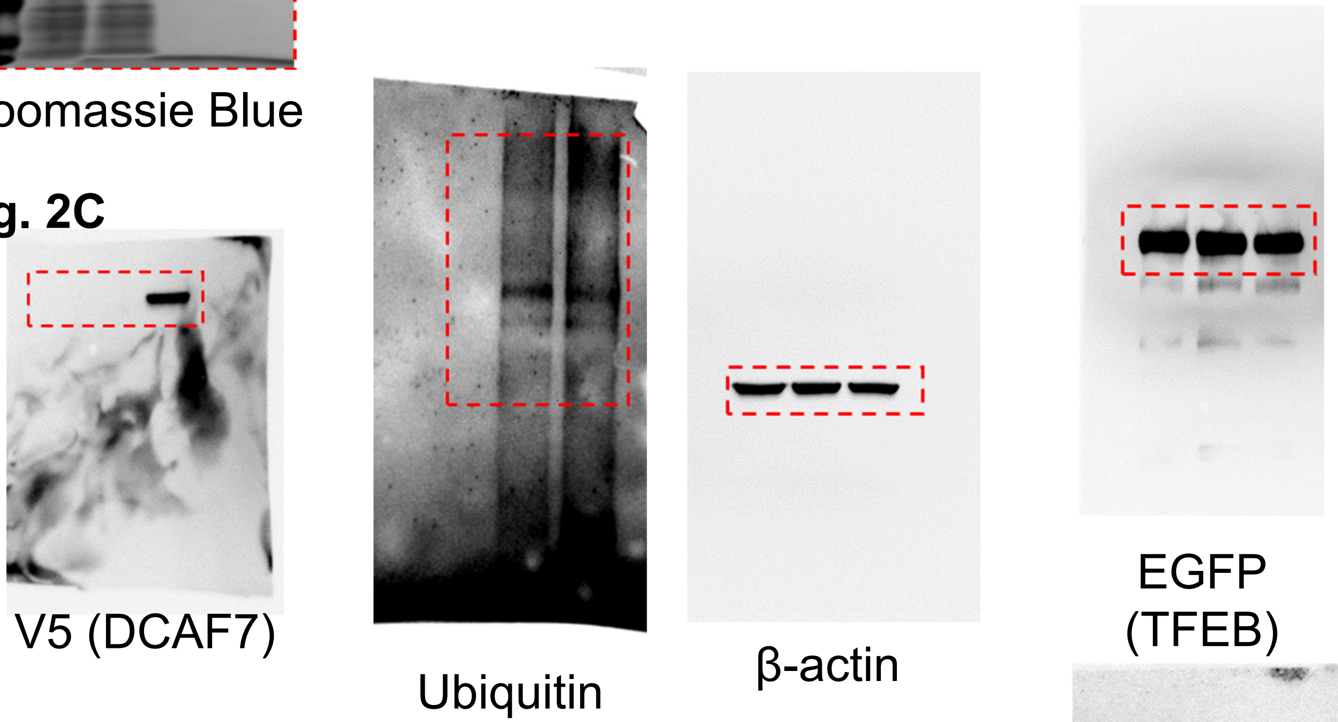

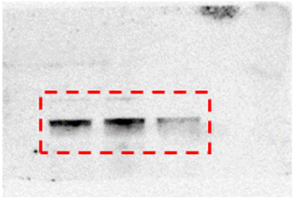

EGFP (TFEB)

Fig. S2E

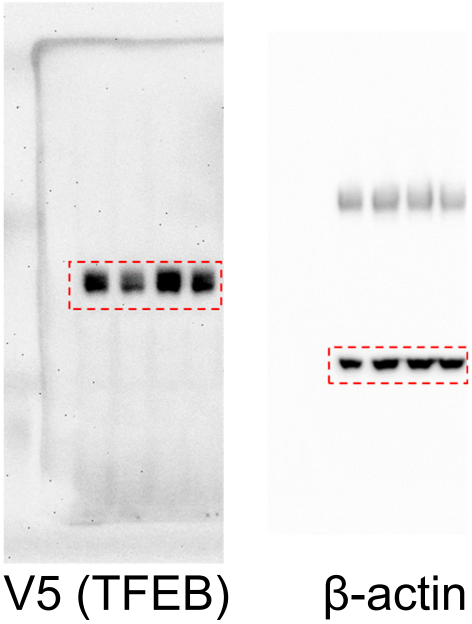

Fig. S2F

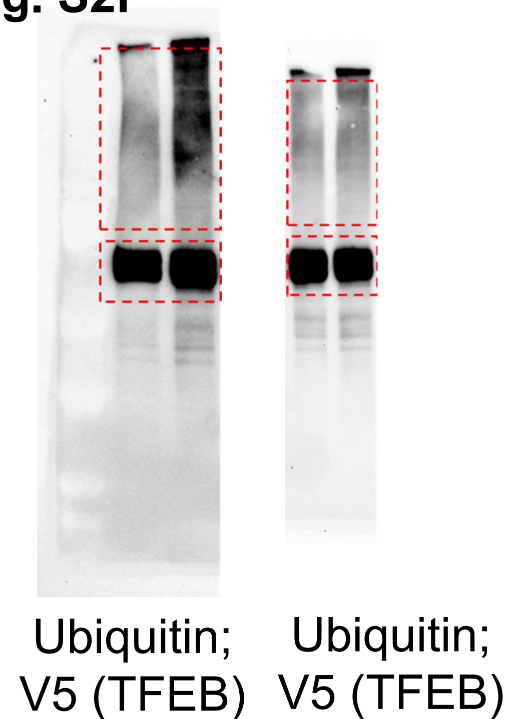

Fig. S2G

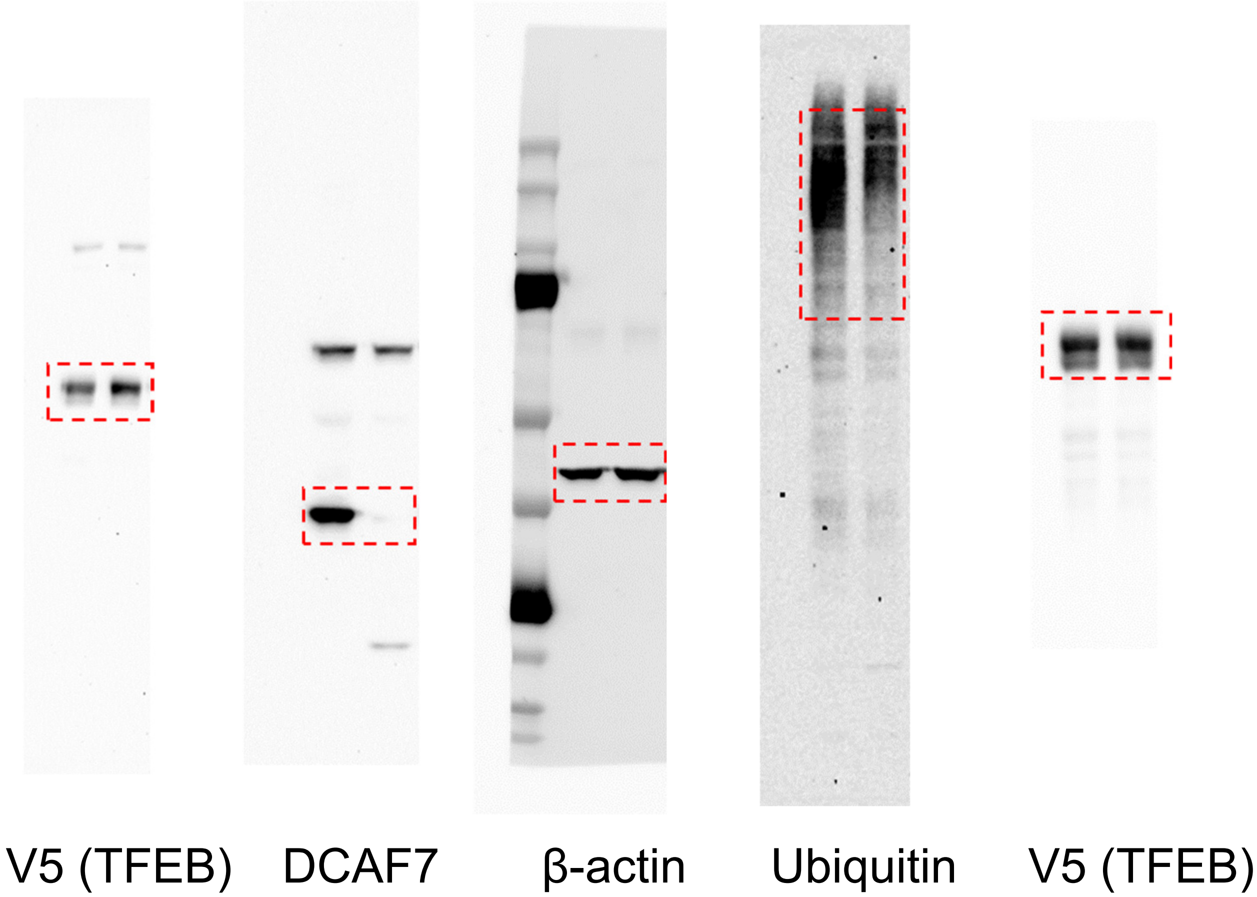

Fig. S3B

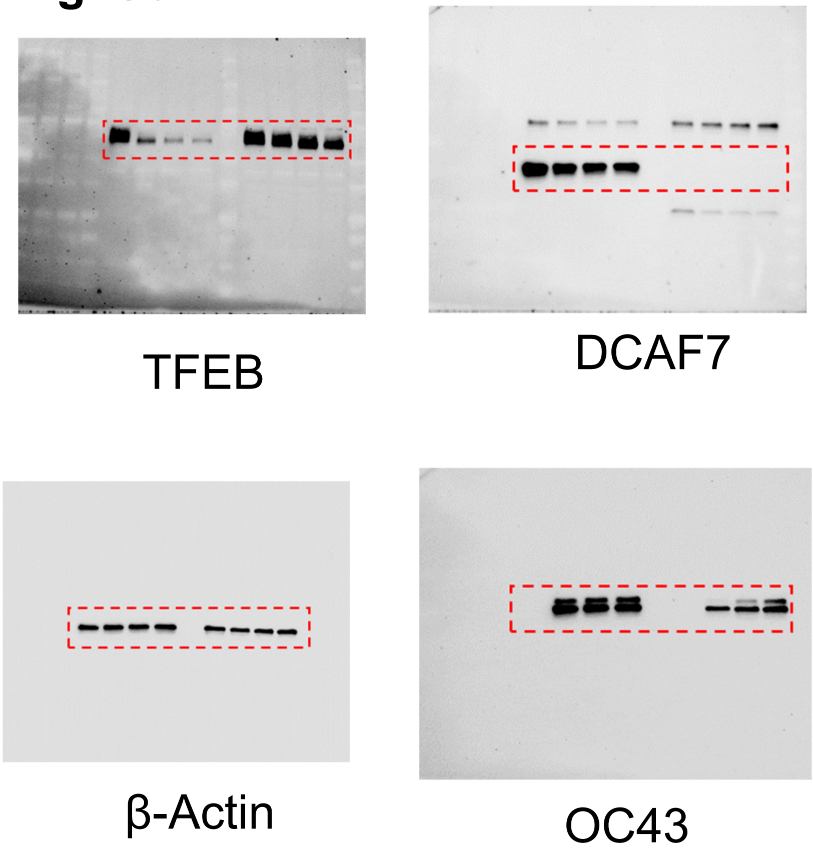

Fig. S3E

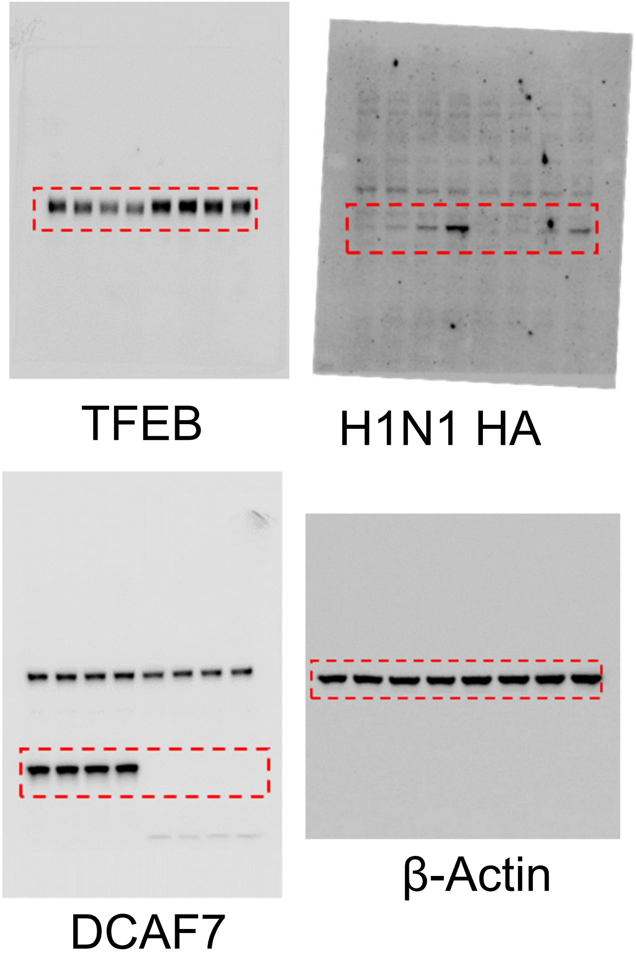

Fig. S4A

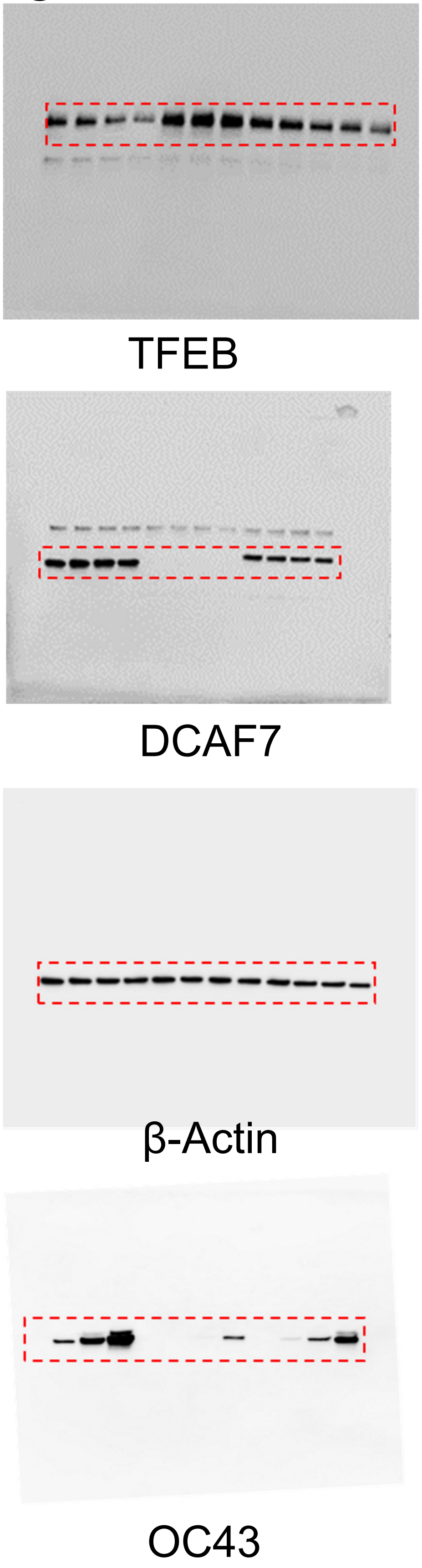

Fig. S5C

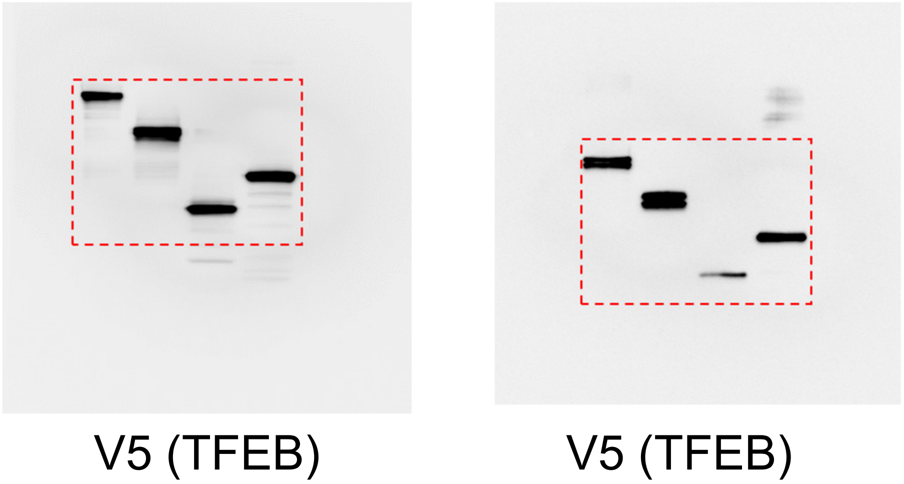

Fig. S5D

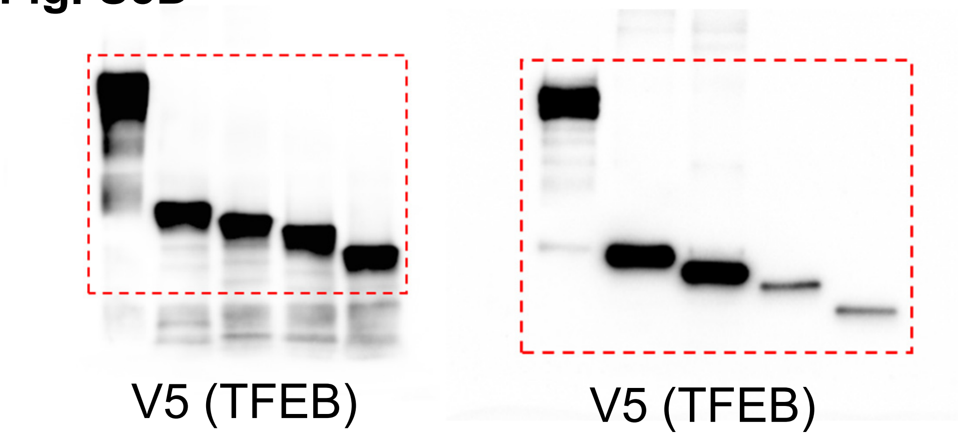

Fig. S6B

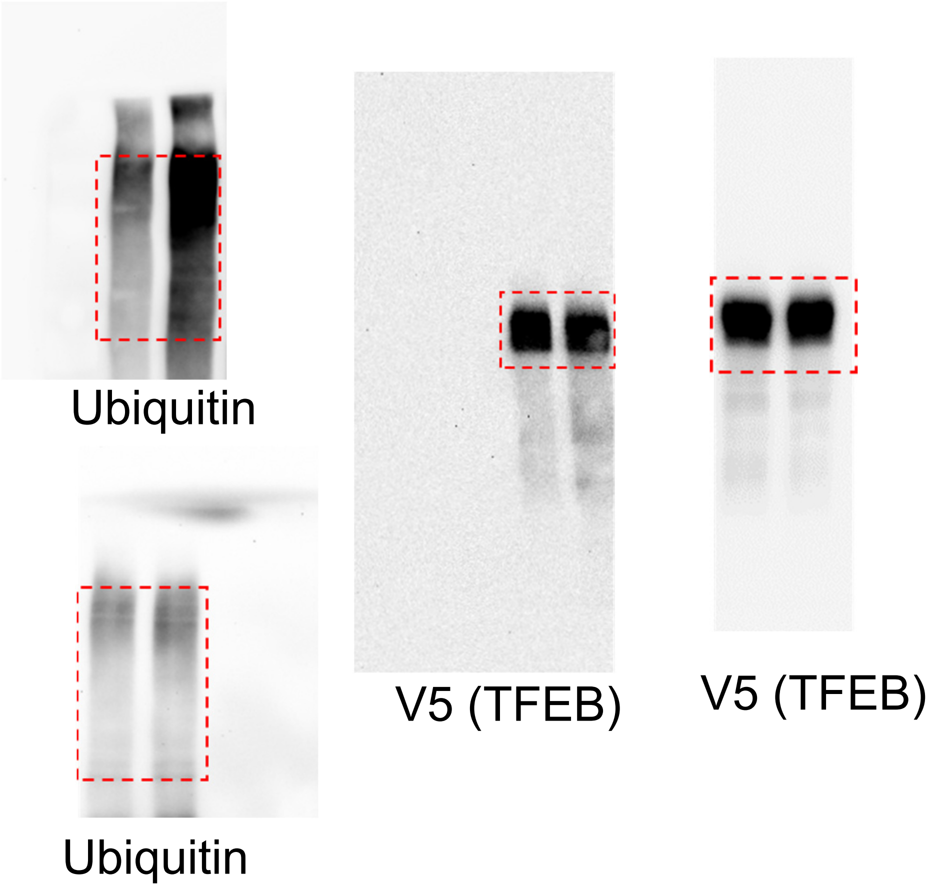

Fig. S6D

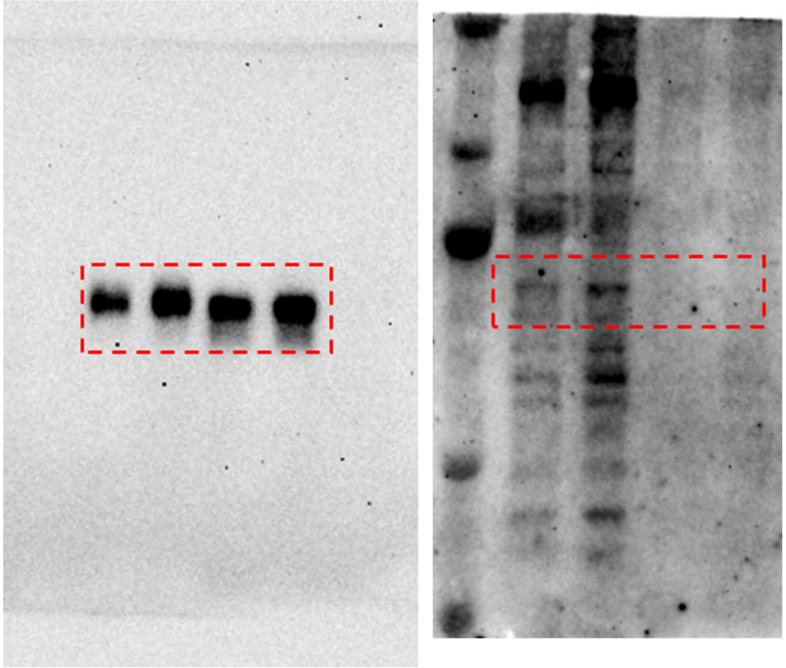

V5 (TFEB)

(P)Serine

Fig. S7B

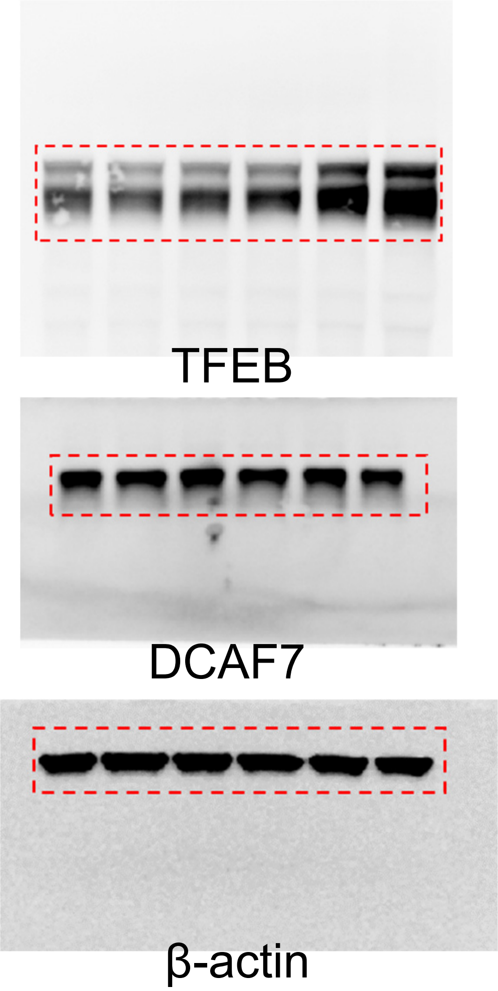

Fig. S8B

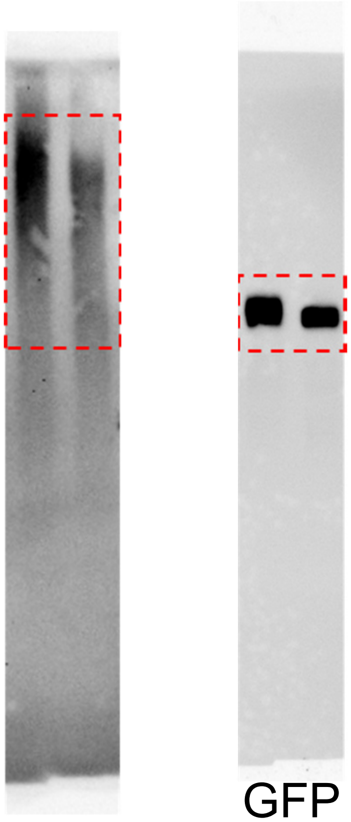

Ubiquitin

GFP (TFEB)

Fig. S8C

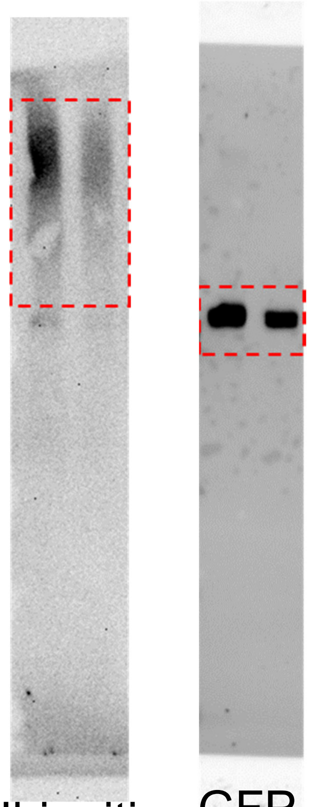

Ubiquitin

GFP (TFEB)

Fig. S12A

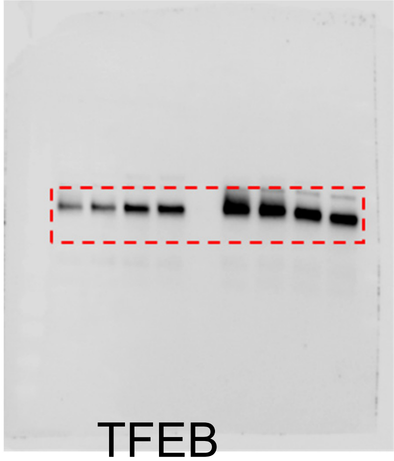
TFEB

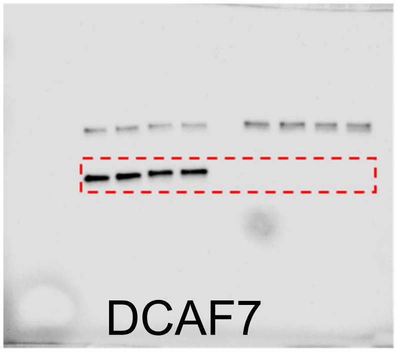
DCAF7

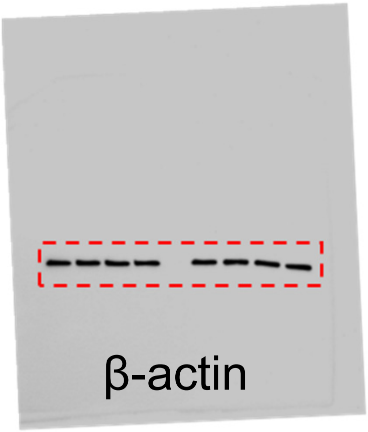
β-actin

Fig. S13A

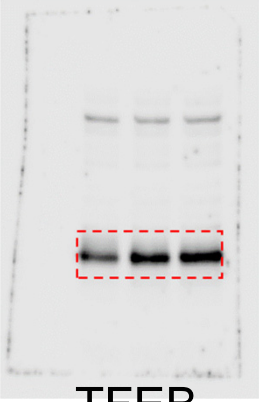
TFEB

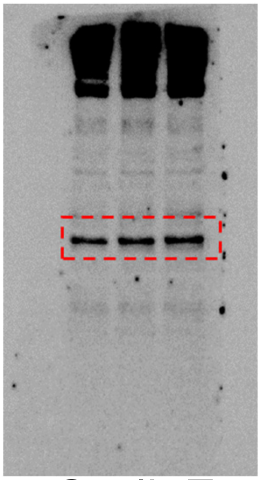
CyclinE

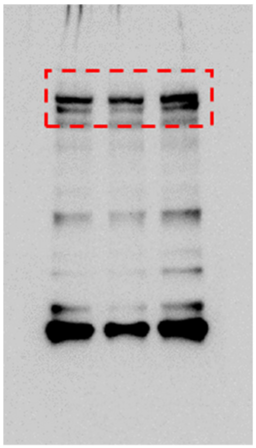
DNMT

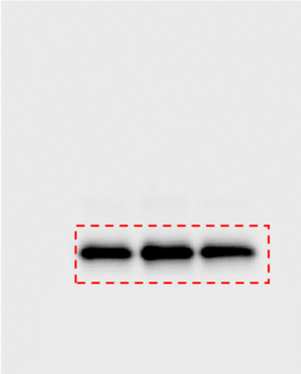
P21

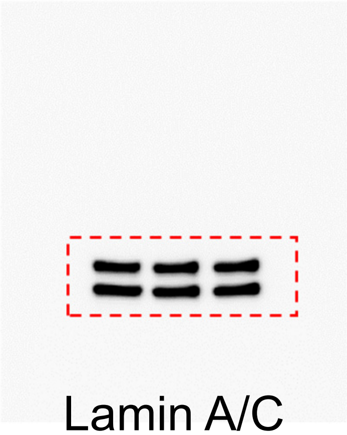
Lamin A/C

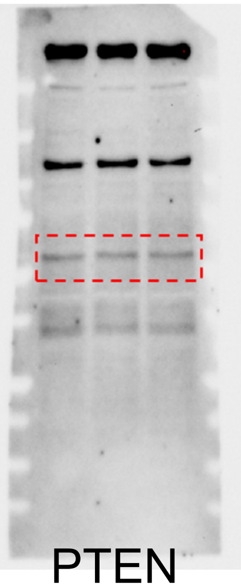
PTEN

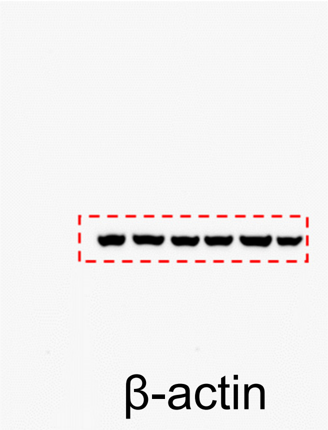
β-actin

Fig. S15L

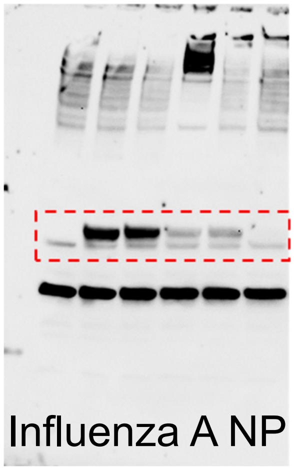
Influenza A NP

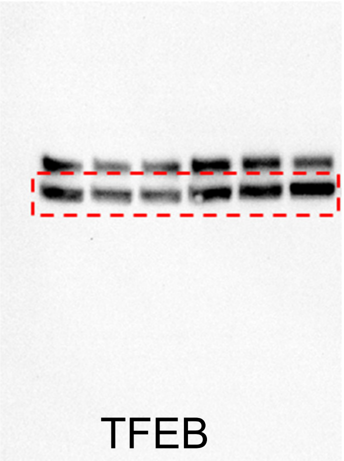
TFEB

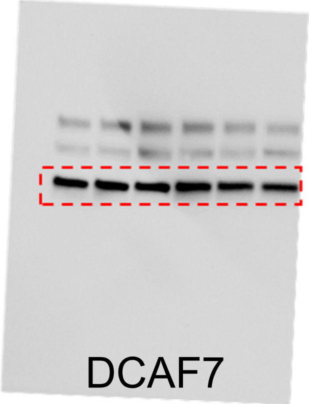
DCAF7

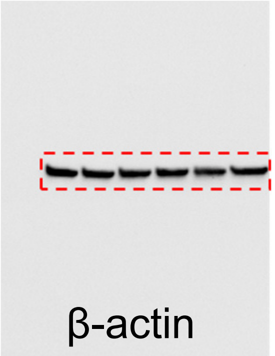
β-actin

Fig. S15A

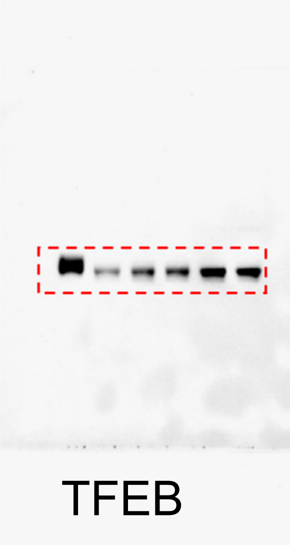
TFEB

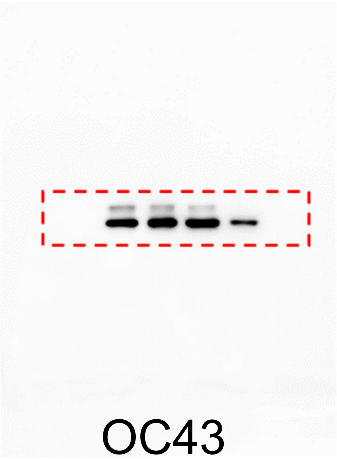
OC43
